# Effect of cadmium stress on certain physiological parameters, antioxidative enzyme activities and biophoton emission of leaves in barley (*Hordeum vulgare* L.) seedlings

**DOI:** 10.1371/journal.pone.0240470

**Published:** 2020-11-03

**Authors:** Ildikó Jócsák, Isaac Malgwi, Gyula Rabnecz, Anita Szegő, Éva Varga-Visi, György Végvári, Zsolt Pónya

**Affiliations:** 1 Szent István University, Kaposvár Campus, Faculty of Agricultural and Environmental Sciences, Institute of Plant Science, Kaposvár, Hungary; 2 Szent István University, Kaposvár Campus, Faculty of Agricultural and Environmental Sciences, Institute of Nutrition and Product Development Sciences, Kaposvár, Hungary; 3 PraxisLab. Ltd., Budapest, Hungary; 4 Szent István University, Institute of Horticultural Plant Biology, Department of Plant Biology and Plant Biochemistry, Budapest, Hungary; 5 Szent István University, Kaposvár Campus, Faculty of Agricultural and Environmental Sciences, Institute of Physiology, Biochemistry and Animal Health, Kaposvár, Hungary; Institute of medical research and medicinal plant studies, CAMEROON

## Abstract

Biophoton emission is a well-known phenomenon in living organisms, including plant species; however, the underlying mechanisms are not yet well elucidated. Nevertheless, non-invasive stress detection is of high importance when in plant production and plant research. Therefore, the aim of our work was to investigate, whether biophoton emission is suitable for the detection of cadmium stress in the early phase of stress evolution and to identify certain stress-related events that occur rapidly upon cadmium exposure of barley seedlings parallel to biophoton emission measurements. Changes of biophoton emission, chlorophyll content estimation index, ascorbate level, the activity of ascorbate- and guaiacol peroxidase enzymes and lipid oxidation were measured during seven days of cadmium treatment in barley (*Hordeum vulgare* **L**.) seedlings. The results indicate that the antioxidant enzyme system responded the most rapidly to the stress caused by cadmium and the lipid oxidation-related emission of photons was detected in cadmium-treated samples as early as one day after cadmium exposure. Furthermore, a concentration-dependent increase in biophoton emission signals indicating an increased rate of antioxidative enzymes and lipid oxidation was also possible to determine. Our work shows evidence that biophoton emission is suitable to identify the initial phase of cadmium stress effectively and non-invasively.

## Introduction

It has been long known that cells and tissues spontaneously emit photons in the UV to visible/near-infrared spectral range (approx. 350–1300 nm) of the electromagnetic spectrum [1]. This phenomenon is a form of chemiluminescence and is thought to derive from electronically excited species forming concomitantly upon oxidative stress or oxidative metabolic processes. This type of chemiluminescence is universal as it has been detected in almost every biological tissue examined hitherto, such as spinach chloroplasts [2], an aqueous extract of crude and roasted soybeans [3], plant leaves [4–6], azuki bean seedlings [7] and soybean seedlings [8]. The oxidation of biomolecules such as lipids, proteins and nucleic acids induces the formation of electronically excited species, which, following undergoing an electronic transition phase, return to ground state [1, 9]. This process is accompanied by photon emission, the ultra-low-level of which requires special devices (PMTs or CCDs) developed to detect extremely low optical signals from weakly emitting sources. This measurement can be carried out non-invasively, *in vivo*, that enables the possibility to implement consecutive measurements in plants at defined times [1, 9]. Earlier studies claim that biophoton emission (BPE) is closely related to stress-induced oxidation processes in plants [10]. Formation of reactive oxygen species (ROS) is a general phenomenon in plants [11, 12], also increased antioxidative enzyme activity ensuing upon stress posed by cadmium is well documented in the literature [9]. Thus, this phenomenon-dubbed BPE has been discussed in the context of radical reactions with particular regard to ROS generation and ROS-initiated cellular dysfunction. Reactive species, such as hydroxyl radicals, superoxide anion radical or hydrogen peroxide, oxidise the surrounding environment in which they were produced, mostly membrane structures and emit photons in the meantime [9]. The endogenous production of excited states during oxidative reactions leads to light-emission, which is mainly due to oxidation of lipids [13] producing light-emitting molecules such as triplet carbonyls and singlet oxygen. This spontaneous photon emission is ultra-weak, with an intensity in the order of 10^1^–10^4^ photons s^-1^ cm^-2^ and is considered to stem from the de-excitation of energetically excited species to a lower energy level in the course of which emission of photons concomitantly occurs. In their early work, Hideg and Inaba [2] reported that dark-adapted spinach chloroplasts emit ultra-weak light, even several hours after illumination and they proved that the production of superoxide radicals is associated with a series of reactions, leading to photoemission.

Heavy metal poisoning of plants is an important issue in plant physiology due to general environmental contamination throughout the whole ecosystem, mostly as a consequence of the human activity. The detoxification of heavy metals is a complex process with numerous elements [14]. Cadmium is one of the most harmful non-essential heavy metals that, regardless of its quantity, affects plants in a detrimental way by triggering severe disorders in plant functionality and structure [15]. In plant stress physiology, there is an increasing need for stress detection technologies with the help of which the physiological state of plants can be characterised with particular attention to non-invasiveness [16, 17].

BPE as a plant stress detecting tool has been used several times for high fertigation [4], leaf wound in *Spathiphyllum* [5] and in *Arabidopsis* [6], heat stress [7], flood [8] and osmotic stress [18]. Hossain et al. [10] measured BPE after five days of 100 μM cadmium treatment in soybean. Albeit BPE is a non-invasive technique for plant stress detection, to our knowledge, it has not been used thus far for the investigation of emerging cadmium toxicity in barley.

Therefore, the aim of this work was to investigate to what extent the different cadmium concentrations (10, 50, 100, 300 μM CdCl_2_) affect BPE arising from barley seedlings along with the measurement of plant-physiological parameters that are related to the physiological state of the plant and to ROS formation. The experiment was done in order to determine the BPE dynamics of cadmium stress evolution.

## Materials and methods

### Growth conditions

Barley (*Hordeum vulgare* L. ‘Triangel’) seeds were soaked and sterilised in 3% sodium hypochlorite for 3 minutes with constant stirring with a glass rod, then were rinsed thoroughly several times with distilled water and soaked for further 3 hours in distilled water. Seeds were then rinsed again with distilled water, and the water was discarded. Wet seeds were transferred onto the surface of germinating plates that were placed on 1.2 L containers in a way that the seed coats just touched the water, but they were not sunk. This was one precaution against drying out the seeds. The germinating plates were covered with plastic wrap, again, in order to prevent the seeds from drying. The seeds were then put into a phytotron growth chamber (Pol-Eco Apartura KK 1450) under the condition of 20°C; 120 μM m^-2^*•*s^-1^ light intensity and 12–12 h light/dark period, in order to provide a controlled environment for their growth under the whole duration of the experiment. The seeds germinated and raised for five days. Then, the plastic wrap was removed from the top of the germinating plates, and distilled water was changed to half-strength Hoagland solution [20] for five more days under the same growth conditions in the same growth chamber. The solution was renewed every second day for the purpose of normal root oxygen supply. After having spent ten days all together with germination and growth, seedlings were treated with cadmium.

### Sampling conditions

The data collection and analyses took place in sequential order. First, one pot from each treatment (0, 10, 50, 100 and 300 μM cadmium) was placed into the NightShade LB 985 *in vivo* Plant Imaging System, and biophoton emission measurement was conducted as it is described later in BPE measurement chapter. Then, pots were removed from the NightShade chamber, and SPAD index measurements were conducted on them, as it is detailed in SPAD index measurement (chlorophyll content estimation) chapter. Finally, the sampling conditions for the measurements requiring grinding of the leaf tissue (metal content determination, antioxidative enzyme activity, ascorbate and lipid oxidation measurement) were the following: half of the pots were cut (app. at least 20 leaves), rinsed with distilled water, dried with paper towel and cut into 0.5 cm wide pieces. Then, the leaf fragments were mixed in order to create an average sample of each individual treatment, so that the results could be considered as characteristic for the treatment.

### Cadmium treatment

Ten-day-old seedlings were treated with half-Hoagland solution containing CdCl_2_ (cadmium: 10 μM, 50 μM, 100 μM, 300 μM) for one week. Leaf tissue of the seedlings was sampled for lipid oxidation, antioxidative enzyme activity–and for heavy metal content determination after 0, 1, 3 and 7 days of the cadmium treatment. The experiment was repeated three times, mean values and standard deviations are presented.

### Cadmium content measurement

The cadmium contents of the leaves of barley seedlings were measured according to Hegedűs et al. [20] and expressed as μg/g d.w. 0.1 of the dried and powdered leaf was put into 10 mL of a 1:1 mixture of concentrated hydrogen peroxide and nitric acid. The complete digestion took place in teflon bombs at 12–16 h at room temperature and then by autoclaving at 120°C. 10 mL was filtered through S3 filter paper. Heavy metal content was determined with an ICAP-61E type plasma emission spectrometer.

### SPAD index measurement (chlorophyll content estimation)

SPAD index, as a non-invasive measurement for chlorophyll content estimation, was measured by reading ten individual points on ten seedlings of each treatment were measured with SPAD (Soil Plant Analysis Development–SPAD-502; Konica Minolta Sensing Inc, Japan) equipment. A barley leaf was app. 10–15 cm long, so measurements were taken along the full length of each leaves in app. every 1–1.5 cm. The reason for this was to get an overall, thus realistic picture of the leaf.

### Tissue extraction for antioxidative enzyme activity measurement

0.5 g of barley leaf tissue was homogenised in 1.5 mL of 0.1 M isolating buffer consisting of potassium-phosphate (1 M stock solution of 90.8 mL K_2_HPO_4_ and 9.2 mL 1 M stock solution of KH_2_PO_4_ (0.1 M K-P buffer (pH = 7.8), 1 mM phenylmethylsulfonyl fluoride-PMSF, 2 mM diethylenetriamine pentaacetic acid-DTPA, 1 mM 1,4-dithiothreitol-DTT, 5 mM ascorbate) in ice-cold mortar [19]. The suspension was centrifuged for 20 minutes, at 10000 g, 4°C. The supernatant was aliquoted into 1.5 mL Eppendorf tubes and used for enzyme activity measurements.

### Peroxidase (POX) (e.c.1.11.1.7.) assay

The catalysed reaction:

$$4\;\text{guaiacol}+4{\text{H}}_{2}{\text{O}}_{2}\to \text{tetraguaiacol}+8{\text{H}}_{2}\text{O}$$

The guaiacol peroxidase assay mixture contained 0.8 mL of 0.015 M H_2_O_2_ solution in 0.5 M phosphate buffer (pH 6.0), 0.5 mL of 0.02 M guaiacol solution and 1.6 mL distilled water. 0.1 mL of enzyme extract was added to the assay mixture in such a manner that the final volume was 3 mL. The absorption was determined against the blank solution (without enzyme) in 1 cm glass cuvettes on 470 nm (ε_470_ = 26.6 mM^-1^cm^-1^) for 1 minute at 25 °C using a BioRad SmartSpec^™^ Plus spectrophotometer [19]. The activity values were calculated as an average of three parallel measurements.

$$\text{Enzyme}\;\text{activity}(\mu \text{kat}/\text{g}\;\text{f.w.})\;\text{calculation}:(3\text{x}0.1^{-1})(1\text{x}26.6^{-1})(\Delta {\text{A}}_{1\text{min}}\text{x}60^{-1})(1.5\text{x}0.5^{-1})\;\text{x}\;4$$

### Ascorbate peroxidase (APX) (E.C. 1.11.1.11.) assay

The catalysed reaction:

$$2\;\text{ascorbate}+{\text{H}}_{2}{\text{O}}_{2}\to 2\;\text{monodehydroascorbate}+2{\;\text{H}}_{2}\text{O}$$

The ascorbate peroxidase mixture contained 1.5 mL of 0.1 M potassium phosphate buffer (pH 7.0), 0.5 mL of 3 mM ascorbate, 0.05 mM 0.015 M H_2_O_2_ solution and 0.85 mL distilled water. 0.1 mL of enzyme extract was added to the assay mixture, and the final volume was 3 mL. The absorption was determined against the blank solution (without extracted enzyme) in 1 cm quartz cuvettes on 290 nm (ε_290_ = 2.8 mM^-1^cm^-1^) for 1 minute on 25 °C on a BioRad SmartSpec^™^ Plus spectrophotometer [20]. The activity values were calculated as the average of three parallel measurements.

$$\text{Enzyme}\;\text{activity}\;(\mu \text{kat}/\text{g}\;\text{f.w.})\;\text{calculation}:(3\text{x}0.1^{-1})(1\text{x}2.8^{-1})(\Delta {\text{Ax}}_{1\text{min}}\text{x}60^{-1})(1.5\text{x}0.5^{-1})\;\text{x}\;4$$

### Determination of ascorbic acid (AA) content

0.5 g of leaves were homogenised in a cool mortar using quartz sand in 4.5 mL cooled (4°C) 0.1 M phosphate buffer (pH 7.6, containing 0.1 mM EDTA). This mixture was filtered through a filter paper and centrifuged at 15.000. rpm for 10 min. The supernatant was used for AA determination [21]. Determination of the AA content of barley samples based on the reduction of ferric ion to ferrous ion in the presence of AA, following the formation of a red-orange complex composed of α, α’-dipyridyl and ferrous ion [22]; a protocol used for the determination of AA level from various plant [23] and animal matrices [22]. A 0.15 cm^3^ aliquot of the sample was acidified with 0.5 cm^3^ 4.25 v/v % ortophosphoric acid solution, then 0.25 cm^3^ 1 w/v % α, α’-dipyridyl solution, 0.1 cm^3^ 1 w/v % ferric-chloride solution and 2 cm^3^ ultra-pure water were added. The solutions were mixed and allowed to stand at room temperature for 60 minutes. The absorbance of the sample was measured at 540 nm in a Shimadzu, UV-1800 spectrophotometer (Nishinokyo Kuwabara-cho, Nakagyo-ku, Kyoto 604–8511, Japan). The concentration was determined using calibration with AA standard solutions.

### Measurement of lipid oxidation

Malondialdehyde (MDA) content was determined by the thiobarbituric acid (TBA) reaction with some modifications of the original method [24]. Samples of 0.5 g were homogenized with 2 mL of 0.1% trichloroacetic acid (TCA) in cold mortars from which 1.8 mL was transferred to Eppendorf tubes with automatic pipettes. To this solution, 40 μL of 20% butylated hydroxytoluene (BHT) in absolute ethanol was added in order to stop further lipid oxidation. The solutions were vortexed for 15 s and centrifuged at 13,000 rpm for 10 min at 4°C. From the clear supernatant 0.25 mL was added to 1 mL of 20% TCA containing 0.5% TBA, gently mixed and briefly centrifuged for 5 s. The solutions were incubated in a water bath (Julabo ED-5M) for 30 min at 96°C. The reactions were stopped by cooling the solutions immediately on ice followed by centrifugation at 10,000 rpm for 5 min. Absorbance at 532 and 600 nm was recorded using a SmartSpec^™^ Plus spectrophotometer, and MDA concentration was calculated by subtracting the non-specific absorption at 600 nm from the absorption at 532 nm using an absorbance coefficient of extinction, 156 mM^-1^ cm^-1^. The results were expressed as nmol g^-1^ in fresh weight (f.w.).

### BPE imaging

For measuring BPE the NightShade LB 985 *in vivo* Plant Imaging System (Berthold Technologies GmbH & Co.KG, 75323 Bad Wildbad, Germany) equipped with a sensitive, thermoelectrically cooled slow-scan NighOwlcam CCD device has been employed. The instrument was controlled by the IndiGo^™^ 2.0.5.0. software. Intensities of light were converted into counts per second (cps) by using the controlling software. The exposure time was kept at 60 sec using a pixel binning of 2 x 2. In the duration of taking the images both the “background correction” and the „cosmic suppression” options were enabled to ensure the elimination of high intensity pixels potentially caused by cosmic radiation. One pot for each treatment (0, 1, 3 and 7 days after cadmium treatment) of the seedlings to be imaged was placed into the dark imaging box and subsequently dark-adapted for 5 min in order to avoid chlorophyll-autofluorescence deriving from electron recoupling of the photosystems.

### Statistical analysis

The presented values were plotted as an average of three independent experiments and presented together with standard deviations (±SD). The effect of the treatments on the measured variables was determined by ANOVA (p≤0.05), Duncan and Tamhane tests, SPSS 7.0 statistical program.

## Results

**Fig 1:**
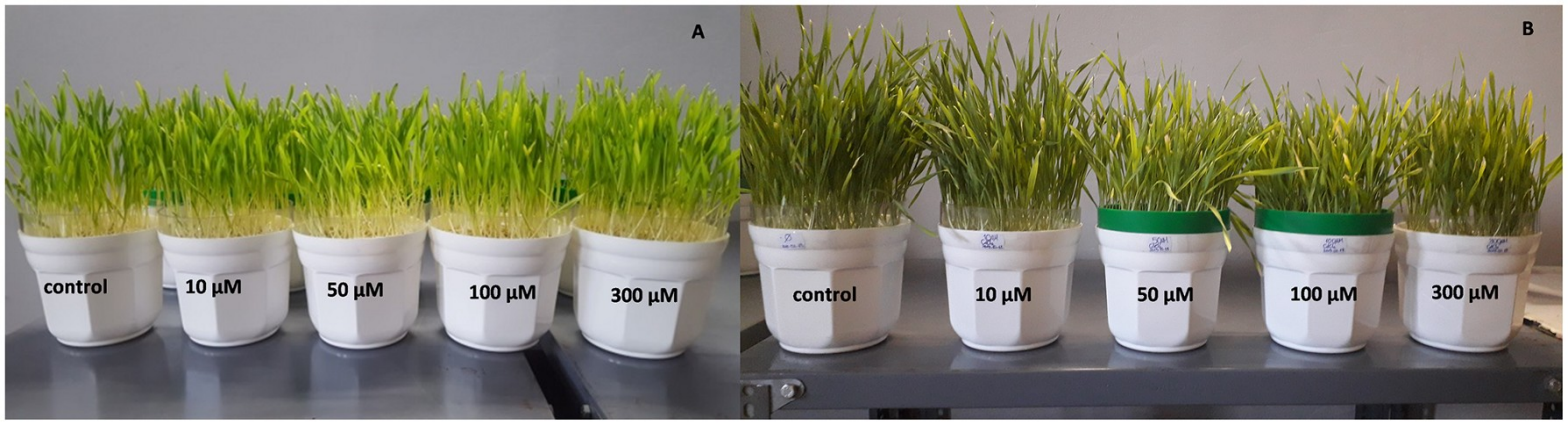
Cadmium-treated barley (*Hordeum vulgare* L.) seedlings. A: on the day of treatment (0^th^ day); B: on the 7^th^ day of treatment. The cadmium concentrations of each treatment are indicated on the images.

The images presented in Fig 1 show the effect of cadmium treatment on the growth of barley seedling. The length of the leaves of cadmium treated seedlings remained the same after the cadmium treatment when higher (50, 100, 300 μM) cadmium concentrations were applied (Fig 1). The growth of the latter seedlings literally stopped as a result of cadmium presence.

### Cadmium content of leaves of barley seedlings

The pattern of cadmium accumulation was continuous and concentration-dependent throughout the experiment. On the first day of cadmium treatment, the cadmium content of the leaves was significantly different from the control in all the treatments.

**Fig 2:**
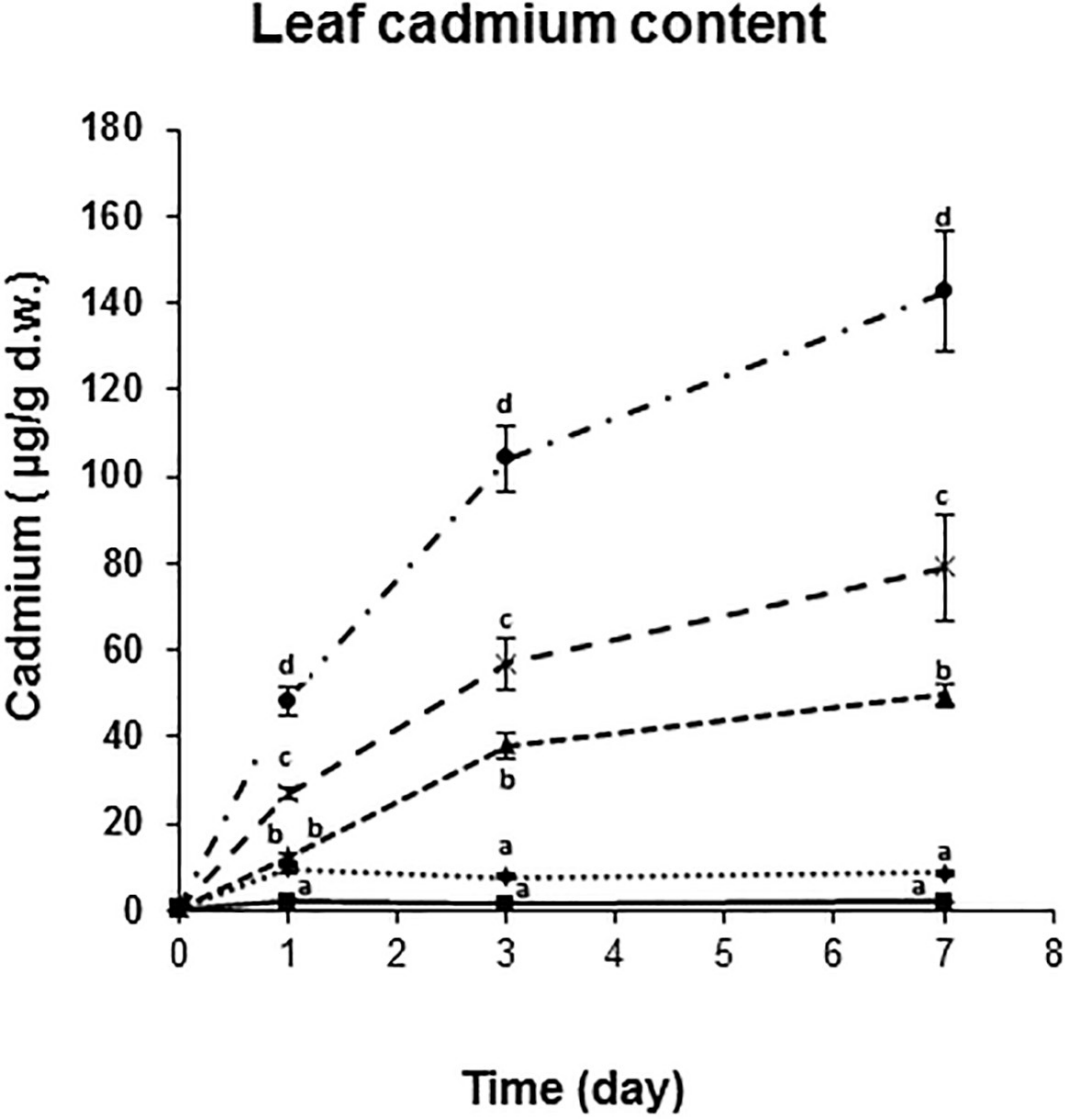
Cadmium content of leaves of barley seedlings. Mean values (n = 3) and ± standard deviations are presented. The same letters represent non-significant difference within each treatment.

On the 3^rd^ and 7^th^ days 50, 100 and 300 μM treatments significantly differed from each other and from the control, and for the highest concentration, the cadmium content of the leaves reached 140 times increase compared to the control (Fig 2).

### The effect of cadmium on SPAD index

SPAD index reflects the chlorophyll content of the leaves. In the presented work, cadmium treatment resulted lower levels in the SPAD indices in a concentration-dependent way.

The effect of the level of cadmium treatment was not detectable in the first two sampling dates, although from the 3^rd^ day there was a distinct, well-characterised, statistically significant decrease in the SPAD index parallel with the increase of cadmium level. On the 3^rd^ day, the 50, 100 and 300 μM treatments were significantly different from 0 and 10 μM respectively. Also the two latter differed from one another significantly. By the last sampling time of the experiment, all concentrations of cadmium applied were separated statistically significantly from each other (Table 1).

**Table 1:** Effect of cadmium on SPAD index in barley (*Hordeum vulgare* L.) seedlings.

Effect of cadmium treatment on the SPAD index in barley leaves				
	days			
	0	1	3	7
**0 μM**	28.039±1.57^A,a^	29.534±1.74^B,b^	27.159±1.77^Ac^	30.219±2.35^B,e^
**10 μM**	28.039±1.57^BC,a^	28.779±1.71^C,ab^	24.981±2.27^A,b^	26.392±2.52^AB,d^
**50 μM**	28.039±1.57^B,a^	28.545±2.13^B,ab^	21.059±1.78^A,a^	20.395±3.16^A,c^
**100 μM**	28.039±1.57^C,a^	27.558±1.41^C,a^	20.567±1.6^B,a^	17.012±3.59^A,b^
**300 μM**	28.039±1.57^C,a^	28.522±2.16^C,ab^	20.896±1.5^B,a^	14.616±3.68^A,a^

Mean values (n = 100) and ± standard deviations are presented. The same letters represent non-significant difference within each treatment. Upper case letters refer to time effect, whereas lower case letters letter refer to the effect of the level of cadmium treatment.

The effect of the length of the treatment manifested in significant SPAD index decrease of 10, 50, 100 and 300 μM cadmium-treated seedlings (Table 1). In 50, 100 and 300 μM cadmium-treated seedlings the statistical differentiation occurred between the first two and the last two sampling occasions.

### The effect of cadmium on antioxidative enzyme activity and ascorbate level

#### Guaiacol peroxidase (EC 1.11.1.7.)

One-day cadmium treatment resulted in significant increase in POX activity in the 100 and 300 μM treatments compared to the control. On the 3^rd^ day, 50, 100 and 300 μM treatments were significantly higher than the control, and on the 7^th^ day, POX values were significantly higher in all the applied concentrations, than the control (Table 2).

**Table 2:** Effect of cadmium on peroxidase activity (μkat/g f.w.) in barley (*Hordeum vulgare* L.) seedlings.

Effect of cadmium treatment on peroxidase activity in barley leaves				
	days			
	0	1	3	7
**0 μM**	0.4±0.01^AB,a^	0.36±0.05^B,a^	0.64±0.12^AB,a^	0.53±0.044^A,a^
**10 μM**	0.4±0.01^A,a^	0.42±0.10^AB,a^	0.9±0.32^BC,a^	1.25±0.12^C,b^
**50 μM**	0.4±0.01^A,a^	0.56±0.03^A,ab^	1.5±0.21^B,b^	2.42±0.22^C,c^
**100 μM**	0.4±0.01^A,a^	0.84±0.22^A,bc^	1.93±0.18^B,c^	2.23±0.13^C,c^
**300 μM**	0.4±0.01^A,a^	0.95±0.28^A,c^	2.7±0.50^B,d^	3.25±0.15^C,d^

Mean values (n = 3) and ± standard deviations are presented. The same letters represent non-significant difference within each treatment. Upper case letters refer to time effect, whereas lower case letters letter refer to the effect of the level of cadmium treatment.

The effect of the time on cadmium-initiated POX activity manifested in higher POX activities over time. The 3^rd^ and 7^th^ day of 10 μM treatment showed a significantly higher POX, than the 0 day value. In the 50, 100 and 300 μM treatments, the POX values of the 3^rd^ and 7^th^ days were significantly higher than that of 1^st^ and 0 days. Also, the POX values of 100 and 300 μM treatments were significantly different from one another (Table 2).

#### Ascorbate peroxidase (EC 1.11.1.11.)

Cadmium treatment resulted in significant increase in APX activity in all of the applied levels on the 1^st^ day. The distinction of treatments continued, since on the 3^rd^ day, the APX activates of 100, and 300 μM treatments were significantly higher than 0 and 10 μM treatments and by the 7^th^ day APX activities of all treatments were significantly higher than that of the control, with a significant difference between 300 μM treatment and the others (10, 50 and 100 μM). (Table 3).

**Table 3:** Effect of cadmium on ascorbate peroxidase activity (μkat/g f.w.) in barley (*Hordeum vulgare* L.) seedlings.

Effect of cadmium treatment on ascorbate peroxidase activity in barley leaves				
	days			
	0	1	3	7
**0 μM**	0.14±0.1^A,a^	0.13±0.01^A,a^	0.14±0.02^A,a^	0.14±0.02^A,a^
**10 μM**	0.14±0.1^A,a^	0.16±0.01^AB,b^	0.18±0.01^CB,b^	0.21±0.03^C,b^
**50 μM**	0.14±0.1^A,a^	0.16±0.02^A,b^	0.20±0.02^B,bc^	0.24±0.03^C,b^
**100 μM**	0.14±0.1^A,a^	0.16±0.01^A,b^	0.22±0.01^B,c^	0.26±0.02^B,b^
**300 μM**	0.14±0.1^A,a^	0.18±0.01^B,b^	0.26±0.03^C,d^	0.33±0.02^D,c^

Mean values (n = 3) and ± standard deviations are presented. The same letters represent non-significant difference within each treatment. Upper case letters refer to time effect, whereas lower case letters refer to the effect of the level of cadmium treatment.

The effect of the time on cadmium-initiated APX activity manifested in higher activity values. The 3^rd^ and 7^th^ day of 10 μM treatment showed a significantly higher APX, than the 0 day value. In the 50, 100 and 300 μM treatments, 3^rd^ and 7^th^ days were significantly different from 0 and 1^st^ days, although the values of 0 and 1^st^ days did not differ significantly in 50 and 100 μM treatments and the 300 μM cadmium treatment resulted in significantly higher APX values in all sampling days compared the day of cadmium treatment (Table 3).

#### Ascorbate level

Cadmium treatment resulted in a significant increase in AA level in the 1^st^ sampling day of the 100 and 300 μM treatment compared to the control (Table 4). On the 3^rd^ day of 100 and 300 μM treatments resulted in significantly higher AA levels compared to that of control, 10 and 50 μM. On the 7^th^ day, 300 μM treatments gave significantly higher AA levels than 10 μM and the untreated (Table 4).

**Table 4:** Effect of cadmium on ascorbate level (mg/g f.w.) in barley (*Hordeum vulgare* L.) seedlings.

Effect of cadmium treatment on ascorbate level				
	days			
	0	1	3	7
**0 μM**	0.54±0.01^A,a^	0.56±0.08^A,a^	0.77±0.01^A,b^	0.88±0.18^A,a^
**10 μM**	0.54±0.01^A,a^	0.59±0.01^A,ab^	0.67±0.03^A,a^	0.94±0.16^A,a^
**50 μM**	0.54±0.01^A,a^	0.64±0.01^A,ab^	0.82±0.02^A,b^	1.3117±0.10^B,ab^
**100 μM**	0.54±0.01^A,a^	0.79±0.01^A,b^	0.93±0.01^AB,c^	1.2965±0.10^B,ab^
**300 μM**	0.54±0.01^A,a^	1.15±0.01^A,c^	0.98±0.02^A,c^	1.79±0.10^B,b^

Mean values (n = 3) and ± standard deviations are presented. The same letters represent non-significant difference within each treatment. Upper case letters refer to time effect, whereas lower case letters letter refer to the effect of the level of cadmium treatment.

The effect of time on cadmium-initiated AA level change was not significant in the control and in the 10 μM treatment. However, the AA level by the 7^th^ day of cadmium treatment was significantly higher than in the other days for 50 and 300 μM treatments, as well (Table 4).

### The effect of cadmium on lipid oxidation

The different cadmium treatments did not initiate significant changes in the lipid oxidation within one day, while the 300 μM cadmium treatment resulted in significantly higher MDA On the 7^th^ day 0, 10–50 and 100–300 μM treatments significantly differed from each other and the higher the concentration was; the higher lipid oxidation occurred (Table 5).

**Table 5:** Effect of cadmium on lipid oxidation (nM/g f.w. MDA equivalent) in barley (*Hordeum vulgare* L.) leaves.

Effect of cadmium treatment on lipid oxidation				
	days			
	0	1	3	7
**0 μM**	20.2±0.89^A,a^	20.4±1.77^A,a^	18.03±2.02^A,a^	23.98±1.15^A,a^
**10 μM**	20.2±0.89^A,a^	19.11±6.79^A,a^	18.89±0.45^A,a^	31.47±1.33^B,b^
**50 μM**	20.2±0.89^B,a^	18.82±0.33^A,a^	18.31±2.0^A,a^	33.14±0.76^C,b^
**100 μM**	20.2±0.89^A,a^	20.54±1.0^A,a^	19.62±3.60^A,a^	48.99±7.30^B,c^
**300 μM**	20.2±0.89^A,a^	20.52±1.02^A,a^	25.01±0.72^B,b^	54.73±0.76^C,c^

Mean values (n = 3) and ± standard deviations are presented. The same letters represent non-significant difference within each treatment. Capital letters refer to time effect and normal letter refer to the effect of cadmium treatment.

By the end of the experiment, the effect of time acted on lipid oxidation state of seedlings in every applied concentrations; compared to 0 day. Furthermore, in case of the highest concentration, the lipid oxidation of the 3^rd^ and 7^th^ days significantly differed from one another and also from the other concentrations (Table 5).

### The effect of cadmium on BPE

**Fig 3:**
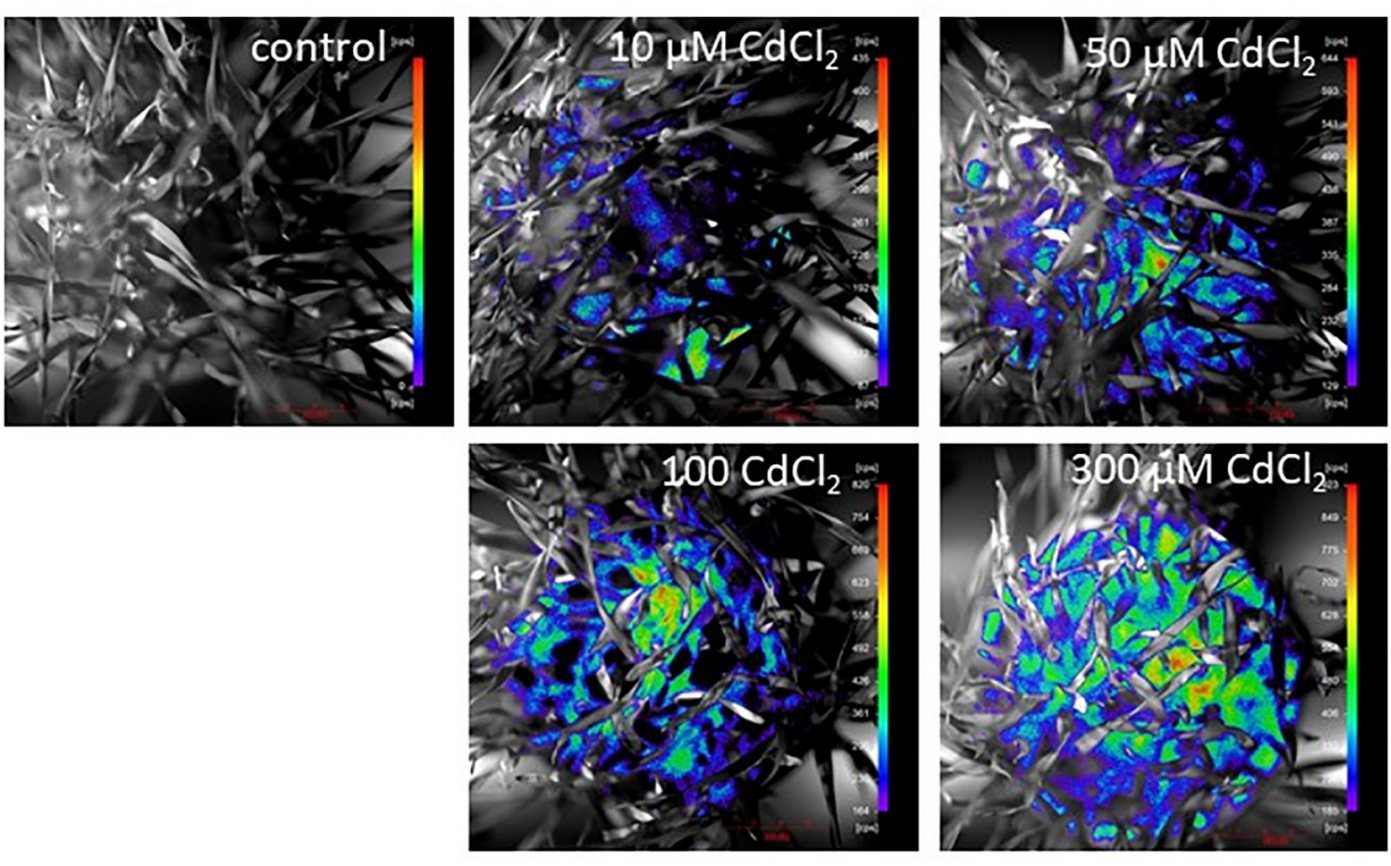
BPE of 17 days-old cadmium treated barley seedlings that were kept on cadmium containing half-Hoagland solution for 7 days. The applied treatments were: control, 10, 50, 100, 300 μM CdCl_2_. The intensity colour bar shows signal intensity detected by the equipment and converted into a scale of colour intensity via the software.

BPE varied according to the cadmium concentration and in response to the time elapsed after it was added to the growth medium. The control plants did not show remarkable BPE. The cadmium treated barley seedling, however showed an incremental increase in BPE (Fig 3) in a concentration-dependent way.

The colour bars represent pseudo-colour coded pixel intensity values in the image (Fig 3); the more intensive the photon emission is, the more red pseudo-colour coded pixels appear in the image. The emission occurs and becomes more intense after cadmium treatment in a concentration-dependent way, with the highest signal intensity observable among the seedlings treated with 300 μM CdCl_2_.

The average peak intensity was selected as a parameter for the interpretation of BPE results based on the work of Hennecke and Brüx [25]. The sum of the average peak intensity of cadmium treated barley seedlings showed a profound change during the experiment.

At the starting point of the cadmium treatment, the seedlings showed very low signs of BPE. This indicates that there were no other factors that initiated BPE other than cadmium. This statement is further verified by the observation that control plants also released low amount of photons on the sampling dates. Cadmium treatment, however, resulted in growing intensity of BPE even in the case of the treatment in which the lowest concentration (10 μM cadmium) was used.

**Fig 4:**
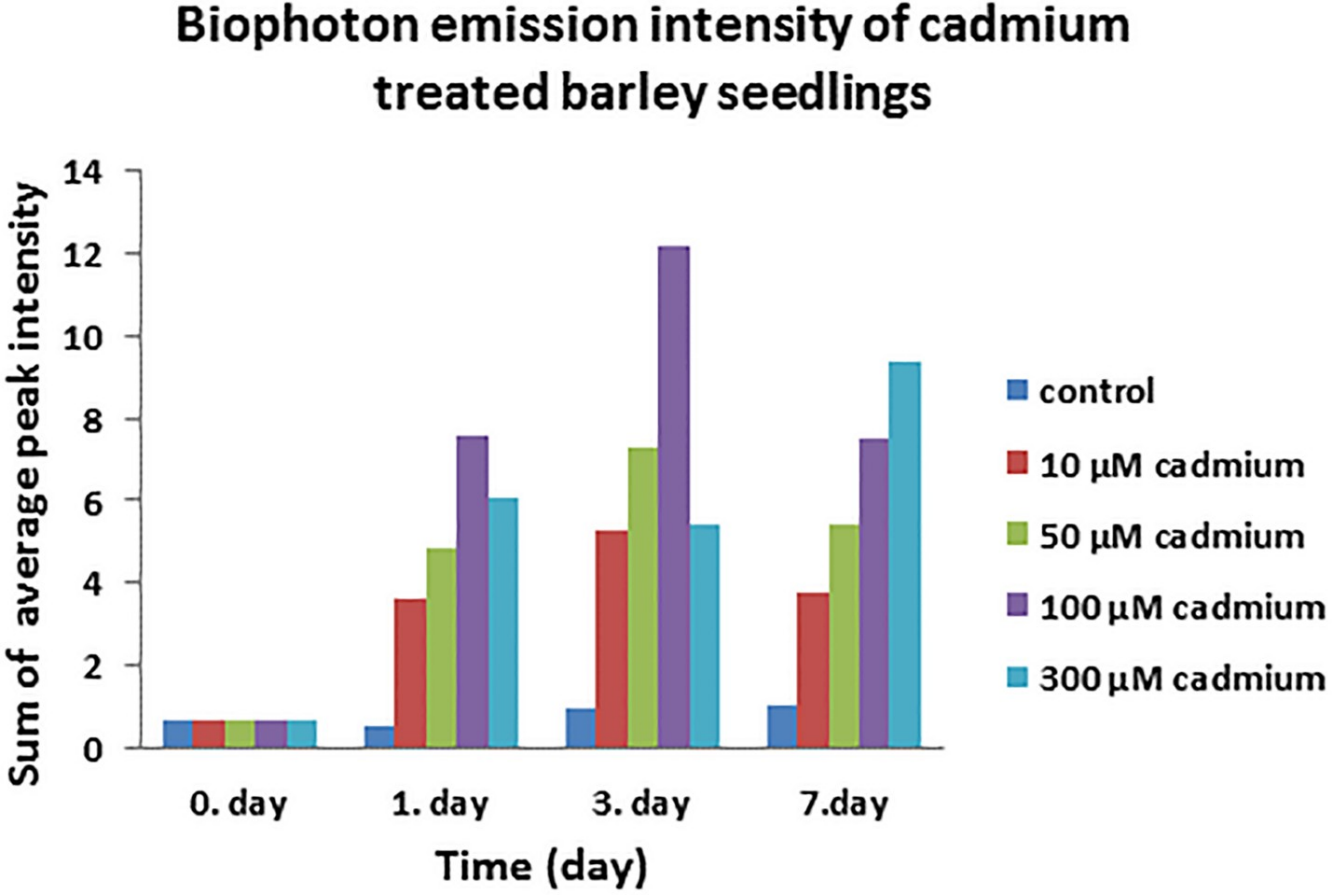
The sum of average BPE intensity [21] of 17 days-old cadmium treated barley seedlings that were kept on cadmium containing half-Hoagland solution for 7 days and BPE was measured on the 1^st^, 3^rd^ and 7^th^ day of cadmium treatment.

From the first day after cadmium treatment, all the cadmium-treated plants presented considerably higher BPE, than the untreated ones. By the end of the experiment, the cadmium treatment manifested a 4–10 times rise of BPE compared to the control seedlings (Fig 4).

## Discussion

The experimental design and the methods of investigations were chosen to support the idea that the effect caused by cadmium presence in barley seedlings generates BPE. Since BPE measurement is a new non-invasive investigational tool in the hands of plant physiologists, the conformation of the effect of cadmium was crucially important that was directly confirmed by the element content measurement. The activity of antioxidative enzymes, the level of MDA and ascorbate supplies characterisation of the physiological state of the plant, since POX is localised in the cell wall, the cytosol, the vacuole and the apoplast, while APX is located in chloroplasts, mitochondria, glyoxysomes and peroxisomes, moreover ascorbate is the substrate of APX and a non-enzymatic antioxidant in the same time [26].

The presented results prove that cadmium induces several metabolic changes, but from the perspective of BPE, the most relevant results are the ones related to ROS formation.

The antioxidant enzyme system of seedlings responded the most rapidly to the stress caused by cadmium, followed by an increase in ascorbate; as being the substrate of APX; levels involved in both enzymatic and non-enzymatic defense systems. AA plays a role in the elimination of superoxide and hydroxyl radicals as a non-enzymatic antioxidant. Also, Tóth et al. [27] proved that AA is an alternative electron donor for PSII, in case of heat stress that damages membranes and similar processes may occur in cadmium-stressed barley as well.

With the exception of the highest cadmium dose, BPE followed a maximum curve as a function of time, while MDA levels increased significantly only at the very end of the time interval studied. These time-dependent changes are analogous to the formation kinetics of the primary and secondary products of lipid oxidation measured in static systems, as the amount of lipid peroxides in these systems changes according to a maximum curve as a function of time, followed by the increase in the amount of secondary products (e.g. MDA) [28]. The phenomenon of BPE can be associated with reactions coupled with the formation and degradation of lipid peroxides [9, 13]. Hence, it may be used to characterise the intensity of the first phase (the so-called peroxide phase) in a non-destructive way and may be a consequence of the different cadmium concentration. 300 μM treatment resulted in a very intensive cadmium accumulation (Fig 2) with a steeply increasing manner as opposed to the other treatments, where the rise was not so intense, or even a plateau was reached. This indicates and further confirmed by the constantly rising BPE signals of 300 μM treated seedlings that they remained in the alarm phase of the stress when there is an overall increase in metabolism in order to be able to cope with the stress.

Overloading the antioxidant system first led to the increase in the amount of lipid peroxides–since in the first phase of peroxide formation the rate of peroxide formation is higher than the rate of peroxide-decomposition [28]; resulting in the enhanced intensity of BPE–inducing reactions. Nevertheless, at a later stage in the lipid oxidation, the amount of lipid peroxides and the BPE associated with them may be decreased, which may be attributable to the instability of lipid peroxides in the advanced phase of lipid oxidation suggesting a higher rate of degradation than that of formation [28] leading to dwindling lipid peroxide concentration. In parallel, the amount of compounds of the decomposition of peroxides increased, which is supported by our observation that significant MDA level increase was detected only following the peak of BPE.

In our study, BPE was higher in cadmium-treated samples than in controls as early as one day after the application of cadmium. Via promoting superoxide anion formation, cadmium also forms a compound that directly initiates lipid oxidation through the electron transport chains of the chloroplast and the mitochondrion. The decomposition of H_2_O_2_ deriving from superoxide anion by SOD also initiates the process [9]. Nonetheless, if BPE correlates directly with ROS levels, then the rapid increase in BPE can be explained by cadmium being a stressor, thereby causing increased ROS levels before the membrane lipids would suffer significant damage.

Since BPE is mostly associated with lipid hydroperoxides being the primary products of lipid oxidation, then, based on our findings, it can be conjectured that lipid oxidation was so rapid that it resulted in the emission of photons detectable by our sensitive CCD-sensor in cadmium-treated samples in as little as one day.

## Conclusions

The analysis of the data revealed that increasing cadmium concentrations initiate gradual and proportional changes in the determined stress indicator parameters.

Our work shows evidence pertaining to lipid oxidation caused by cadmium and supports previous findings demonstrating that BPE is linked to these processes and can be followed by BPE-detection effectively and non-invasively.

We demonstrate here that BPE is suitable for the confirmation of the presence of cadmium in the leaves of barley seedlings; however, more data are to be generated in order to establish its dynamics. A future task would be to fine-tune the methodology in order to provide a more detailed characterisation of the relationship between cadmium stress and BPE. Since BPE changed during one day of cadmium exposure, it is advisable to investigate the spatio-temporal dynamics that depicts the cellular processes induced by within one-day cadmium treatment as well.

## Supporting information

S1 Data (XLSX)

S2 Data (XLSX)

S1 File (ZIP)
