# Supplementary material for: Effect of cadmium stress on certain physiological parameters, antioxidative enzyme activities and biophoton emission of leaves in barley (Hordeum vulgare L.) seedlings
Source: PLoS One. 2020 Nov 3;15(11):e0240470. doi: 10.1371/journal.pone.0240470 (PMC7608874; doi:10.1371/journal.pone.0240470)
Supplement: S1 File — (ZIP) [file pone.0240470.s003.zip › stat result time-0 Cd AA leaf.pdf]

```
ONEWAY AA1 BY Idő
  /STATISTICS DESCRIPTIVES HOMOGENEITY
  /MISSING ANALYSIS
  /POSTHOC=DUNCAN T2 ALPHA(0.05).
```

Oneway

[DataSet2] H:\Jócsák\01 Növényélettan\árpa vizsgálatok\PhD téma folytatása \Visi É árpa c vit meghatározás\aszkorbinsav mg-g fr tömeg.sav

Descriptives

AA1

	N	Mean	Std. Deviation	Std. Error	95% Confidence Interval for Mean	
					Lower Bound	Upper Bound
1	2	,5619	,16949	,11985	-,9610	2,0847
3	2	,7659	,00948	,00670	,6808	,8510
7	2	,8805	,36678	,25935	-2,4149	4,1758
Total	6	,7361	,23131	,09443	,4933	,9788

Descriptives

AA1

	Minimum	Maximum
1	,44	,68
3	,76	,77
7	,62	1,14
Total	,44	1,14

Test of Homogeneity of Variances

AA1

Levene Statistic	df1	df2	Sig.
.	2	.	.

ANOVA

AA1

	Sum of Squares	df	Mean Square	F	Sig.
Between Groups	,104	2	,052	,957	,477
Within Groups	,163	3	,054		
Total	,268	5			

Post Hoc Tests

Multiple Comparisons

Dependent Variable: AA1

	(I) Idő	(J) Idő	Mean Difference (I-J)	Std. Error	Sig.	95% Confidence Interval	
						Lower Bound	Upper Bound
Tamhane	1	3	-,20405	,12004	,709	-4,6157	4,2076
		7	-,31860	,28570	,804	-4,4164	3,7792
	3	1	,20405	,12004	,709	-4,2076	4,6157
		7	-,11455	,25944	,981	-9,8105	9,5814
	7	1	,31860	,28570	,804	-3,7792	4,4164
		3	,11455	,25944	,981	-9,5814	9,8105

Homogeneous Subsets

AA1

	Idő	N	Subset for alpha = 0.05
			1
Duncan[a]	1	2	,5619
	3	2	,7659
	7	2	,8805
	Sig.		,264

Means for groups in homogeneous subsets are displayed.

a. Uses Harmonic Mean Sample Size = 2,000.
