# Supplementary material for: Effect of cadmium stress on certain physiological parameters, antioxidative enzyme activities and biophoton emission of leaves in barley (Hordeum vulgare L.) seedlings
Source: PLoS One. 2020 Nov 3;15(11):e0240470. doi: 10.1371/journal.pone.0240470 (PMC7608874; doi:10.1371/journal.pone.0240470)
Supplement: S1 File — (ZIP) [file pone.0240470.s003.zip › stat result time-0 Cd MDH-enzyme leaf.pdf]

```
GET
  FILE='H:\Jócsák\01 Növényélettan\árpa vizsgálatok\PhD téma folytatása\Vi
si É árpa c vit meghatározás\aszkorbinsav mg-g fr tömeg.sav'.
DATASET NAME DataSet3 WINDOW=FRONT.
DATASET ACTIVATE DataSet2.
ONEWAY MDHlevél GPXlevél APXlevél GRlevél BY Idő
  /STATISTICS DESCRIPTIVES HOMOGENEITY
  /MISSING ANALYSIS
  /POSTHOC=DUNCAN T2 ALPHA(0.05).
```

Oneway

[DataSet2] H:\Jócsák\01 Növényélettan\árpa vizsgálatok\PhD téma folytatása
\MGHgyökér_1.sav

Descriptives

		N	Mean	Std. Deviation	Std. Error	95% Confidence ... Lower Bound
MDHlevél	0	3	21,5415	1,68115	,97061	17,3652
	1	3	20,4009	1,77390	1,02416	15,9943
	3	3	18,0267	2,02616	1,16981	12,9934
	7	3	23,9871	1,15389	,66620	21,1207
	Total	12	20,9890	2,66398	,76903	19,2964
GPXlevél	0	3	,6837	,04569	,02638	,5702
	1	3	,7886	,08550	,04936	,5762
	3	3	,6393	,11548	,06667	,3525
	7	3	,5299	,04374	,02525	,4213
	Total	12	,6604	,11780	,03401	,5855
APXlevél	0	3	,1575	,01006	,00581	,1325
	1	3	,1626	,01112	,00642	,1349
	3	3	,1396	,02043	,01180	,0889
	7	3	,1412	,01978	,01142	,0921
	Total	12	,1502	,01722	,00497	,1393
GRlevél	0	3	,004596	,0009166	,0005292	,002319
	1	3	,004774	,0007611	,0004394	,002884
	3	3	,005094	,0008244	,0004760	,003046
	7	3	,005450	,0011863	,0006849	,002503
	Total	12	,004979	,0008679	,0002505	,004427

Descriptives

		95% Confidence ... Upper Bound	Minimum	Maximum
MDHlevél	0	25,7177	19,60	22,59
	1	24,8075	19,17	22,43
	3	23,0599	16,35	20,28
	7	26,8535	23,11	25,30
	Total	22,6816	16,35	25,30
GPXlevél	0	,7972	,64	,73
	1	1,0010	,70	,86
	3	,9262	,53	,76
	7	,6386	,49	,57
	Total	,7352	,49	,86
APXlevél	0	,1825	,15	,17
	1	,1902	,15	,17
	3	,1904	,12	,16
	7	,1904	,13	,16
	Total	,1612	,12	,17
GRlevél	0	,006873	,0037	,0055
	1	,006665	,0040	,0055
	3	,007142	,0042	,0058
	7	,008397	,0046	,0068
	Total	,005530	,0037	,0068

Test of Homogeneity of Variances

	Levene Statistic	df1	df2	Sig.
MDHlevél	,540	3	8	,668
GPXlevél	1,127	3	8	,394
APXlevél	,883	3	8	,490
GRlevél	,465	3	8	,715

ANOVA

		Sum of Squares	df	Mean Square	F	Sig.
MDHlevél	Between Groups	55,245	3	18,415	6,456	,016
	Within Groups	22,820	8	2,852		
	Total	78,065	11			
GPXlevél	Between Groups	,103	3	,034	5,591	,023
	Within Groups	,049	8	,006		
	Total	,153	11			
APXlevél	Between Groups	,001	3	,000	1,541	,277
	Within Groups	,002	8	,000		
	Total	,003	11			
GRlevél	Between Groups	,000	3	,000	,484	,703
	Within Groups	,000	8	,000		
	Total	,000	11			

Post Hoc Tests

Multiple Comparisons

Dependent Variable		(I) Idő	(J) Idő	Mean Difference (I-J)	Std. Error	Sig.	95% ... Lower Bound
MDHlevél	Tamhane	0	1	1,14056	1,41103	,976	-5,6770
			3	3,51479	1,52004	,410	-3,9957
			7	-2,44564	1,17725	,521	-8,6815
		1	0	-1,14056	1,41103	,976	-7,9581
			3	2,37423	1,55478	,743	-5,2169
			7	-3,58620	1,22177	,271	-10,2288
		3	0	-3,51479	1,52004	,410	-11,0253
			1	-2,37423	1,55478	,743	-9,9653
			7	-5,96043	1,34620	,109	-13,8303
		7	0	2,44564	1,17725	,521	-3,7902
			1	3,58620	1,22177	,271	-3,0564
			3	5,96043	1,34620	,109	-1,9095
GPXlevél	Tamhane	0	1	-,10495	,05597	,638	-,4446
			3	,04435	,07170	,995	-,4749
			7	,15375	,03652	,079	-,0226
		1	0	,10495	,05597	,638	-,2347
			3	,14930	,08296	,629	-,2761
			7	,25870	,05545	,108	-,0866
		3	0	-,04435	,07170	,995	-,5637
			1	-,14930	,08296	,629	-,5747
			7	,10940	,07129	,803	-,4194
		7	0	-,15375	,03652	,079	-,3301
			1	-,25870	,05545	,108	-,6040
			3	-,10940	,07129	,803	-,6382

Multiple Comparisons

Dependent Variable		(I) Idő	(J) Idő	95% ... Upper Bound
MDHlevél	Tamhane	0	1	7,9581
			3	11,0253
			7	3,7902
		1	0	5,6770
			3	9,9653
			7	3,0564
		3	0	3,9957
			1	5,2169
			7	1,9095
		7	0	8,6815
			1	10,2288
			3	13,8303
GPXlevél	Tamhane	0	1	,2347
			3	,5637
			7	,3301
		1	0	,4446
			3	,5747
			7	,6040
		3	0	,4749
			1	,2761
			7	,6382
		7	0	,0226
			1	,0866
			3	,4194

Multiple Comparisons

Dependent Variable		(I) Idő	(J) Idő	Mean Difference (I-J)	Std. Error	Sig.	95% ...
							Lower Bound
APXlevél	Tamhane	0	1	-,00504	,00866	,995	-,0471
			3	,01788	,01315	,848	-,0659
			7	,01628	,01281	,876	-,0638
		1	0	,00504	,00866	,995	-,0370
			3	,02292	,01343	,704	-,0577
			7	,02132	,01310	,734	-,0558
		3	0	-,01788	,01315	,848	-,1017
			1	-,02292	,01343	,704	-,1035
			7	-,00160	,01642	1,000	-,0808
		7	0	-,01628	,01281	,876	-,0964
			1	-,02132	,01310	,734	-,0985
			3	,00160	,01642	1,000	-,0776
GRlevél	Tamhane	0	1	-,0001786	,0006879	1,000	-,003577
			3	-,0004984	,0007118	,988	-,003958
			7	-,0008545	,0008655	,945	-,005224
		1	0	,0001786	,0006879	1,000	-,003219
			3	-,0003198	,0006478	,998	-,003457
			7	-,0006759	,0008137	,975	-,005131
		3	0	,0004984	,0007118	,988	-,002961
			1	,0003198	,0006478	,998	-,002818
			7	-,0003561	,0008340	,999	-,004749
		7	0	,0008545	,0008655	,945	-,003515
			1	,0006759	,0008137	,975	-,003779
			3	,0003561	,0008340	,999	-,004037

Multiple Comparisons

Dependent Variable		(I) Idő	(J) Idő	95% ... Upper Bound
APXlevél	Tamhane	0	1	,0370
			3	,1017
			7	,0964
		1	0	,0471
			3	,1035
			7	,0985
		3	0	,0659
			1	,0577
			7	,0776
		7	0	,0638
			1	,0558
			3	,0808
GRlevél	Tamhane	0	1	,003219
			3	,002961
			7	,003515
		1	0	,003577
			3	,002818
			7	,003779
		3	0	,003958
			1	,003457
			7	,004037
		7	0	,005224
			1	,005131
			3	,004749

Homogeneous Subsets

MDHlevél

	Idő	N	Subset for alpha = 0.05 1	2	3
Duncan[a]	3	3	18,0267		
	1	3	20,4009	20,4009	
	0	3		21,5415	21,5415
	7	3			23,9871
	Sig.		,123	,432	,114

Means for groups in homogeneous subsets are displayed.

a. Uses Harmonic Mean Sample Size = 3,000.

GPXlevél

Idő		N	Subset for alpha = 0.05	
			1	2
Duncan[a]	7	3	,5299	
	3	3	,6393	,6393
	0	3	,6837	,6837
	1	3		,7886
	Sig.		,050	,056

Means for groups in homogeneous subsets are displayed.

a. Uses Harmonic Mean Sample Size = 3,000.

APXlevél

Idő		N	Subset for alpha = 0.05
			1
Duncan[a]	3	3	,1396
	7	3	,1412
	0	3	,1575
	1	3	,1626
	Sig.		,139

Means for groups in homogeneous subsets are displayed.

a. Uses Harmonic Mean Sample Size = 3,000.

GRlevél

Idő		N	Subset for alpha = 0.05
			1
Duncan[a]	0	3	,004596
	1	3	,004774
	3	3	,005094
	7	3	,005450
	Sig.		,323

Means for groups in homogeneous subsets are displayed.

a. Uses Harmonic Mean Sample Size = 3,000.

```
GET
  FILE='H:\Jócsák\01 Növényélettan\árpa vizsgálatok\PhD téma folytatása\SP
AD\SPAD-two-way-anova.sav'.
DATASET NAME DataSet4 WINDOW=FRONT.
DATASET ACTIVATE DataSet1.
DATASET CLOSE DataSet4.
```

```
DATASET CLOSE DataSet3.
DATASET ACTIVATE DataSet2.
USE ALL.
COMPUTE filter_$=(kadmium=10).
VARIABLE LABELS filter_$ 'kadmium=10 (FILTER)'.
VALUE LABELS filter_$ 0 'Not Selected' 1 'Selected'.
FORMATS filter_$ (f1.0).
FILTER BY filter_$.
EXECUTE.
```
