# Supplementary material for: Effect of cadmium stress on certain physiological parameters, antioxidative enzyme activities and biophoton emission of leaves in barley (Hordeum vulgare L.) seedlings
Source: PLoS One. 2020 Nov 3;15(11):e0240470. doi: 10.1371/journal.pone.0240470 (PMC7608874; doi:10.1371/journal.pone.0240470)
Supplement: S1 File — (ZIP) [file pone.0240470.s003.zip › stat result time-0 Cd SPAD leaf.pdf]

```
ONEWAY SPAD BY Idő
  /STATISTICS DESCRIPTIVES HOMOGENEITY
  /PLOT MEANS
  /MISSING ANALYSIS
  /POSTHOC=DUNCAN T2 ALPHA(0.05).
```

Oneway

[DataSet1] H:\Jócsák\01 Növényélettan\árpa vizsgálatok\PhD téma folytatása \SPAD\SPAD-two-way-anova.sav

Descriptives

SPAD

	N	Mean	Std. Deviation	Std. Error	95% Confidence Interval for Mean	
					Lower Bound	Upper Bound
0	100	28,0390	3,13310	,31331	27,4173	28,6607
1	100	29,5343	3,47909	,34791	28,8440	30,2246
3	100	27,1530	3,82109	,38211	26,3948	27,9112
7	100	29,8830	4,82291	,48229	28,9260	30,8400
Total	400	28,6523	4,00815	,20041	28,2583	29,0463

Descriptives

SPAD

	Minimum	Maximum
0	22,10	38,90
1	22,10	38,90
3	19,10	36,60
7	12,10	37,90
Total	12,10	38,90

Test of Homogeneity of Variances

SPAD

Levene Statistic	df1	df2	Sig.
5,511	3	396	,001

ANOVA

SPAD

	Sum of Squares	df	Mean Square	F	Sig.
Between Groups	491,658	3	163,886	10,966	,000
Within Groups	5918,369	396	14,945		
Total	6410,028	399			

Post Hoc Tests

Multiple Comparisons

Dependent Variable: SPAD

	(I) Idő	(J) Idő	Mean Difference (I-J)	Std. Error	Sig.	95% Confidence Interval	
						Lower Bound	Upper Bound
Tamhane	0	1	-1,49530*	,46819	,010	-2,7397	-,2509
		3	,88600	,49414	,372	-,4277	2,1997
		7	-1,84400*	,57512	,010	-3,3750	-,3130
	1	0	1,49530*	,46819	,010	,2509	2,7397
		3	2,38130*	,51677	,000	1,0078	3,7548
		7	-,34870	,59468	,993	-1,9307	1,2333
	3	0	-,88600	,49414	,372	-2,1997	,4277
		1	-2,38130*	,51677	,000	-3,7548	-1,0078
		7	-2,73000*	,61531	,000	-4,3661	-1,0939
	7	0	1,84400*	,57512	,010	,3130	3,3750
		1	,34870	,59468	,993	-1,2333	1,9307
		3	2,73000*	,61531	,000	1,0939	4,3661

*. The mean difference is significant at the 0.05 level.

Homogeneous Subsets

SPAD

	Idő	N	Subset for alpha = 0.05	
			1	2
Duncan[a]	3	100	27,1530	
	0	100	28,0390	
	1	100		29,5343
	7	100		29,8830
	Sig.		,106	,524

Means for groups in homogeneous subsets are displayed.

a. Uses Harmonic Mean Sample Size = 100,000.

Means Plots

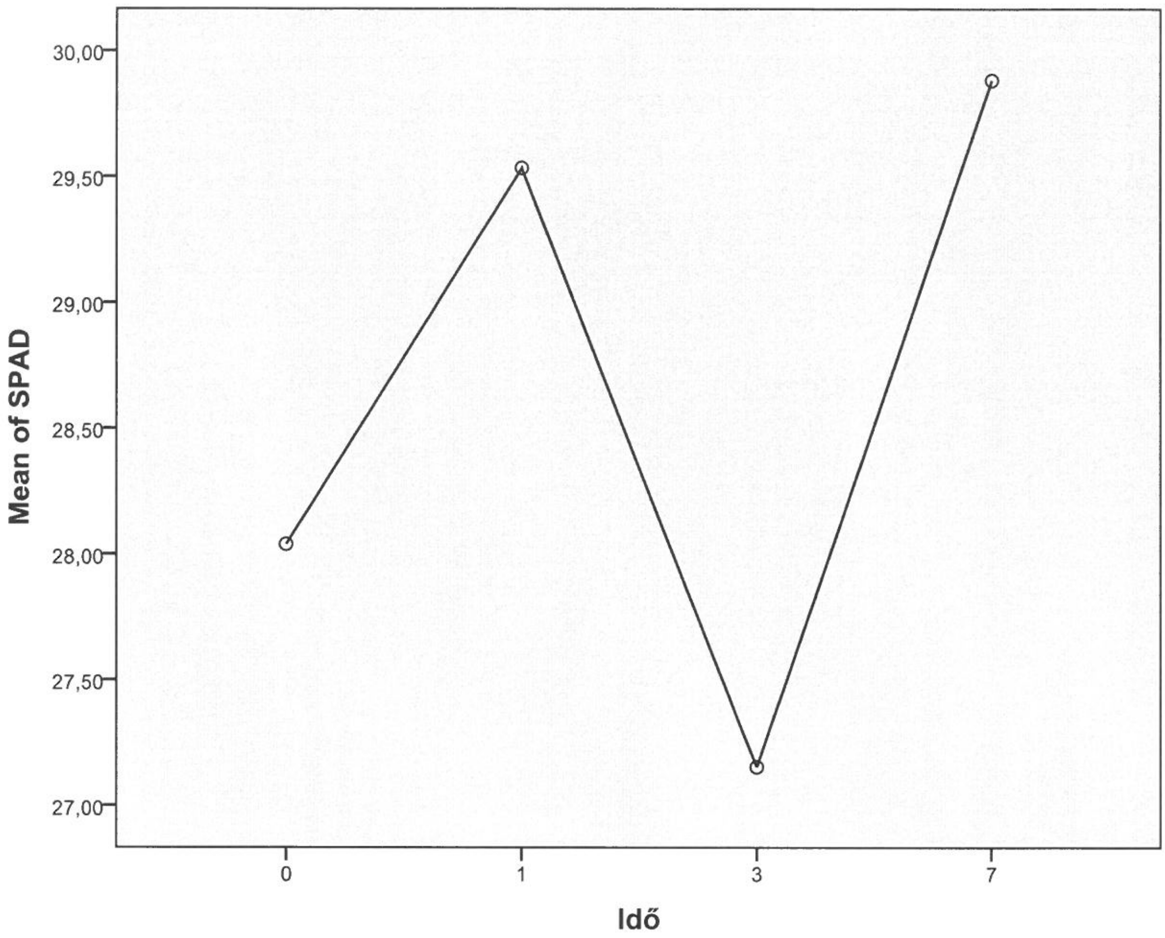
30,00
29,50
29,00
28,50
28,00
27,50
27,00
Mean of SPAD
0
1
3
7
Idő
