# Supplementary material for: Effect of cadmium stress on certain physiological parameters, antioxidative enzyme activities and biophoton emission of leaves in barley (Hordeum vulgare L.) seedlings
Source: PLoS One. 2020 Nov 3;15(11):e0240470. doi: 10.1371/journal.pone.0240470 (PMC7608874; doi:10.1371/journal.pone.0240470)
Supplement: S1 File — (ZIP) [file pone.0240470.s003.zip › stat result time-10 Cd AA leaf.pdf]

```
ONEWAY AA1 BY Idő
  /STATISTICS DESCRIPTIVES HOMOGENEITY
  /MISSING ANALYSIS
  /POSTHOC=DUNCAN T2 ALPHA(0.05).
```

Oneway

[DataSet2] H:\Jócsák\01 Növényélettan\árpa vizsgálatok\PhD téma folytatása \Visi É árpa c vit meghatározás\aszkorbinsav mg-g fr tömeg.sav

Descriptives

AA1

	N	Mean	Std. Deviation	Std. Error	95% Confidence Interval for Mean	
					Lower Bound	Upper Bound
1	2	,5904	,00057	,00040	,5853	,5955
3	2	,6722	,05636	,03985	,1658	1,1785
7	2	,9365	,31742	,22445	-1,9155	3,7884
Total	6	,7330	,21670	,08847	,5056	,9604

Descriptives

AA1

	Minimum	Maximum
1	,59	,59
3	,63	,71
7	,71	1,16
Total	,59	1,16

Test of Homogeneity of Variances

AA1

Levene Statistic	df1	df2	Sig.
.	2	.	.

ANOVA

AA1

	Sum of Squares	df	Mean Square	F	Sig.
Between Groups	,131	2	,065	1,889	,295
Within Groups	,104	3	,035		
Total	,235	5			

Post Hoc Tests

Multiple Comparisons

Dependent Variable: AA1

	(I) Idő	(J) Idő	Mean Difference (I-J)	Std. Error	Sig.	95% Confidence Interval	
						Lower Bound	Upper Bound
Tamhane	1	3	-,08175	,03985	,640	-1,5769	1,4134
		7	-,34605	,22445	,746	-8,7727	8,0806
	3	1	,08175	,03985	,640	-1,4134	1,5769
		7	-,26430	,22796	,828	-7,2555	6,7269
	7	1	,34605	,22445	,746	-8,0806	8,7727
		3	,26430	,22796	,828	-6,7269	7,2555

Homogeneous Subsets

AA1

	Idő	N	Subset for alpha = 0.05
			1
Duncan[a]	1	2	,5904
	3	2	,6722
	7	2	,9365
	Sig.		,160

Means for groups in homogeneous subsets are displayed.

a. Uses Harmonic Mean Sample Size = 2,000.
