# Supplementary material for: Effect of cadmium stress on certain physiological parameters, antioxidative enzyme activities and biophoton emission of leaves in barley (Hordeum vulgare L.) seedlings
Source: PLoS One. 2020 Nov 3;15(11):e0240470. doi: 10.1371/journal.pone.0240470 (PMC7608874; doi:10.1371/journal.pone.0240470)
Supplement: S1 File — (ZIP) [file pone.0240470.s003.zip › stat result time-10 Cd content leaf.pdf]

```
ONEWAY Kadmiumtartlev BY Idő
  /STATISTICS DESCRIPTIVES HOMOGENEITY
  /PLOT MEANS
  /MISSING ANALYSIS
  /POSTHOC=DUNCAN T2 ALPHA(0.05).
```

# Oneway

[DataSet1] H:\Jócsák\01 Növényélettan\árpa vizsgálatok\PhD téma folytatása \MGHgyökér_1.sav

**Descriptives**

Kadmiumtartlev

| | N | Mean | Std. Deviation | Std. Error | 95% Confidence Interval for Mean | |
|---|---|---|---|---|---|---|
| | | | | | Lower Bound | Upper Bound |
| 0 | 3 | ,5367 | ,03055 | ,01764 | ,4608 | ,6126 |
| 1 | 3 | 10,4633 | ,76009 | ,43884 | 8,5752 | 12,3515 |
| 3 | 3 | 7,8367 | ,68017 | ,39270 | 6,1470 | 9,5263 |
| 7 | 3 | 8,7400 | ,82177 | ,47445 | 6,6986 | 10,7814 |
| Total | 12 | 6,8942 | 3,99761 | 1,15401 | 4,3542 | 9,4341 |

**Descriptives**

Kadmiumtartlev

| | Minimum | Maximum |
|---|---|---|
| 0 | ,51 | ,57 |
| 1 | 9,71 | 11,23 |
| 3 | 7,21 | 8,56 |
| 7 | 8,01 | 9,63 |
| Total | ,51 | 11,23 |

**Test of Homogeneity of Variances**

Kadmiumtartlev

| Levene Statistic | df1 | df2 | Sig. |
|---|---|---|---|
| 1,782 | 3 | 8 | ,228 |

**ANOVA**

Kadmiumtartlev

| | Sum of Squares | df | Mean Square | F | Sig. |
|---|---|---|---|---|---|
| Between Groups | 172,356 | 3 | 57,452 | 133,874 | ,000 |
| Within Groups | 3,433 | 8 | ,429 | | |
| Total | 175,790 | 11 | | | |

## Post Hoc Tests

**Multiple Comparisons**

Dependent Variable: Kadmiumtartlev

| | (I) Idő | (J) Idő | Mean Difference (I-J) | Std. Error | Sig. | 95% Confidence Interval | |
|---|---|---|---|---|---|---|---|
| | | | | | | Lower Bound | Upper Bound |
| Tamhane | 0 | 1 | -9,92667* | ,43919 | ,011 | -14,6297 | -5,2236 |
| | | 3 | -7,30000* | ,39309 | ,017 | -11,5035 | -3,0965 |
| | | 7 | -8,20333* | ,47477 | ,020 | -13,2916 | -3,1151 |
| | 1 | 0 | 9,92667* | ,43919 | ,011 | 5,2236 | 14,6297 |
| | | 3 | 2,62667 | ,58889 | ,067 | -,2378 | 5,4911 |
| | | 7 | 1,72333 | ,64628 | ,294 | -1,4064 | 4,8530 |
| | 3 | 0 | 7,30000* | ,39309 | ,017 | 3,0965 | 11,5035 |
| | | 1 | -2,62667 | ,58889 | ,067 | -5,4911 | ,2378 |
| | | 7 | -,90333 | ,61588 | ,773 | -3,9483 | 2,1417 |
| | 7 | 0 | 8,20333* | ,47477 | ,020 | 3,1151 | 13,2916 |
| | | 1 | -1,72333 | ,64628 | ,294 | -4,8530 | 1,4064 |
| | | 3 | ,90333 | ,61588 | ,773 | -2,1417 | 3,9483 |

*. The mean difference is significant at the 0.05 level.

## Homogeneous Subsets

**Kadmiumtartlev**

| | Idő | N | Subset for alpha = 0.05 | | |
|---|---|---|---|---|---|
| | | | 1 | 2 | 3 |
| Duncan[a] | 0 | 3 | ,5367 | | |
| | 3 | 3 | | 7,8367 | |
| | 7 | 3 | | 8,7400 | |
| | 1 | 3 | | | 10,4633 |
| | Sig. | | 1,000 | ,130 | 1,000 |

Means for groups in homogeneous subsets are displayed.

a. Uses Harmonic Mean Sample Size = 3,000.

## Means Plots

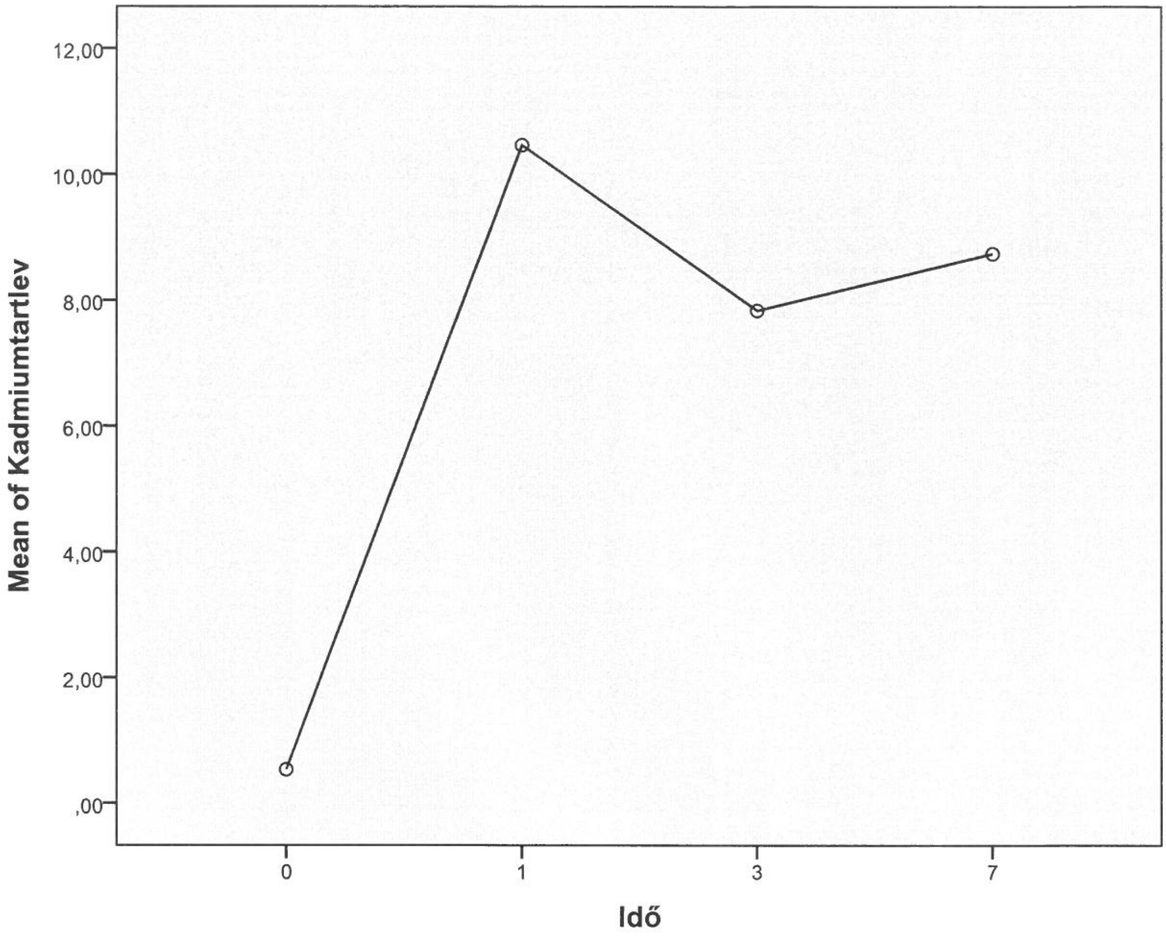

12,00
10,00
8,00
6,00
4,00
2,00
,00
Mean of Kadmiumtartlev
0
1
3
7
Idő
