# Supplementary material for: Effect of cadmium stress on certain physiological parameters, antioxidative enzyme activities and biophoton emission of leaves in barley (Hordeum vulgare L.) seedlings
Source: PLoS One. 2020 Nov 3;15(11):e0240470. doi: 10.1371/journal.pone.0240470 (PMC7608874; doi:10.1371/journal.pone.0240470)
Supplement: S1 File — (ZIP) [file pone.0240470.s003.zip › stat result time-10 Cd MDH-enzyme leaf.pdf]

```
ONEWAY MDHlevél GPXlevél APXlevél GRlevél BY Idő
  /STATISTICS DESCRIPTIVES HOMOGENEITY
  /MISSING ANALYSIS
  /POSTHOC=DUNCAN T2 ALPHA(0.05).
```

Oneway

[DataSet2] H:\Jócsák\01 Növényélettan\árpa vizsgálatok\PhD téma folytatása \MGHgyökér_1.sav

Descriptives

		N	Mean	Std. Deviation	Std. Error	95% Confidence ...
						Lower Bound
MDHlevél	0	3	21,5415	1,68115	,97061	17,3652
	1	3	19,1132	6,78541	3,91756	2,2573
	3	3	18,8989	,45394	,26208	17,7713
	7	3	31,4740	1,33240	,76926	28,1641
	Total	12	22,7569	6,16865	1,78073	18,8375
GPXlevél	0	3	,6837	,04569	,02638	,5702
	1	3	1,2124	,03181	,01837	1,1334
	3	3	,9043	,32158	,18566	,1054
	7	3	1,2487	,11928	,06887	,9524
	Total	12	1,0123	,28417	,08203	,8317
APXlevél	0	3	,1575	,01006	,00581	,1325
	1	3	,1488	,01886	,01089	,1019
	3	3	,1856	,00943	,00544	,1622
	7	3	,2130	,02590	,01496	,1486
	Total	12	,1762	,03024	,00873	,1570
GRlevél	0	3	,004596	,0009166	,0005292	,002319
	1	3	,005399	,0022163	,0012796	-,000107
	3	3	,005930	,0009826	,0005673	,003489
	7	3	,007382	,0022275	,0012861	,001849
	Total	12	,005827	,0018027	,0005204	,004681

Descriptives

		95% Confidence ...		
		Upper Bound	Minimum	Maximum
MDHlevél	0	25,7177	19,60	22,59
	1	35,9691	11,29	23,46
	3	20,0266	18,54	19,41
	7	34,7838	30,31	32,93
	Total	26,6763	11,29	32,93
GPXlevél	0	,7972	,64	,73
	1	1,2914	1,19	1,25
	3	1,7031	,56	1,19
	7	1,5450	1,13	1,37
	Total	1,1928	,56	1,37
APXlevél	0	,1825	,15	,17
	1	,1956	,13	,17
	3	,2091	,18	,20
	7	,2773	,19	,24
	Total	,1954	,13	,24
GRlevél	0	,006873	,0037	,0055
	1	,010904	,0038	,0079
	3	,008371	,0050	,0070
	7	,012916	,0057	,0099
	Total	,006972	,0037	,0099

Test of Homogeneity of Variances

	Levene Statistic	df1	df2	Sig.
MDHlevél	9,752	3	8	,005
GPXlevél	3,920	3	8	,054
APXlevél	,961	3	8	,457
GRlevél	2,207	3	8	,165

ANOVA

		Sum of Squares	df	Mean Square	F	Sig.
MDHlevél	Between Groups	316,875	3	105,625	8,309	,008
	Within Groups	101,699	8	12,712		
	Total	418,574	11			
GPXlevél	Between Groups	,647	3	,216	7,142	,012
	Within Groups	,241	8	,030		
	Total	,888	11			
APXlevél	Between Groups	,008	3	,003	8,358	,008
	Within Groups	,002	8	,000		
	Total	,010	11			
GRlevél	Between Groups	,000	3	,000	1,414	,308
	Within Groups	,000	8	,000		
	Total	,000	11			

Post Hoc Tests

Multiple Comparisons

Dependent Variable		(I) Idő	(J) Idő	Mean Difference (I-J)	Std. Error	Sig.	95% ... Lower Bound
MDHlevél	Tamhane	0	1	2,42823	4,03601	,996	-33,5652
			3	2,64253	1,00537	,482	-6,0585
			7	-9,93252*	1,23849	,010	-16,1326
		1	0	-2,42823	4,03601	,996	-38,4216
			3	,21430	3,92632	1,000	-41,4152
			7	-12,36075	3,99237	,403	-50,3238
		3	0	-2,64253	1,00537	,482	-11,3435
			1	-,21430	3,92632	1,000	-41,8438
			7	-12,57505*	,81268	,010	-18,9400
		7	0	9,93252*	1,23849	,010	3,7325
			1	12,36075	3,99237	,403	-25,6023
			3	12,57505*	,81268	,010	6,2101
GPXlevél	Tamhane	0	1	-,52872*	,03215	,001	-,6979
			3	-,22060	,18753	,929	-2,1076
			7	-,56503*	,07375	,046	-1,1087
		1	0	,52872*	,03215	,001	,3595
			3	,30811	,18657	,804	-1,6346
			7	-,03631	,07127	,998	-,6560
		3	0	,22060	,18753	,929	-1,6664
			1	-,30811	,18657	,804	-2,2508
			7	-,34443	,19802	,731	-1,8297
		7	0	,56503*	,07375	,046	,0213
			1	,03631	,07127	,998	-,5833
			3	,34443	,19802	,731	-1,1409

Multiple Comparisons

Dependent Variable		(I) Idő	(J) Idő	95% ... Upper Bound
MDHlevél	Tamhane	0	1	38,4216
			3	11,3435
			7	-3,7325
		1	0	33,5652
			3	41,8438
			7	25,6023
		3	0	6,0585
			1	41,4152
			7	-6,2101
		7	0	16,1326
			1	50,3238
			3	18,9400
GPXlevél	Tamhane	0	1	-,3595
			3	1,6664
			7	-,0213
		1	0	,6979
			3	2,2508
			7	,5833
		3	0	2,1076
			1	1,6346
			7	1,1409
		7	0	1,1087
			1	,6560
			3	1,8297

Multiple Comparisons

Dependent Variable		(I) Idő	(J) Idő	Mean Difference (I-J)	Std. Error	Sig.	95% ...
							Lower Bound
APXlevél	Tamhane	0	1	,00874	,01234	,989	-,0662
			3	-,02811	,00796	,138	-,0666
			7	-,05546	,01604	,269	-,1728
		1	0	-,00874	,01234	,989	-,0837
			3	-,03685	,01217	,301	-,1137
			7	-,06420	,01850	,165	-,1597
		3	0	,02811	,00796	,138	-,0104
			1	,03685	,01217	,301	-,0400
			7	-,02735	,01592	,740	-,1479
		7	0	,05546	,01604	,269	-,0619
			1	,06420	,01850	,165	-,0313
			3	,02735	,01592	,740	-,0932
GRlevél	Tamhane	0	1	-,0008031	,0013847	,996	-,010585
			3	-,0013342	,0007758	,651	-,005088
			7	-,0027866	,0013907	,624	-,012640
		1	0	,0008031	,0013847	,996	-,008979
			3	-,0005312	,0013997	1,000	-,010029
			7	-,0019835	,0018142	,914	-,010731
		3	0	,0013342	,0007758	,651	-,002419
			1	,0005312	,0013997	1,000	-,008966
			7	-,0014524	,0014056	,945	-,011018
		7	0	,0027866	,0013907	,624	-,007066
			1	,0019835	,0018142	,914	-,006764
			3	,0014524	,0014056	,945	-,008114

Multiple Comparisons

Dependent Variable		(I) Idő	(J) Idő	95% ... Upper Bound
APXlevél	Tamhane	0	1	,0837
			3	,0104
			7	,0619
		1	0	,0662
			3	,0400
			7	,0313
		3	0	,0666
			1	,1137
			7	,0932
		7	0	,1728
			1	,1597
			3	,1479
GRlevél	Tamhane	0	1	,008979
			3	,002419
			7	,007066
		1	0	,010585
			3	,008966
			7	,006764
		3	0	,005088
			1	,010029
			7	,008114
		7	0	,012640
			1	,010731
			3	,011018

*. The mean difference is significant at the 0.05 level.

Homogeneous Subsets

MDHlevél

	Idő	N	Subset for alpha = 0.05 1	2
Duncan[a]	3	3	18,8989	
	1	3	19,1132	
	0	3	21,5415	
	7	3		31,4740
	Sig.		,409	1,000

Means for groups in homogeneous subsets are displayed.

a. Uses Harmonic Mean Sample Size = 3,000.

GPXlevél

Idő		N	Subset for alpha = 0.05		
			1	2	3
Duncan[a]	0	3	,6837		
	3	3	,9043	,9043	
	1	3		1,2124	1,2124
	7	3			1,2487
	Sig.		,159	,062	,804

Means for groups in homogeneous subsets are displayed.

a. Uses Harmonic Mean Sample Size = 3,000.

APXlevél

Idő		N	Subset for alpha = 0.05		
			1	2	3
Duncan[a]	1	3	,1488		
	0	3	,1575	,1575	
	3	3		,1856	,1856
	7	3			,2130
	Sig.		,556	,084	,091

Means for groups in homogeneous subsets are displayed.

a. Uses Harmonic Mean Sample Size = 3,000.

GRlevél

Idő		N	Subset for alpha = 0.05
			1
Duncan[a]	0	3	,004596
	1	3	,005399
	3	3	,005930
	7	3	,007382
	Sig.		,098

Means for groups in homogeneous subsets are displayed.

a. Uses Harmonic Mean Sample Size = 3,000.
