# Supplementary material for: Effect of cadmium stress on certain physiological parameters, antioxidative enzyme activities and biophoton emission of leaves in barley (Hordeum vulgare L.) seedlings
Source: PLoS One. 2020 Nov 3;15(11):e0240470. doi: 10.1371/journal.pone.0240470 (PMC7608874; doi:10.1371/journal.pone.0240470)
Supplement: S1 File — (ZIP) [file pone.0240470.s003.zip › stat result time-100 Cd content leaf.pdf]

```
ONEWAY Kadmiumtartlev BY Idő
  /STATISTICS DESCRIPTIVES HOMOGENEITY
  /PLOT MEANS
  /MISSING ANALYSIS
  /POSTHOC=DUNCAN T2 ALPHA(0.05).
```

Oneway

[DataSet1] H:\Jócsák\01 Növényélettan\árpa vizsgálatok\PhD téma folytatása \MGHgyökér_1.sav

Descriptives

Kadmiumtartlev

	N	Mean	Std. Deviation	Std. Error	95% Confidence Interval for Mean	
					Lower Bound	Upper Bound
0	3	,5367	,03055	,01764	,4608	,6126
1	3	27,1567	2,44876	1,41379	21,0736	33,2397
3	3	61,0033	3,51654	2,03027	52,2678	69,7389
7	3	80,2033	7,93224	4,57968	60,4986	99,9081
Total	12	42,2250	32,25244	9,31048	21,7328	62,7172

Descriptives

Kadmiumtartlev

	Minimum	Maximum
0	,51	,57
1	24,79	29,68
3	56,98	63,49
7	72,96	88,68
Total	,51	88,68

Test of Homogeneity of Variances

Kadmiumtartlev

Levene Statistic	df1	df2	Sig.
3,657	3	8	,063

ANOVA

Kadmiumtartlev

	Sum of Squares	df	Mean Square	F	Sig.
Between Groups	11279,854	3	3759,951	185,028	,000
Within Groups	162,568	8	20,321		
Total	11442,422	11			

Post Hoc Tests

Multiple Comparisons

Dependent Variable: Kadmiumtartlev

	(I) Idő	(J) Idő	Mean Difference (I-J)	Std. Error	Sig.	95% Confidence Interval	
						Lower Bound	Upper Bound
Tamhane	0	1	-26,62000*	1,41390	,017	-41,8384	-11,4016
		3	-60,46667*	2,03035	,007	-82,3263	-38,6071
		7	-79,66667*	4,57971	,020	-128,9843	-30,3490
	1	0	26,62000*	1,41390	,017	11,4016	41,8384
		3	-33,84667*	2,47403	,002	-46,8653	-20,8281
		7	-53,04667*	4,79294	,025	-92,3411	-13,7522
	3	0	60,46667*	2,03035	,007	38,6071	82,3263
		1	33,84667*	2,47403	,002	20,8281	46,8653
		7	-19,20000	5,00954	,199	-53,1928	14,7928
	7	0	79,66667*	4,57971	,020	30,3490	128,9843
		1	53,04667*	4,79294	,025	13,7522	92,3411
		3	19,20000	5,00954	,199	-14,7928	53,1928

*. The mean difference is significant at the 0.05 level.

Homogeneous Subsets

Kadmiumtartlev

	Idő	N	Subset for alpha = 0.05			
			1	2	3	4
Duncan[a]	0	3	,5367			
	1	3		27,1567		
	3	3			61,0033	
	7	3				80,2033
	Sig.		1,000	1,000	1,000	1,000

Means for groups in homogeneous subsets are displayed.

a. Uses Harmonic Mean Sample Size = 3,000.

Means Plots

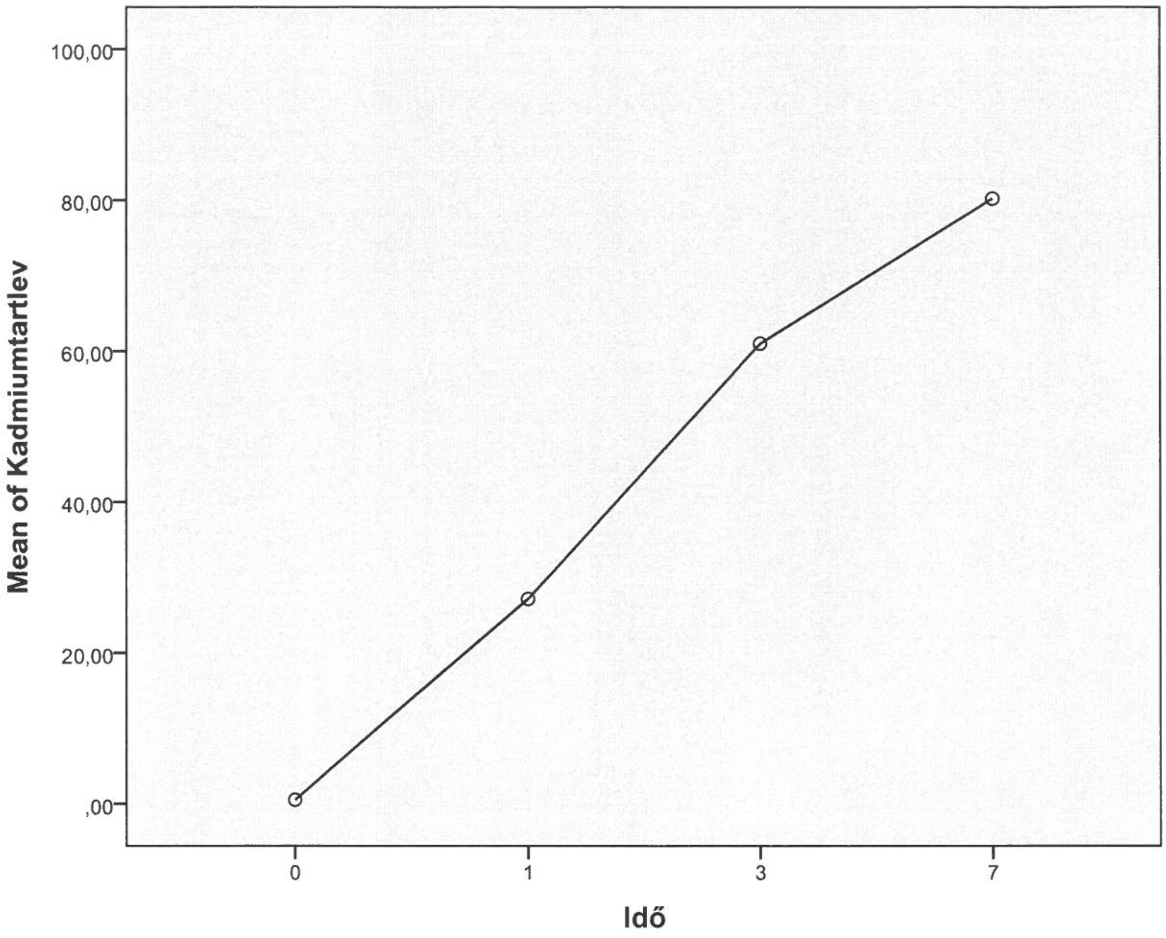
100,00
80,00
60,00
40,00
20,00
,00
Mean of Kadmiumtartlev
0
1
3
7
Idő
