# Supplementary material for: Effect of cadmium stress on certain physiological parameters, antioxidative enzyme activities and biophoton emission of leaves in barley (Hordeum vulgare L.) seedlings
Source: PLoS One. 2020 Nov 3;15(11):e0240470. doi: 10.1371/journal.pone.0240470 (PMC7608874; doi:10.1371/journal.pone.0240470)
Supplement: S1 File — (ZIP) [file pone.0240470.s003.zip › stat result time-100 Cd MDA-enzyme leaf.pdf]

```
ONEWAY MDHlevél GPXlevél APXlevél GRlevél BY Idő
  /STATISTICS DESCRIPTIVES HOMOGENEITY
  /MISSING ANALYSIS
  /POSTHOC=DUNCAN T2 ALPHA(0.05).
```

Oneway

[DataSet2] H:\Jócsák\01 Növényélettan\árpa vizsgálatok\PhD téma folytatása \MGHgyökér_1.sav

Descriptives

		N	Mean	Std. Deviation	Std. Error	95% Confidence ... Lower Bound
MDHlevél	0	3	21,5415	1,68115	,97061	17,3652
	1	3	20,5203	1,02439	,59143	17,9756
	3	3	25,0170	,72218	,41695	23,2230
	7	3	54,7342	,75540	,43613	52,8577
	Total	12	30,4532	14,77571	4,26538	21,0652
GPXlevél	0	3	,6837	,04569	,02638	,5702
	1	3	,8052	,05518	,03186	,6682
	3	3	2,6994	,14900	,08603	2,3292
	7	3	3,2499	,14619	,08440	2,8868
	Total	12	1,8596	1,18688	,34262	1,1054
APXlevél	0	3	,1575	,01006	,00581	,1325
	1	3	,2175	,01962	,01133	,1688
	3	3	,2569	,02576	,01487	,1929
	7	3	,3261	,02191	,01265	,2717
	Total	12	,2395	,06626	,01913	,1974
GRlevél	0	3	,004596	,0009166	,0005292	,002319
	1	3	,005361	,0019480	,0011247	,000522
	3	3	,005779	,0028076	,0016210	-,001196
	7	3	,007807	,0045571	,0026310	-,003514
	Total	12	,005886	,0027550	,0007953	,004135

Descriptives

		95% Confidence ...		
		Upper Bound	Minimum	Maximum
MDHlevél	0	25,7177	19,60	22,59
	1	23,0650	19,73	21,68
	3	26,8110	24,18	25,43
	7	56,6107	54,30	55,61
	Total	39,8413	19,60	55,61
GPXlevél	0	,7972	,64	,73
	1	,9423	,75	,86
	3	3,0695	2,58	2,87
	7	3,6131	3,09	3,38
	Total	2,6137	,64	3,38
APXlevél	0	,1825	,15	,17
	1	,2663	,20	,24
	3	,3209	,23	,28
	7	,3806	,30	,35
	Total	,2816	,15	,35
GRlevél	0	,006873	,0037	,0055
	1	,010200	,0038	,0075
	3	,012754	,0031	,0087
	7	,019127	,0043	,0130
	Total	,007636	,0031	,0130

Test of Homogeneity of Variances

	Levene Statistic	df1	df2	Sig.
MDHlevél	2,344	3	8	,149
GPXlevél	2,122	3	8	,176
APXlevél	,719	3	8	,568
GRlevél	2,769	3	8	,111

ANOVA

		Sum of Squares	df	Mean Square	F	Sig.
MDHlevél	Between Groups	2391,602	3	797,201	641,894	,000
	Within Groups	9,936	8	1,242		
	Total	2401,538	11			
GPXlevél	Between Groups	15,398	3	5,133	421,528	,000
	Within Groups	,097	8	,012		
	Total	15,496	11			
APXlevél	Between Groups	,045	3	,015	36,850	,000
	Within Groups	,003	8	,000		
	Total	,048	11			
GRlevél	Between Groups	,000	3	,000	,678	,590
	Within Groups	,000	8	,000		
	Total	,000	11			

Post Hoc Tests

Multiple Comparisons

Dependent Variable		(I) Idő	(J) Idő	Mean Difference (I-J)	Std. Error	Sig.	95% ...
							Lower Bound
MDHlevél	Tamhane	0	1	1,02114	1,13661	,966	-5,3751
			3	-3,47551	1,05638	,280	-10,7771
			7	-33,19274*	1,06409	,001	-40,3561
		1	0	-1,02114	1,13661	,966	-7,4174
			3	-4,49664*	,72363	,029	-8,2836
			7	-34,21387*	,73485	,000	-37,9881
		3	0	3,47551	1,05638	,280	-3,8261
			1	4,49664*	,72363	,029	,7097
			7	-29,71723*	,60337	,000	-32,6307
		7	0	33,19274*	1,06409	,001	26,0294
			1	34,21387*	,73485	,000	30,4397
			3	29,71723*	,60337	,000	26,8038
GPXlevél	Tamhane	0	1	-,12156	,04136	,238	-,3260
			3	-2,01570*	,08998	,005	-2,7555
			7	-2,56626*	,08843	,003	-3,2873
		1	0	,12156	,04136	,238	-,0829
			3	-1,89414*	,09174	,004	-2,5828
			7	-2,44470*	,09021	,002	-3,1151
		3	0	2,01570*	,08998	,005	1,2758
			1	1,89414*	,09174	,004	1,2055
			7	-,55056	,12052	,060	-1,1318
		7	0	2,56626*	,08843	,003	1,8453
			1	2,44470*	,09021	,002	1,7743
			3	,55056	,12052	,060	-,0307

Multiple Comparisons

Dependent Variable		(I) Idő	(J) Idő	95% ... Upper Bound
MDHlevél	Tamhane	0	1	7,4174
			3	3,8261
			7	-26,0294
		1	0	5,3751
			3	-,7097
			7	-30,4397
		3	0	10,7771
			1	8,2836
			7	-26,8038
		7	0	40,3561
			1	37,9881
			3	32,6307
GPXlevél	Tamhane	0	1	,0829
			3	-1,2758
			7	-1,8453
		1	0	,3260
			3	-1,2055
			7	-1,7743
		3	0	2,7555
			1	2,5828
			7	,0307
		7	0	3,2873
			1	3,1151
			3	1,1318

Multiple Comparisons

Dependent Variable		(I) Idő	(J) Idő	Mean Difference (I-J)	Std. Error	Sig.	95% ...
							Lower Bound
APXlevél	Tamhane	0	1	-,06004	,01273	,105	-,1392
			3	-,09940	,01597	,073	-,2158
			7	-,16863*	,01392	,010	-,2611
		1	0	,06004	,01273	,105	-,0191
			3	-,03936	,01869	,496	-,1342
			7	-,10859*	,01698	,019	-,1912
		3	0	,09940	,01597	,073	-,0170
			1	,03936	,01869	,496	-,0555
			7	-,06923	,01952	,140	-,1651
		7	0	,16863*	,01392	,010	,0761
			1	,10859*	,01698	,019	,0260
			3	,06923	,01952	,140	-,0267
GRlevél	Tamhane	0	1	-,0007653	,0012430	,995	-,008906
			3	-,0011832	,0017052	,992	-,014813
			7	-,0032108	,0026837	,922	-,028588
		1	0	,0007653	,0012430	,995	-,007375
			3	-,0004180	,0019729	1,000	-,010817
			7	-,0024456	,0028613	,976	-,022279
		3	0	,0011832	,0017052	,992	-,012447
			1	,0004180	,0019729	1,000	-,009981
			7	-,0020276	,0030903	,992	-,019314
		7	0	,0032108	,0026837	,922	-,022166
			1	,0024456	,0028613	,976	-,017388
			3	,0020276	,0030903	,992	-,015259

Multiple Comparisons

Dependent Variable		(I) Idő	(J) Idő	95% ... Upper Bound
APXlevél	Tamhane	0	1	,0191
			3	,0170
			7	-,0761
		1	0	,1392
			3	,0555
			7	-,0260
		3	0	,2158
			1	,1342
			7	,0267
		7	0	,2611
			1	,1912
			3	,1651
GRlevél	Tamhane	0	1	,007375
			3	,012447
			7	,022166
		1	0	,008906
			3	,009981
			7	,017388
		3	0	,014813
			1	,010817
			7	,015259
		7	0	,028588
			1	,022279
			3	,019314

*. The mean difference is significant at the 0.05 level.

Homogeneous Subsets

MDHlevél

	Idő	N	Subset for alpha = 0.05 1	2	3
Duncan[a]	1	3	20,5203		
	0	3	21,5415		
	3	3		25,0170	
	7	3			54,7342
	Sig.		,294	1,000	1,000

Means for groups in homogeneous subsets are displayed.

a. Uses Harmonic Mean Sample Size = 3,000.

GPXlevél

	Idő	N	Subset for alpha = 0.05		
			1	2	3
Duncan[a]	0	3	,6837		
	1	3	,8052		
	3	3		2,6994	
	7	3			3,2499
	Sig.		,214	1,000	1,000

Means for groups in homogeneous subsets are displayed.

a. Uses Harmonic Mean Sample Size = 3,000.

APXlevél

	Idő	N	Subset for alpha = 0.05			
			1	2	3	4
Duncan[a]	0	3	,1575			
	1	3		,2175		
	3	3			,2569	
	7	3				,3261
	Sig.		1,000	1,000	1,000	1,000

Means for groups in homogeneous subsets are displayed.

a. Uses Harmonic Mean Sample Size = 3,000.

GRlevél

	Idő	N	Subset for alpha = 0.05
			1
Duncan[a]	0	3	,004596
	1	3	,005361
	3	3	,005779
	7	3	,007807
	Sig.		,235

Means for groups in homogeneous subsets are displayed.

a. Uses Harmonic Mean Sample Size = 3,000.
