# Supplementary material for: Effect of cadmium stress on certain physiological parameters, antioxidative enzyme activities and biophoton emission of leaves in barley (Hordeum vulgare L.) seedlings
Source: PLoS One. 2020 Nov 3;15(11):e0240470. doi: 10.1371/journal.pone.0240470 (PMC7608874; doi:10.1371/journal.pone.0240470)
Supplement: S1 File — (ZIP) [file pone.0240470.s003.zip › stat result time-100 Cd SPAD leaf.pdf]

```
ONEWAY SPAD BY Idő
  /STATISTICS DESCRIPTIVES HOMOGENEITY
  /PLOT MEANS
  /MISSING ANALYSIS
  /POSTHOC=DUNCAN T2 ALPHA(0.05).
```

Oneway

[DataSet1] H:\Jócsák\01 Növényélettan\árpa vizsgálatok\PhD téma folytatása \SPAD\SPAD-two-way-anova.sav

Descriptives

SPAD

	N	Mean	Std. Deviation	Std. Error	95% Confidence Interval for Mean	
					Lower Bound	Upper Bound
0	100	28,0390	3,13310	,31331	27,4173	28,6607
1	100	27,5410	2,61758	,26176	27,0216	28,0604
3	100	20,9040	4,67635	,46764	19,9761	21,8319
7	100	16,2290	7,23953	,72395	14,7925	17,6655
Total	400	23,1782	6,83061	,34153	22,5068	23,8497

Descriptives

SPAD

	Minimum	Maximum
0	22,10	38,90
1	22,10	38,90
3	11,00	30,20
7	2,00	29,90
Total	2,00	38,90

Test of Homogeneity of Variances

SPAD

Levene Statistic	df1	df2	Sig.
41,278	3	396	,000

ANOVA

SPAD

	Sum of Squares	df	Mean Square	F	Sig.
Between Groups	9612,477	3	3204,159	140,924	,000
Within Groups	9003,764	396	22,737		
Total	18616,241	399			

Post Hoc Tests

Multiple Comparisons

Dependent Variable: SPAD

	(I) Idő	(J) Idő	Mean Difference (I-J)	Std. Error	Sig.	95% Confidence Interval Lower Bound	95% Confidence Interval Upper Bound
Tamhane	0	1	,49800	,40827	,782	-,5874	1,5834
		3	7,13500*	,56289	,000	5,6369	8,6331
		7	11,81000*	,78884	,000	9,7036	13,9164
	1	0	-,49800	,40827	,782	-1,5834	,5874
		3	6,63700*	,53591	,000	5,2088	8,0652
		7	11,31200*	,76982	,000	9,2538	13,3702
	3	0	-7,13500*	,56289	,000	-8,6331	-5,6369
		1	-6,63700*	,53591	,000	-8,0652	-5,2088
		7	4,67500*	,86185	,000	2,3806	6,9694
	7	0	-11,81000*	,78884	,000	-13,9164	-9,7036
		1	-11,31200*	,76982	,000	-13,3702	-9,2538
		3	-4,67500*	,86185	,000	-6,9694	-2,3806

*. The mean difference is significant at the 0.05 level.

Homogeneous Subsets

SPAD

	Idő	N	Subset for alpha = 0.05: 1	2	3
Duncan[a]	7	100	16,2290		
	3	100		20,9040	
	1	100			27,5410
	0	100			28,0390
	Sig.		1,000	1,000	,461

Means for groups in homogeneous subsets are displayed.

a. Uses Harmonic Mean Sample Size = 100,000.

Means Plots

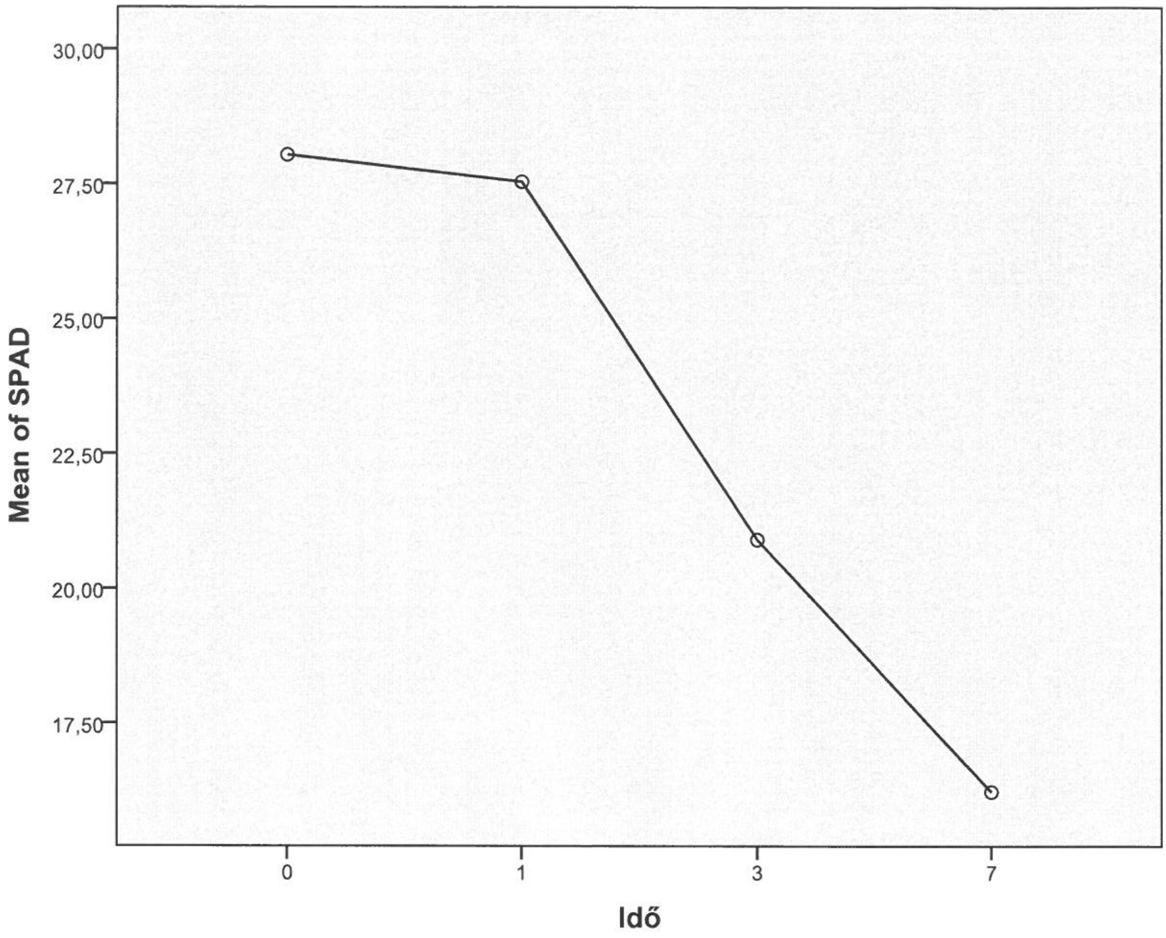
Mean of SPAD
30,00
27,50
25,00
22,50
20,00
17,50
0
1
3
7
Idő
