# Supplementary material for: Effect of cadmium stress on certain physiological parameters, antioxidative enzyme activities and biophoton emission of leaves in barley (Hordeum vulgare L.) seedlings
Source: PLoS One. 2020 Nov 3;15(11):e0240470. doi: 10.1371/journal.pone.0240470 (PMC7608874; doi:10.1371/journal.pone.0240470)
Supplement: S1 File — (ZIP) [file pone.0240470.s003.zip › stat result time-300 Cd AA leaf.pdf]

```
ONEWAY AA1 BY Idő
  /STATISTICS DESCRIPTIVES HOMOGENEITY
  /MISSING ANALYSIS
  /POSTHOC=DUNCAN T2 ALPHA(0.05).
```

Oneway

[DataSet2] H:\Jócsák\01 Növényélettan\árpa vizsgálatok\PhD téma folytatása \Visi É árpa c vit meghatározás\aszkorbinsav mg-g fr tömeg.sav

Descriptives

AA1

	N	Mean	Std. Deviation	Std. Error	95% Confidence Interval for Mean	
					Lower Bound	Upper Bound
1	2	1,1531	,01881	,01330	,9841	1,3221
3	2	,9822	,04624	,03270	,5667	1,3977
7	2	1,7927	,24296	,17180	-,3902	3,9756
Total	6	1,3093	,39791	,16245	,8918	1,7269

Descriptives

AA1

	Minimum	Maximum
1	1,14	1,17
3	,95	1,01
7	1,62	1,96
Total	,95	1,96

Test of Homogeneity of Variances

AA1

Levene Statistic	df1	df2	Sig.
.	2	.	.

ANOVA

AA1

	Sum of Squares	df	Mean Square	F	Sig.
Between Groups	,730	2	,365	17,802	,022
Within Groups	,062	3	,021		
Total	,792	5			

Post Hoc Tests

Multiple Comparisons

Dependent Variable: AA1

	(I) Idő	(J) Idő	Mean Difference (I-J)	Std. Error	Sig.	95% Confidence Interval	
						Lower Bound	Upper Bound
Tamhane	1	3	,17090	,03530	,233	-,4166	,7584
		7	-,63960	,17231	,418	-6,8514	5,5722
	3	1	-,17090	,03530	,233	-,7584	,4166
		7	-,81050	,17488	,325	-6,0264	4,4054
	7	1	,63960	,17231	,418	-5,5722	6,8514
		3	,81050	,17488	,325	-4,4054	6,0264

Homogeneous Subsets

AA1

	Idő	N	Subset for alpha = 0.05	
			1	2
Duncan[a]	3	2	,9822	
	1	2	1,1531	
	7	2		1,7927
	Sig.		,318	1,000

Means for groups in homogeneous subsets are displayed.

a. Uses Harmonic Mean Sample Size = 2,000.
