# Supplementary material for: Effect of cadmium stress on certain physiological parameters, antioxidative enzyme activities and biophoton emission of leaves in barley (Hordeum vulgare L.) seedlings
Source: PLoS One. 2020 Nov 3;15(11):e0240470. doi: 10.1371/journal.pone.0240470 (PMC7608874; doi:10.1371/journal.pone.0240470)
Supplement: S1 File — (ZIP) [file pone.0240470.s003.zip › stat result time-300 Cd content leaf.pdf]

```
ONEWAY Kadmiumtartlev BY Idő
  /STATISTICS DESCRIPTIVES HOMOGENEITY
  /PLOT MEANS
  /MISSING ANALYSIS
  /POSTHOC=DUNCAN T2 ALPHA(0.05).
```

Oneway

[DataSet1] H:\Jócsák\01 Növényélettan\árpa vizsgálatok\PhD téma folytatása \MGHgyökér_1.sav

Descriptives

Kadmiumtartlev

	N	Mean	Std. Deviation	Std. Error	95% Confidence Interval for Mean	
					Lower Bound	Upper Bound
0	3	,5367	,03055	,01764	,4608	,6126
1	3	49,6633	3,04894	1,76031	42,0893	57,2373
3	3	105,1067	10,39908	6,00391	79,2739	130,9394
7	3	143,7467	13,75321	7,94042	109,5818	177,9115
Total	12	74,7633	57,26355	16,53056	38,3798	111,1469

Descriptives

Kadmiumtartlev

	Minimum	Maximum
0	,51	,57
1	47,56	53,16
3	95,14	115,89
7	130,53	157,98
Total	,51	157,98

Test of Homogeneity of Variances

Kadmiumtartlev

Levene Statistic	df1	df2	Sig.
2,637	3	8	,121

ANOVA

Kadmiumtartlev

	Sum of Squares	df	Mean Square	F	Sig.
Between Groups	35457,079	3	11819,026	154,200	,000
Within Groups	613,177	8	76,647		
Total	36070,256	11			

Post Hoc Tests

Multiple Comparisons

Dependent Variable: Kadmiumtartlev

	(I) Idő	(J) Idő	Mean Difference (I-J)	Std. Error	Sig.	95% Confidence Interval	
						Lower Bound	Upper Bound
Tamhane	0	1	-49,12667*	1,76039	,008	-68,0781	-30,1752
		3	-104,57000*	6,00394	,020	-169,2261	-39,9139
		7	-143,21000*	7,94044	,018	-228,7213	-57,6987
	1	0	49,12667*	1,76039	,008	30,1752	68,0781
		3	-55,44333*	6,25665	,044	-107,8755	-3,0111
		7	-94,08333*	8,13320	,031	-169,0930	-19,0737
	3	0	104,57000*	6,00394	,020	39,9139	169,2261
		1	55,44333*	6,25665	,044	3,0111	107,8755
		7	-38,64000	9,95476	,116	-89,2816	12,0016
	7	0	143,21000*	7,94044	,018	57,6987	228,7213
		1	94,08333*	8,13320	,031	19,0737	169,0930
		3	38,64000	9,95476	,116	-12,0016	89,2816

*. The mean difference is significant at the 0.05 level.

Homogeneous Subsets

Kadmiumtartlev

	Idő	N	Subset for alpha = 0.05			
			1	2	3	4
Duncan[a]	0	3	,5367			
	1	3		49,6633		
	3	3			105,1067	
	7	3				143,7467
	Sig.		1,000	1,000	1,000	1,000

Means for groups in homogeneous subsets are displayed.

a. Uses Harmonic Mean Sample Size = 3,000.

Means Plots

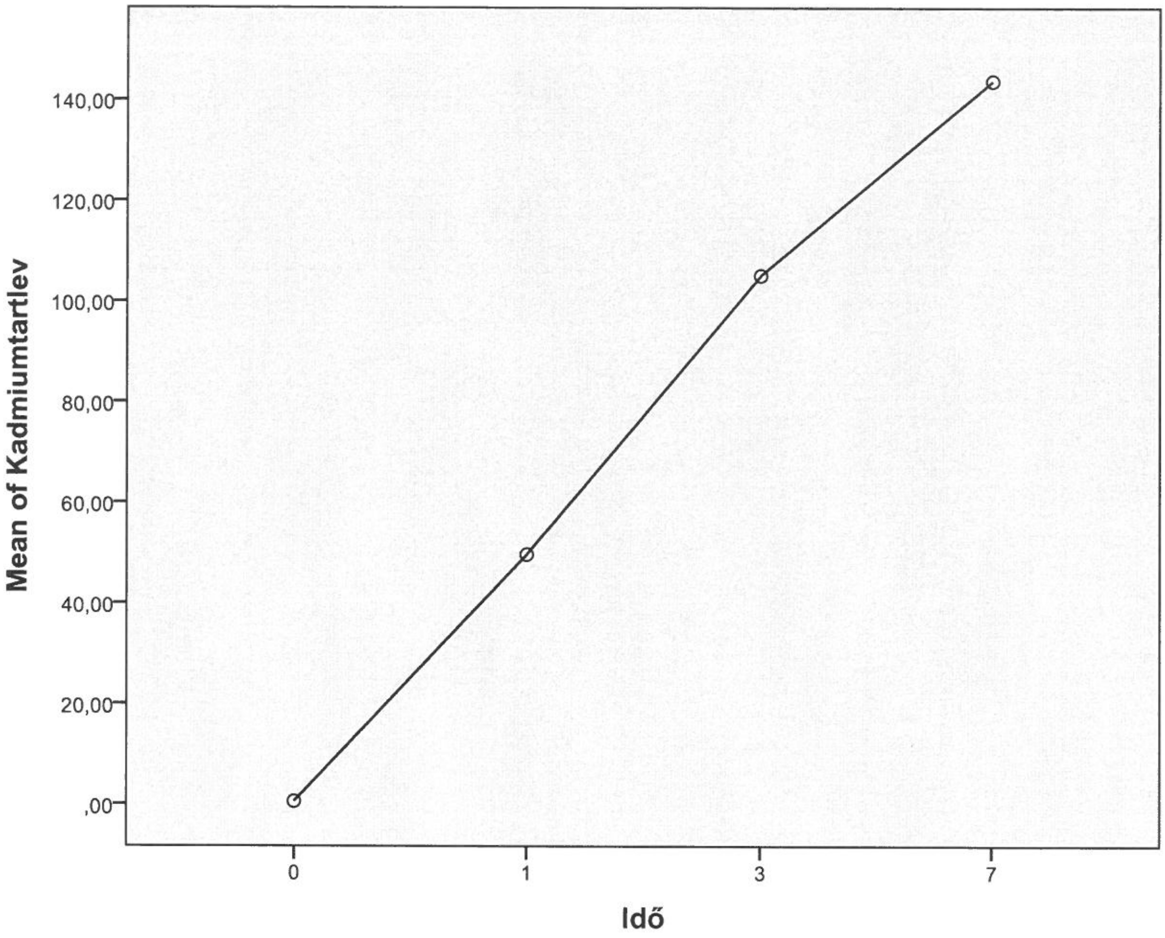

Mean of Kadmiumtartlev
140,00
120,00
100,00
80,00
60,00
40,00
20,00
,00
0
1
3
7
Idő
