# Supplementary material for: Effect of cadmium stress on certain physiological parameters, antioxidative enzyme activities and biophoton emission of leaves in barley (Hordeum vulgare L.) seedlings
Source: PLoS One. 2020 Nov 3;15(11):e0240470. doi: 10.1371/journal.pone.0240470 (PMC7608874; doi:10.1371/journal.pone.0240470)
Supplement: S1 File — (ZIP) [file pone.0240470.s003.zip › stat result time-300 Cd MDA-enzyme leaf.pdf]

```
ONEWAY MDHlevél GPXlevél APXlevél GRlevél BY Idő
  /STATISTICS DESCRIPTIVES HOMOGENEITY
  /MISSING ANALYSIS
  /POSTHOC=DUNCAN T2 ALPHA(0.05).
```

Oneway

[DataSet2] H:\Jócsák\01 Növényélettan\árpa vizsgálatok\PhD téma folytatása \MGHgyökér_1.sav

Descriptives

		N	Mean	Std. Deviation	Std. Error	95% Confidence ...
						Lower Bound
MDHlevél	0	3	21,5415	1,68115	,97061	17,3652
	1	3	20,5489	1,02581	,59225	18,0007
	3	3	19,6258	3,60302	2,08020	10,6754
	7	3	48,9918	7,30218	4,21591	30,8522
	Total	12	27,6770	13,35923	3,85648	19,1890
GPXlevél	0	3	,6837	,04569	,02638	,5702
	1	3	,8756	,09764	,05637	,6331
	3	3	1,9438	,18138	,10472	1,4932
	7	3	2,2273	,12948	,07475	1,9056
	Total	12	1,4326	,70157	,20253	,9868
APXlevél	0	3	,1575	,01006	,00581	,1325
	1	3	,1654	,02994	,01728	,0910
	3	3	,2241	,01458	,00842	,1879
	7	3	,2582	,02216	,01280	,2032
	Total	12	,2013	,04702	,01357	,1714
GRlevél	0	3	,004596	,0009166	,0005292	,002319
	1	3	,005033	,0016392	,0009464	,000961
	3	3	,005866	,0026681	,0015404	-,000762
	7	3	,007266	,0017147	,0009900	,003006
	Total	12	,005690	,0018973	,0005477	,004485

Descriptives

		95% Confidence ...		
		Upper Bound	Minimum	Maximum
MDHlevél	0	25,7177	19,60	22,59
	1	23,0972	19,75	21,71
	3	28,5762	17,23	23,77
	7	67,1314	40,56	53,21
	Total	36,1650	17,23	53,21
GPXlevél	0	,7972	,64	,73
	1	1,1182	,79	,98
	3	2,3943	1,75	2,11
	7	2,5489	2,09	2,35
	Total	1,8783	,64	2,35
APXlevél	0	,1825	,15	,17
	1	,2397	,14	,20
	3	,2604	,21	,23
	7	,3133	,24	,28
	Total	,2312	,14	,28
GRlevél	0	,006873	,0037	,0055
	1	,009105	,0040	,0069
	3	,012494	,0035	,0088
	7	,011525	,0054	,0088
	Total	,006896	,0035	,0088

Test of Homogeneity of Variances

	Levene Statistic	df1	df2	Sig.
MDHlevél	7,156	3	8	,012
GPXlevél	1,200	3	8	,370
APXlevél	1,731	3	8	,238
GRlevél	1,250	3	8	,355

ANOVA

		Sum of Squares	df	Mean Square	F	Sig.
MDHlevél	Between Groups	1822,794	3	607,598	34,630	,000
	Within Groups	140,364	8	17,546		
	Total	1963,158	11			
GPXlevél	Between Groups	5,292	3	1,764	115,125	,000
	Within Groups	,123	8	,015		
	Total	5,414	11			
APXlevél	Between Groups	,021	3	,007	16,392	,001
	Within Groups	,003	8	,000		
	Total	,024	11			
GRlevél	Between Groups	,000	3	,000	1,220	,364
	Within Groups	,000	8	,000		
	Total	,000	11			

Post Hoc Tests

Multiple Comparisons

Dependent Variable		(I) Idő	(J) Idő	Mean Difference (I-J)	Std. Error	Sig.	95% ...
							Lower Bound
MDHlevél	Tamhane	0	1	,99254	1,13704	,970	-5,4013
			3	1,91565	2,29550	,977	-13,1947
			7	-27,45037	4,32620	,106	-66,9215
		1	0	-,99254	1,13704	,970	-7,3863
			3	,92311	2,16287	,999	-17,4189
			7	-28,44291	4,25731	,112	-71,3449
		3	0	-1,91565	2,29550	,977	-17,0260
			1	-,92311	2,16287	,999	-19,2651
			7	-29,36602	4,70119	,053	-59,2912
		7	0	27,45037	4,32620	,106	-12,0207
			1	28,44291	4,25731	,112	-14,4591
			3	29,36602	4,70119	,053	-,5591
GPXlevél	Tamhane	0	1	-,19194	,06224	,302	-,6009
			3	-1,26008*	,10799	,027	-2,2179
			7	-1,54358*	,07927	,005	-2,1537
		1	0	,19194	,06224	,302	-,2170
			3	-1,06814*	,11893	,016	-1,7867
			7	-1,35164*	,09363	,001	-1,8284
		3	0	1,26008*	,10799	,027	,3023
			1	1,06814*	,11893	,016	,3495
			7	-,28350	,12867	,467	-,9532
		7	0	1,54358*	,07927	,005	,9335
			1	1,35164*	,09363	,001	,8749
			3	,28350	,12867	,467	-,3862

Multiple Comparisons

Dependent Variable		(I) Idő	(J) Idő	95% ... Upper Bound
MDHlevél	Tamhane	0	1	7,3863
			3	17,0260
			7	12,0207
		1	0	5,4013
			3	19,2651
			7	14,4591
		3	0	13,1947
			1	17,4189
			7	,5591
		7	0	66,9215
			1	71,3449
			3	59,2912
GPXlevél	Tamhane	0	1	,2170
			3	-,3023
			7	-,9335
		1	0	,6009
			3	-,3495
			7	-,8749
		3	0	2,2179
			1	1,7867
			7	,3862
		7	0	2,1537
			1	1,8284
			3	,9532

Multiple Comparisons

Dependent Variable		(I) Idő	(J) Idő	Mean Difference (I-J)	Std. Error	Sig.	95% ...
							Lower Bound
APXlevél	Tamhane	0	1	-,00784	,01823	,999	-,1516
			3	-,06663*	,01023	,025	-,1207
			7	-,10071*	,01405	,042	-,1947
		1	0	,00784	,01823	,999	-,1359
			3	-,05879	,01922	,300	-,1821
			7	-,09287	,02150	,086	-,2031
		3	0	,06663*	,01023	,025	,0126
			1	,05879	,01922	,300	-,0645
			7	-,03407	,01532	,471	-,1169
		7	0	,10071*	,01405	,042	,0067
			1	,09287	,02150	,086	-,0174
			3	,03407	,01532	,471	-,0487
GRlevél	Tamhane	0	1	-,0004376	,0010843	,999	-,006844
			3	-,0012703	,0016288	,985	-,013975
			7	-,0026700	,0011225	,455	-,009480
		1	0	,0004376	,0010843	,999	-,005969
			3	-,0008327	,0018079	,999	-,010961
			7	-,0022324	,0013695	,693	-,008845
		3	0	,0012703	,0016288	,985	-,011434
			1	,0008327	,0018079	,999	-,009296
			7	-,0013997	,0018311	,983	-,011417
		7	0	,0026700	,0011225	,455	-,004141
			1	,0022324	,0013695	,693	-,004381
			3	,0013997	,0018311	,983	-,008617

Multiple Comparisons

Dependent Variable		(I) Idő	(J) Idő	95% ... Upper Bound
APXlevél	Tamhane	0	1	,1359
			3	-,0126
			7	-,0067
		1	0	,1516
			3	,0645
			7	,0174
		3	0	,1207
			1	,1821
			7	,0487
		7	0	,1947
			1	,2031
			3	,1169
GRlevél	Tamhane	0	1	,005969
			3	,011434
			7	,004141
		1	0	,006844
			3	,009296
			7	,004381
		3	0	,013975
			1	,010961
			7	,008617
		7	0	,009480
			1	,008845
			3	,011417

*. The mean difference is significant at the 0.05 level.

Homogeneous Subsets

MDHlevél

	Idő	N	Subset for alpha = 0.05: 1	2
Duncan[a]	3	3	19,6258	
	1	3	20,5489	
	0	3	21,5415	
	7	3		48,9918
	Sig.		,605	1,000

Means for groups in homogeneous subsets are displayed.

a. Uses Harmonic Mean Sample Size = 3,000.

GPXlevél

	Idő	N	Subset for alpha = 0.05		
			1	2	3
Duncan[a]	0	3	,6837		
	1	3	,8756		
	3	3		1,9438	
	7	3			2,2273
	Sig.		,094	1,000	1,000

Means for groups in homogeneous subsets are displayed.

a. Uses Harmonic Mean Sample Size = 3,000.

APXlevél

	Idő	N	Subset for alpha = 0.05	
			1	2
Duncan[a]	0	3	,1575	
	1	3	,1654	
	3	3		,2241
	7	3		,2582
	Sig.		,654	,078

Means for groups in homogeneous subsets are displayed.

a. Uses Harmonic Mean Sample Size = 3,000.

GRlevél

	Idő	N	Subset for alpha = 0.05
			1
Duncan[a]	0	3	,004596
	1	3	,005033
	3	3	,005866
	7	3	,007266
	Sig.		,134

Means for groups in homogeneous subsets are displayed.

a. Uses Harmonic Mean Sample Size = 3,000.
