# Supplementary material for: Effect of cadmium stress on certain physiological parameters, antioxidative enzyme activities and biophoton emission of leaves in barley (Hordeum vulgare L.) seedlings
Source: PLoS One. 2020 Nov 3;15(11):e0240470. doi: 10.1371/journal.pone.0240470 (PMC7608874; doi:10.1371/journal.pone.0240470)
Supplement: S1 File — (ZIP) [file pone.0240470.s003.zip › stat result time-300 Cd SPAD leaf.pdf]

```
ONEWAY SPAD BY Idő
  /STATISTICS DESCRIPTIVES HOMOGENEITY
  /PLOT MEANS
  /MISSING ANALYSIS
  /POSTHOC=DUNCAN T2 ALPHA(0.05).
```

Oneway

```
[DataSet1] H:\Jócsák\01 Növényélettan\árpa vizsgálatok\PhD téma folytatása
\SPAD\SPAD-two-way-anova.sav
```

Descriptives

SPAD

	N	Mean	Std. Deviation	Std. Error	95% Confidence Interval for Mean	
					Lower Bound	Upper Bound
0	100	28,0390	3,13310	,31331	27,4173	28,6607
1	100	28,3148	3,61216	,36122	27,5981	29,0315
3	100	21,0210	4,96528	,49653	20,0358	22,0062
7	100	13,9730	6,86247	,68625	12,6113	15,3347
Total	400	22,8369	7,63518	,38176	22,0864	23,5875

Descriptives

SPAD

	Minimum	Maximum
0	22,10	38,90
1	20,20	38,90
3	5,60	32,50
7	1,60	28,30
Total	1,60	38,90

Test of Homogeneity of Variances

SPAD

Levene Statistic	df1	df2	Sig.
25,981	3	396	,000

ANOVA

SPAD

	Sum of Squares	df	Mean Square	F	Sig.
Between Groups	13893,545	3	4631,182	195,798	,000
Within Groups	9366,545	396	23,653		
Total	23260,090	399			

Post Hoc Tests

Multiple Comparisons

Dependent Variable: SPAD

	(I) Idő	(J) Idő	Mean Difference (I-J)	Std. Error	Sig.	95% Confidence Interval	
						Lower Bound	Upper Bound
Tamhane	0	1	-,27580	,47816	,993	-1,5468	,9952
		3	7,01800*	,58711	,000	5,4548	8,5812
		7	14,06600*	,75439	,000	12,0524	16,0796
	1	0	,27580	,47816	,993	-,9952	1,5468
		3	7,29380*	,61402	,000	5,6604	8,9272
		7	14,34180*	,77551	,000	12,2741	16,4095
	3	0	-7,01800*	,58711	,000	-8,5812	-5,4548
		1	-7,29380*	,61402	,000	-8,9272	-5,6604
		7	7,04800*	,84704	,000	4,7947	9,3013
	7	0	-14,06600*	,75439	,000	-16,0796	-12,0524
		1	-14,34180*	,77551	,000	-16,4095	-12,2741
		3	-7,04800*	,84704	,000	-9,3013	-4,7947

*. The mean difference is significant at the 0.05 level.

Homogeneous Subsets

SPAD

	Idő	N	Subset for alpha = 0.05		
			1	2	3
Duncan[a]	7	100	13,9730		
	3	100		21,0210	
	0	100			28,0390
	1	100			28,3148
	Sig.		1,000	1,000	,689

Means for groups in homogeneous subsets are displayed.

a. Uses Harmonic Mean Sample Size = 100,000.

Means Plots

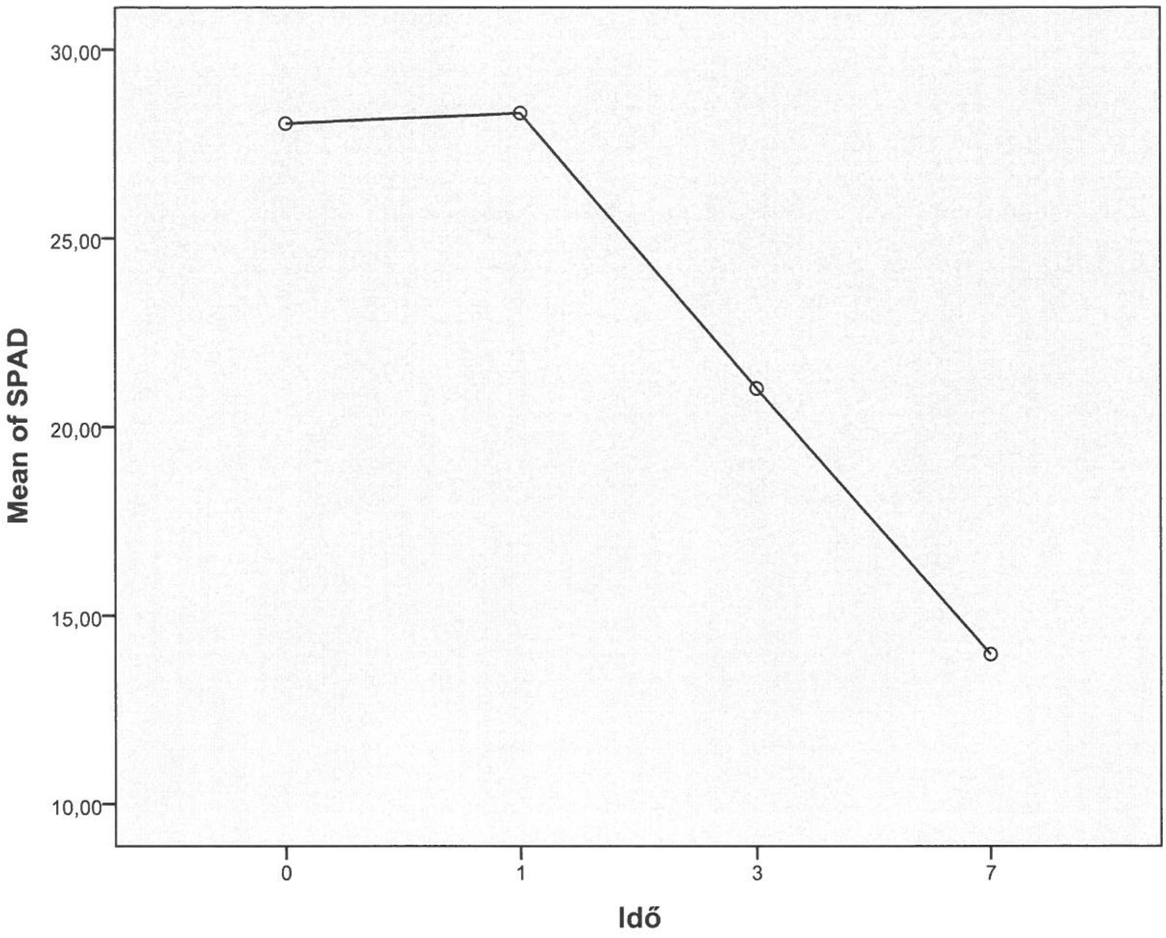
30,00
25,00
20,00
15,00
10,00
Mean of SPAD
0
1
3
7
Idő
