# Supplementary material for: Effect of cadmium stress on certain physiological parameters, antioxidative enzyme activities and biophoton emission of leaves in barley (Hordeum vulgare L.) seedlings
Source: PLoS One. 2020 Nov 3;15(11):e0240470. doi: 10.1371/journal.pone.0240470 (PMC7608874; doi:10.1371/journal.pone.0240470)
Supplement: S1 File — (ZIP) [file pone.0240470.s003.zip › stat result time-50 Cd AA leaf-10 Cd SPAD leaf.pdf]

```
ONEWAY AA1 BY Idő
  /STATISTICS DESCRIPTIVES HOMOGENEITY
  /MISSING ANALYSIS
  /POSTHOC=DUNCAN T2 ALPHA(0.05).
```

Oneway

[DataSet2] H:\Jócsák\01 Növényélettan\árpa vizsgálatok\PhD téma folytatása \Visi É árpa c vit meghatározás\aszkorbinsav mg-g fr tömeg.sav

Descriptives

AA1

	N	Mean	Std. Deviation	Std. Error	95% Confidence Interval for Mean	
					Lower Bound	Upper Bound
1	2	,6419	,01351	,00955	,5205	,7632
3	2	,8233	,02885	,02040	,5641	1,0825
7	2	1,3117	,20549	,14530	-,5345	3,1579
Total	6	,9256	,32353	,13208	,5861	1,2651

Descriptives

AA1

	Minimum	Maximum
1	,63	,65
3	,80	,84
7	1,17	1,46
Total	,63	1,46

Test of Homogeneity of Variances

AA1

Levene Statistic	df1	df2	Sig.
.	2	.	.

ANOVA

AA1

	Sum of Squares	df	Mean Square	F	Sig.
Between Groups	,480	2	,240	16,655	,024
Within Groups	,043	3	,014		
Total	,523	5			

Post Hoc Tests

Multiple Comparisons

Dependent Variable: AA1

	(I) Idő	(J) Idő	Mean Difference (I-J)	Std. Error	Sig.	95% Confidence Interval	
						Lower Bound	Upper Bound
Tamhane	1	3	-,18145	,02252	,108	-,4996	,1367
		7	-,66985	,14561	,352	-5,9785	4,6388
	3	1	,18145	,02252	,108	-,1367	,4996
		7	-,48840	,14673	,445	-5,3269	4,3501
	7	1	,66985	,14561	,352	-4,6388	5,9785
		3	,48840	,14673	,445	-4,3501	5,3269

Homogeneous Subsets

AA1

	Idő	N	Subset for alpha = 0.05	
			1	2
Duncan[a]	1	2	,6419	
	3	2	,8233	
	7	2		1,3117
	Sig.		,228	1,000

Means for groups in homogeneous subsets are displayed.

a. Uses Harmonic Mean Sample Size = 2,000.

```
ONEWAY SPAD BY Idő
  /STATISTICS DESCRIPTIVES HOMOGENEITY
  /PLOT MEANS
  /MISSING ANALYSIS
  /POSTHOC=DUNCAN T2 ALPHA(0.05).
```

Oneway

[DataSet1] H:\Jócsák\01 Növényélettan\árpa vizsgálatok\PhD téma folytatása \SPAD\SPAD-two-way-anova.sav

Descriptives

SPAD

	N	Mean	Std. Deviation	Std. Error	95% Confidence Interval for Mean	
					Lower Bound	Upper Bound
0	100	28,0390	3,13310	,31331	27,4173	28,6607
1	100	28,4333	3,35001	,33500	27,7686	29,0980
3	100	24,8020	5,57804	,55780	23,6952	25,9088
7	100	26,4710	5,06308	,50631	25,4664	27,4756
Total	400	26,9363	4,62203	,23110	26,4820	27,3907

Descriptives

SPAD

	Minimum	Maximum
0	22,10	38,90
1	22,10	38,90
3	10,30	35,30
7	15,70	36,10
Total	10,30	38,90

Test of Homogeneity of Variances

SPAD

Levene Statistic	df1	df2	Sig.
19,185	3	396	,000

ANOVA

SPAD

	Sum of Squares	df	Mean Square	F	Sig.
Between Groups	822,870	3	274,290	14,104	,000
Within Groups	7701,035	396	19,447		
Total	8523,904	399			

Post Hoc Tests

Multiple Comparisons

Dependent Variable: SPAD

	(I) Idő	(J) Idő	Mean Difference (I-J)	Std. Error	Sig.	95% Confidence Interval	
						Lower Bound	Upper Bound
Tamhane	0	1	-,39430	,45868	,949	-1,6133	,8247
		3	3,23700*	,63977	,000	1,5321	4,9419
		7	1,56800	,59541	,054	-,0175	3,1535
	1	0	,39430	,45868	,949	-,8247	1,6133
		3	3,63130*	,65067	,000	1,8982	5,3644
		7	1,96230*	,60710	,009	,3464	3,5782
	3	0	-3,23700*	,63977	,000	-4,9419	-1,5321
		1	-3,63130*	,65067	,000	-5,3644	-1,8982
		7	-1,66900	,75332	,156	-3,6712	,3332
	7	0	-1,56800	,59541	,054	-3,1535	,0175
		1	-1,96230*	,60710	,009	-3,5782	-,3464
		3	1,66900	,75332	,156	-,3332	3,6712

*. The mean difference is significant at the 0.05 level.

Homogeneous Subsets

SPAD

	Idő	N	Subset for alpha = 0.05		
			1	2	3
Duncan[a]	3	100	24,8020		
	7	100		26,4710	
	0	100			28,0390
	1	100			28,4333
	Sig.		1,000	1,000	,528

Means for groups in homogeneous subsets are displayed.

a. Uses Harmonic Mean Sample Size = 100,000.

Means Plots

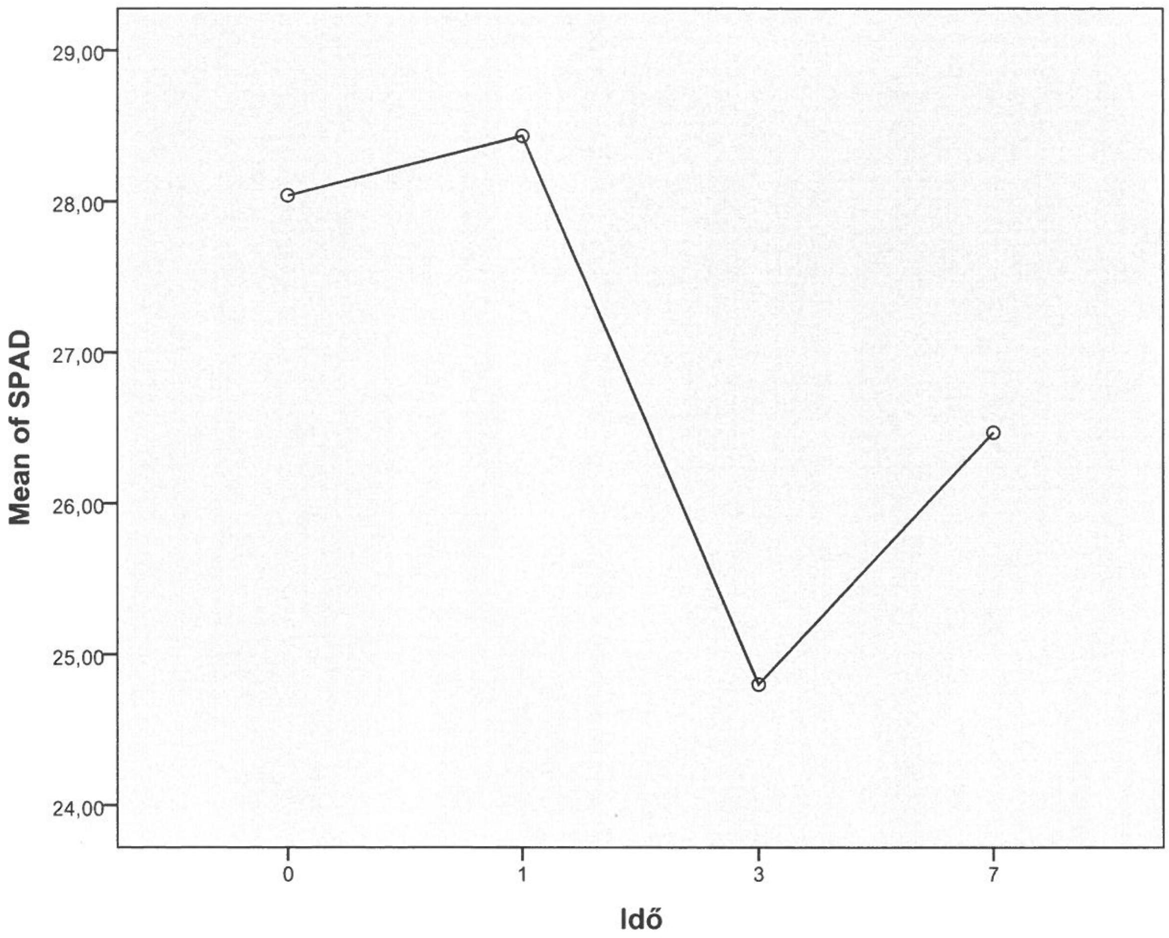

Mean of SPAD
29,00
28,00
27,00
26,00
25,00
24,00
0
1
3
7
Idő
