# Supplementary material for: Effect of cadmium stress on certain physiological parameters, antioxidative enzyme activities and biophoton emission of leaves in barley (Hordeum vulgare L.) seedlings
Source: PLoS One. 2020 Nov 3;15(11):e0240470. doi: 10.1371/journal.pone.0240470 (PMC7608874; doi:10.1371/journal.pone.0240470)
Supplement: S1 File — (ZIP) [file pone.0240470.s003.zip › stat result time-50 Cd content leaf.pdf]

```
ONEWAY Kadmiumtartlev BY Idő
  /STATISTICS DESCRIPTIVES HOMOGENEITY
  /PLOT MEANS
  /MISSING ANALYSIS
  /POSTHOC=DUNCAN T2 ALPHA(0.05).
```

Oneway

```
[DataSet1] H:\Jócsák\01 Növényélettan\árpa vizsgálatok\PhD téma folytatása
\MGHgyökér_1.sav
```

Descriptives

Kadmiumtartlev

	N	Mean	Std. Deviation	Std. Error	95% Confidence Interval for Mean	
					Lower Bound	Upper Bound
0	3	,5367	,03055	,01764	,4608	,6126
1	3	12,8633	,65287	,37693	11,2415	14,4851
3	3	39,3700	3,64600	2,10502	30,3128	48,4272
7	3	49,3667	7,59485	4,38489	30,5000	68,2333
Total	12	25,5342	20,83994	6,01597	12,2931	38,7752

Descriptives

Kadmiumtartlev

	Minimum	Maximum
0	,51	,57
1	12,16	13,45
3	36,56	43,49
7	41,62	56,80
Total	,51	56,80

Test of Homogeneity of Variances

Kadmiumtartlev

Levene Statistic	df1	df2	Sig.
3,400	3	8	,074

ANOVA

Kadmiumtartlev

	Sum of Squares	df	Mean Square	F	Sig.
Between Groups	4634,530	3	1544,843	86,543	,000
Within Groups	142,804	8	17,851		
Total	4777,334	11			

Post Hoc Tests

Multiple Comparisons

Dependent Variable: Kadmiumtartlev

	(I) Idő	(J) Idő	Mean Difference (I-J)	Std. Error	Sig.	95% Confidence Interval Lower Bound	Upper Bound
Tamhane	0	1	-12,32667*	,37734	,005	-16,3594	-8,2940
		3	-38,83333*	2,10509	,017	-61,4980	-16,1686
		7	-48,83000*	4,38492	,047	-96,0497	-1,6103
	1	0	12,32667*	,37734	,005	8,2940	16,3594
		3	-26,50667*	2,13850	,030	-47,2414	-5,7720
		7	-36,50333	4,40106	,079	-82,7030	9,6963
	3	0	38,83333*	2,10509	,017	16,1686	61,4980
		1	26,50667*	2,13850	,030	5,7720	47,2414
		7	-9,99667	4,86398	,584	-41,4729	21,4796
	7	0	48,83000*	4,38492	,047	1,6103	96,0497
		1	36,50333	4,40106	,079	-9,6963	82,7030
		3	9,99667	4,86398	,584	-21,4796	41,4729

*. The mean difference is significant at the 0.05 level.

Homogeneous Subsets

Kadmiumtartlev

	Idő	N	Subset for alpha = 0.05 1	2	3	4
Duncan[a]	0	3	,5367			
	1	3		12,8633		
	3	3			39,3700	
	7	3				49,3667
	Sig.		1,000	1,000	1,000	1,000

Means for groups in homogeneous subsets are displayed.

a. Uses Harmonic Mean Sample Size = 3,000.

Means Plots

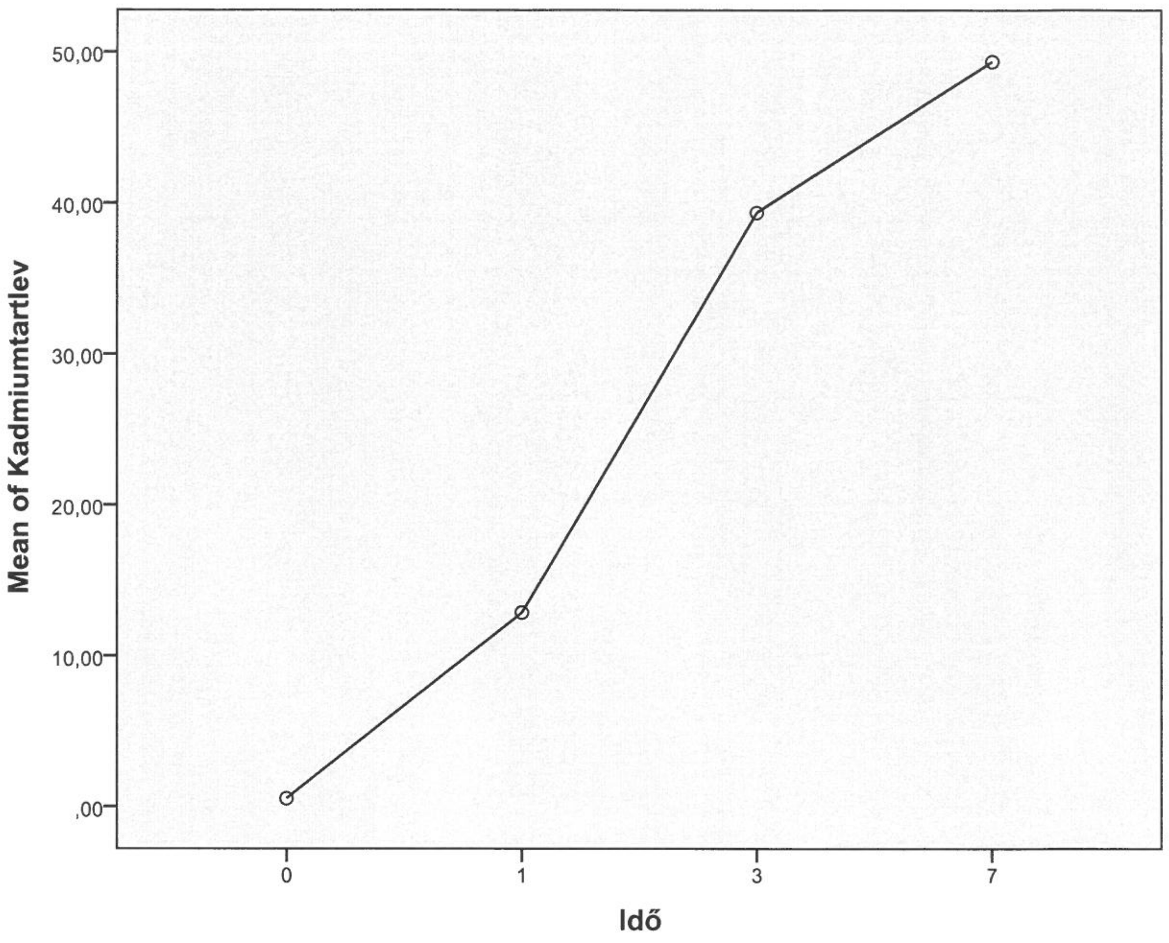
50,00
40,00
30,00
20,00
10,00
,00
Mean of Kadmiumtartlev
0
1
3
7
Idő
