# Supplementary material for: Effect of cadmium stress on certain physiological parameters, antioxidative enzyme activities and biophoton emission of leaves in barley (Hordeum vulgare L.) seedlings
Source: PLoS One. 2020 Nov 3;15(11):e0240470. doi: 10.1371/journal.pone.0240470 (PMC7608874; doi:10.1371/journal.pone.0240470)
Supplement: S1 File — (ZIP) [file pone.0240470.s003.zip › stat result time-50 Cd MDH-enzyme leaf.pdf]

```
ONEWAY MDHlevél GPXlevél APXlevél GRlevél BY Idő
  /STATISTICS DESCRIPTIVES HOMOGENEITY
  /MISSING ANALYSIS
  /POSTHOC=DUNCAN T2 ALPHA(0.05).
```

Oneway

[DataSet2] H:\Jócsák\01 Növényélettan\árpa vizsgálatok\PhD téma folytatása \MGHgyökér_1.sav

Descriptives

		N	Mean	Std. Deviation	Std. Error	95% Confidence ... Lower Bound
MDHlevél	0	3	21,5415	1,68115	,97061	17,3652
	1	3	18,8236	,33177	,19155	17,9995
	3	3	18,3174	1,99859	1,15389	13,3526
	7	3	33,1458	,75540	,43613	31,2693
	Total	12	22,9571	6,38379	1,84284	18,9010
GPXlevél	0	3	,6837	,04569	,02638	,5702
	1	3	,8689	,09979	,05761	,6210
	3	3	1,5044	,20914	,12075	,9849
	7	3	2,4211	,21858	,12619	1,8781
	Total	12	1,3695	,72250	,20857	,9105
APXlevél	0	3	,1575	,01006	,00581	,1325
	1	3	,1634	,01296	,00748	,1312
	3	3	,1989	,01489	,00860	,1619
	7	3	,2361	,02876	,01660	,1646
	Total	12	,1890	,03632	,01048	,1659
GRlevél	0	3	,004596	,0009166	,0005292	,002319
	1	3	,004905	,0014371	,0008297	,001335
	3	3	,006187	,0018519	,0010692	,001587
	7	3	,005817	,0013725	,0007924	,002407
	Total	12	,005376	,0013976	,0004034	,004488
