# Supplementary material for: Effect of cadmium stress on certain physiological parameters, antioxidative enzyme activities and biophoton emission of leaves in barley (Hordeum vulgare L.) seedlings
Source: PLoS One. 2020 Nov 3;15(11):e0240470. doi: 10.1371/journal.pone.0240470 (PMC7608874; doi:10.1371/journal.pone.0240470)
Supplement: S1 File — (ZIP) [file pone.0240470.s003.zip › stat result time-50 Cd MDH-enzyme leaf-2.pdf]

Descriptives

		95% Confidence ... Upper Bound	Minimum	Maximum
MDHlevél	0	25,7177	19,60	22,59
	1	19,6478	18,46	19,11
	3	23,2822	16,14	20,06
	7	35,0223	32,27	33,58
	Total	27,0131	16,14	33,58
GPXlevél	0	,7972	,64	,73
	1	1,1168	,78	,98
	3	2,0240	1,28	1,70
	7	2,9640	2,18	2,61
	Total	1,8286	,64	2,61
APXlevél	0	,1825	,15	,17
	1	,1956	,15	,18
	3	,2359	,19	,21
	7	,3075	,20	,26
	Total	,2120	,15	,26
GRlevél	0	,006873	,0037	,0055
	1	,008475	,0041	,0066
	3	,010787	,0043	,0080
	7	,009226	,0050	,0074
	Total	,006264	,0037	,0080

Test of Homogeneity of Variances

	Levene Statistic	df1	df2	Sig.
MDHlevél	3,240	3	8	,082
GPXlevél	1,792	3	8	,226
APXlevél	2,085	3	8	,181
GRlevél	,521	3	8	,680
