# Supplementary material for: Effect of cadmium stress on certain physiological parameters, antioxidative enzyme activities and biophoton emission of leaves in barley (Hordeum vulgare L.) seedlings
Source: PLoS One. 2020 Nov 3;15(11):e0240470. doi: 10.1371/journal.pone.0240470 (PMC7608874; doi:10.1371/journal.pone.0240470)
Supplement: S1 File — (ZIP) [file pone.0240470.s003.zip › stat result time-50 Cd MDH-enzyme leaf-3.pdf]

**ANOVA**

| | | Sum of Squares | df | Mean Square | F | Sig. |
|---|---|---|---|---|---|---|
| MDHlevél | Between Groups | 433,278 | 3 | 144,426 | 77,013 | ,000 |
| | Within Groups | 15,003 | 8 | 1,875 | | |
| | Total | 448,281 | 11 | | | |
| GPXlevél | Between Groups | 5,535 | 3 | 1,845 | 71,262 | ,000 |
| | Within Groups | ,207 | 8 | ,026 | | |
| | Total | 5,742 | 11 | | | |
| APXlevél | Between Groups | ,012 | 3 | ,004 | 12,016 | ,002 |
| | Within Groups | ,003 | 8 | ,000 | | |
| | Total | ,015 | 11 | | | |
| GRlevél | Between Groups | ,000 | 3 | ,000 | ,819 | ,519 |
| | Within Groups | ,000 | 8 | ,000 | | |
| | Total | ,000 | 11 | | | |

# Post Hoc Tests

**Multiple Comparisons**

| Dependent Variable | | (I) Idő | (J) Idő | Mean Difference (I-J) | Std. Error | Sig. | 95% ... Lower Bound |
|---|---|---|---|---|---|---|---|
| MDHlevél | Tamhane | 0 | 1 | 2,71782 | ,98933 | ,476 | -6,6789 |
| | | | 3 | 3,22404 | 1,50783 | ,473 | -4,2008 |
| | | | 7 | -11,60435* | 1,06409 | ,014 | -18,7677 |
| | | 1 | 0 | -2,71782 | ,98933 | ,476 | -12,1145 |
| | | | 3 | ,50621 | 1,16968 | ,999 | -10,9931 |
| | | | 7 | -14,32218* | ,47634 | ,001 | -17,5722 |
| | | 3 | 0 | -3,22404 | 1,50783 | ,473 | -10,6489 |
| | | | 1 | -,50621 | 1,16968 | ,999 | -12,0055 |
| | | | 7 | -14,82839* | 1,23356 | ,015 | -23,9886 |
| | | 7 | 0 | 11,60435* | 1,06409 | ,014 | 4,4410 |
| | | | 1 | 14,32218* | ,47634 | ,001 | 11,0721 |
| | | | 3 | 14,82839* | 1,23356 | ,015 | 5,6681 |
| GPXlevél | Tamhane | 0 | 1 | -,18520 | ,06337 | ,339 | -,6070 |
| | | | 3 | -,82074 | ,12360 | ,099 | -1,9652 |
| | | | 7 | -1,73740* | ,12892 | ,023 | -2,9450 |
| | | 1 | 0 | ,18520 | ,06337 | ,339 | -,2366 |
| | | | 3 | -,63554 | ,13379 | ,112 | -1,5045 |
| | | | 7 | -1,55220* | ,13872 | ,012 | -2,4773 |
| | | 3 | 0 | ,82074 | ,12360 | ,099 | -,3237 |
| | | | 1 | ,63554 | ,13379 | ,112 | -,2334 |
| | | | 7 | -,91666* | ,17466 | ,037 | -1,7600 |
| | | 7 | 0 | 1,73740* | ,12892 | ,023 | ,5298 |
| | | | 1 | 1,55220* | ,13872 | ,012 | ,6271 |
| | | | 3 | ,91666* | ,17466 | ,037 | ,0733 |
