# Supplementary material for: Effect of cadmium stress on certain physiological parameters, antioxidative enzyme activities and biophoton emission of leaves in barley (Hordeum vulgare L.) seedlings
Source: PLoS One. 2020 Nov 3;15(11):e0240470. doi: 10.1371/journal.pone.0240470 (PMC7608874; doi:10.1371/journal.pone.0240470)
Supplement: S1 File — (ZIP) [file pone.0240470.s003.zip › stat result time-50 Cd MDH-enzyme leaf-4.pdf]

Multiple Comparisons

Dependent Variable		(I) Idő	(J) Idő	95% ... Upper Bound
MDHlevél	Tamhane	0	1	12,1145
			3	10,6489
			7	-4,4410
		1	0	6,6789
			3	12,0055
			7	-11,0721
		3	0	4,2008
			1	10,9931
			7	-5,6681
		7	0	18,7677
			1	17,5722
			3	23,9886
GPXlevél	Tamhane	0	1	,2366
			3	,3237
			7	-,5298
		1	0	,6070
			3	,2334
			7	-,6271
		3	0	1,9652
			1	1,5045
			7	-,0733
		7	0	2,9450
			1	2,4773
			3	1,7600
