# Supplementary material for: Effect of cadmium stress on certain physiological parameters, antioxidative enzyme activities and biophoton emission of leaves in barley (Hordeum vulgare L.) seedlings
Source: PLoS One. 2020 Nov 3;15(11):e0240470. doi: 10.1371/journal.pone.0240470 (PMC7608874; doi:10.1371/journal.pone.0240470)
Supplement: S1 File — (ZIP) [file pone.0240470.s003.zip › stat result time-50 Cd MDH-enzyme leaf-5.pdf]

Multiple Comparisons

Dependent Variable		(I) Idő	(J) Idő	Mean Difference (I-J)	Std. Error	Sig.	95% ...
							Lower Bound
APXlevél	Tamhane	0	1	-,00589	,00947	,994	-,0536
			3	-,04140	,01038	,119	-,0967
			7	-,07854	,01759	,171	-,2145
		1	0	,00589	,00947	,994	-,0418
			3	-,03551	,01139	,200	-,0912
			7	-,07264	,01821	,180	-,1950
		3	0	,04140	,01038	,119	-,0139
			1	,03551	,01139	,200	-,0202
			7	-,03714	,01870	,599	-,1528
		7	0	,07854	,01759	,171	-,0574
			1	,07264	,01821	,180	-,0497
			3	,03714	,01870	,599	-,0785
GRlevél	Tamhane	0	1	-,0003093	,0009841	1,000	-,005714
			3	-,0015913	,0011930	,857	-,009171
			7	-,0012209	,0009529	,859	-,006334
		1	0	,0003093	,0009841	1,000	-,005095
			3	-,0012819	,0013534	,953	-,008104
			7	-,0009116	,0011473	,978	-,006452
		3	0	,0015913	,0011930	,857	-,005989
			1	,0012819	,0013534	,953	-,005540
			7	,0003704	,0013308	1,000	-,006451
		7	0	,0012209	,0009529	,859	-,003892
			1	,0009116	,0011473	,978	-,004629
			3	-,0003704	,0013308	1,000	-,007192
