# Supplementary material for: Effect of cadmium stress on certain physiological parameters, antioxidative enzyme activities and biophoton emission of leaves in barley (Hordeum vulgare L.) seedlings
Source: PLoS One. 2020 Nov 3;15(11):e0240470. doi: 10.1371/journal.pone.0240470 (PMC7608874; doi:10.1371/journal.pone.0240470)
Supplement: S1 File — (ZIP) [file pone.0240470.s003.zip › stat result time-50 Cd MDH-enzyme leaf-6.pdf]

Multiple Comparisons

Dependent Variable		(I) Idő	(J) Idő	95% ... Upper Bound
APXlevél	Tamhane	0	1	,0418
			3	,0139
			7	,0574
		1	0	,0536
			3	,0202
			7	,0497
		3	0	,0967
			1	,0912
			7	,0785
		7	0	,2145
			1	,1950
			3	,1528
GRlevél	Tamhane	0	1	,005095
			3	,005989
			7	,003892
		1	0	,005714
			3	,005540
			7	,004629
		3	0	,009171
			1	,008104
			7	,007192
		7	0	,006334
			1	,006452
			3	,006451

*. The mean difference is significant at the 0.05 level.

Homogeneous Subsets

MDHlevél

	Idő	N	Subset for alpha = 0.05 1	2	3
Duncan[a]	3	3	18,3174		
	1	3	18,8236		
	0	3		21,5415	
	7	3			33,1458
	Sig.		,663	1,000	1,000

Means for groups in homogeneous subsets are displayed.

a. Uses Harmonic Mean Sample Size = 3,000.
