# Supplementary material for: Effect of cadmium stress on certain physiological parameters, antioxidative enzyme activities and biophoton emission of leaves in barley (Hordeum vulgare L.) seedlings
Source: PLoS One. 2020 Nov 3;15(11):e0240470. doi: 10.1371/journal.pone.0240470 (PMC7608874; doi:10.1371/journal.pone.0240470)
Supplement: S1 File — (ZIP) [file pone.0240470.s003.zip › stat result time-50 Cd MDH-enzyme leaf-7.pdf]

GPXlevél

	Idő	N	Subset for alpha = 0.05		
			1	2	3
Duncan[a]	0	3	,6837		
	1	3	,8689		
	3	3		1,5044	
	7	3			2,4211
	Sig.		,196	1,000	1,000

Means for groups in homogeneous subsets are displayed.

a. Uses Harmonic Mean Sample Size = 3,000.

APXlevél

	Idő	N	Subset for alpha = 0.05		
			1	2	3
Duncan[a]	0	3	,1575		
	1	3	,1634		
	3	3		,1989	
	7	3			,2361
	Sig.		,701	1,000	1,000

Means for groups in homogeneous subsets are displayed.

a. Uses Harmonic Mean Sample Size = 3,000.

GRlevél

	Idő	N	Subset for alpha = 0.05
			1
Duncan[a]	0	3	,004596
	1	3	,004905
	7	3	,005817
	3	3	,006187
	Sig.		,236

Means for groups in homogeneous subsets are displayed.

a. Uses Harmonic Mean Sample Size = 3,000.
