# Supplementary material for: Effect of cadmium stress on certain physiological parameters, antioxidative enzyme activities and biophoton emission of leaves in barley (Hordeum vulgare L.) seedlings
Source: PLoS One. 2020 Nov 3;15(11):e0240470. doi: 10.1371/journal.pone.0240470 (PMC7608874; doi:10.1371/journal.pone.0240470)
Supplement: S1 File — (ZIP) [file pone.0240470.s003.zip › stat result time-50 Cd SPAD leaf.pdf]

```
ONEWAY SPAD BY Idő
  /STATISTICS DESCRIPTIVES HOMOGENEITY
  /PLOT MEANS
  /MISSING ANALYSIS
  /POSTHOC=DUNCAN T2 ALPHA(0.05).
```

Oneway

[DataSet1] H:\Jócsák\01 Növényélettan\árpa vizsgálatok\PhD téma folytatása \SPAD\SPAD-two-way-anova.sav

Descriptives

SPAD

	N	Mean	Std. Deviation	Std. Error	95% Confidence Interval for Mean	
					Lower Bound	Upper Bound
0	100	28,0390	3,13310	,31331	27,4173	28,6607
1	100	28,5730	4,32983	,43298	27,7139	29,4321
3	100	21,0430	5,45472	,54547	19,9607	22,1253
7	100	20,0690	6,28634	,62863	18,8217	21,3163
Total	400	24,4310	6,28355	,31418	23,8134	25,0486

Descriptives

SPAD

	Minimum	Maximum
0	22,10	38,90
1	22,10	55,80
3	8,50	32,10
7	5,50	31,50
Total	5,50	55,80

Test of Homogeneity of Variances

SPAD

Levene Statistic	df1	df2	Sig.
18,810	3	396	,000

ANOVA

SPAD

	Sum of Squares	df	Mean Square	F	Sig.
Between Groups	6067,942	3	2022,647	82,695	,000
Within Groups	9685,755	396	24,459		
Total	15753,697	399			
