# Supplementary material for: Effect of cadmium stress on certain physiological parameters, antioxidative enzyme activities and biophoton emission of leaves in barley (Hordeum vulgare L.) seedlings
Source: PLoS One. 2020 Nov 3;15(11):e0240470. doi: 10.1371/journal.pone.0240470 (PMC7608874; doi:10.1371/journal.pone.0240470)
Supplement: S1 File — (ZIP) [file pone.0240470.s003.zip › stat result time-50 Cd SPAD leaf-2.pdf]

Post Hoc Tests

Multiple Comparisons

Dependent Variable: SPAD

	(I) Idő	(J) Idő	Mean Difference (I-J)	Std. Error	Sig.	95% Confidence Interval Lower Bound	Upper Bound
Tamhane	0	1	-,53400	,53445	,900	-1,9558	,8878
		3	6,99600*	,62905	,000	5,3199	8,6721
		7	7,97000*	,70238	,000	6,0965	9,8435
	1	0	,53400	,53445	,900	-,8878	1,9558
		3	7,53000*	,69643	,000	5,6782	9,3818
		7	8,50400*	,76332	,000	6,4728	10,5352
	3	0	-6,99600*	,62905	,000	-8,6721	-5,3199
		1	-7,53000*	,69643	,000	-9,3818	-5,6782
		7	,97400	,83230	,812	-1,2384	3,1864
	7	0	-7,97000*	,70238	,000	-9,8435	-6,0965
		1	-8,50400*	,76332	,000	-10,5352	-6,4728
		3	-,97400	,83230	,812	-3,1864	1,2384

*. The mean difference is significant at the 0.05 level.

Homogeneous Subsets

SPAD

	Idő	N	Subset for alpha = 0.05: 1	2
Duncan[a]	7	100	20,0690	
	3	100	21,0430	
	0	100		28,0390
	1	100		28,5730
	Sig.		,165	,446

Means for groups in homogeneous subsets are displayed.

a. Uses Harmonic Mean Sample Size = 100,000.

Means Plots
