# Supplementary material for: Effect of cadmium stress on certain physiological parameters, antioxidative enzyme activities and biophoton emission of leaves in barley (Hordeum vulgare L.) seedlings
Source: PLoS One. 2020 Nov 3;15(11):e0240470. doi: 10.1371/journal.pone.0240470 (PMC7608874; doi:10.1371/journal.pone.0240470)
Supplement: S1 File — (ZIP) [file pone.0240470.s003.zip › stat result time-50 Cd SPAD.pdf]

```
ONEWAY AA1 BY Idő
  /STATISTICS DESCRIPTIVES HOMOGENEITY
  /MISSING ANALYSIS
  /POSTHOC=DUNCAN T2 ALPHA(0.05).
```

Oneway

[DataSet2] H:\Jócsák\01 Növényélettan\árpa vizsgálatok\PhD téma folytatása \Visi É árpa c vit meghatározás\aszkorbinsav mg-g fr tömeg.sav

Descriptives

AA1

	N	Mean	Std. Deviation	Std. Error	95% Confidence Interval for Mean	
					Lower Bound	Upper Bound
1	2	,7870	,02036	,01440	,6040	,9700
3	2	,9262	,00304	,00215	,8988	,9535
7	2	1,2966	,22691	,16045	-,7422	3,3353
Total	6	1,0032	,25666	,10478	,7339	1,2726

Descriptives

AA1

	Minimum	Maximum
1	,77	,80
3	,92	,93
7	1,14	1,46
Total	,77	1,46

Test of Homogeneity of Variances

AA1

Levene Statistic	df1	df2	Sig.
.	2	.	.

ANOVA

AA1

	Sum of Squares	df	Mean Square	F	Sig.
Between Groups	,277	2	,139	8,017	,063
Within Groups	,052	3	,017		
Total	,329	5			

Post Hoc Tests

Multiple Comparisons

Dependent Variable: AA1

	(I) Idő	(J) Idő	Mean Difference (I-J)	Std. Error	Sig.	95% Confidence Interval	
						Lower Bound	Upper Bound
Tamhane	1	3	-,13915	,01456	,171	-,6116	,3333
		7	-,50955	,16109	,472	-6,2377	5,2186
	3	1	,13915	,01456	,171	-,3333	,6116
		7	-,37040	,16046	,595	-6,3875	5,6467
	7	1	,50955	,16109	,472	-5,2186	6,2377
		3	,37040	,16046	,595	-5,6467	6,3875

Homogeneous Subsets

AA1

	Idő	N	Subset for alpha = 0.05	
			1	2
Duncan[a]	1	2	,7870	
	3	2	,9262	,9262
	7	2		1,2966
	Sig.		,368	,067

Means for groups in homogeneous subsets are displayed.

a. Uses Harmonic Mean Sample Size = 2,000.
