# Supplementary material for: Effect of cadmium stress on certain physiological parameters, antioxidative enzyme activities and biophoton emission of leaves in barley (Hordeum vulgare L.) seedlings
Source: PLoS One. 2020 Nov 3;15(11):e0240470. doi: 10.1371/journal.pone.0240470 (PMC7608874; doi:10.1371/journal.pone.0240470)
Supplement: S1 File — (ZIP) [file pone.0240470.s003.zip › stat results AA leaf.pdf]

```
ONEWAY aszkorbinsavegynap aszkorbinsavháromnap aszkorbinsavhétnap BY Időke
zelés
  /STATISTICS DESCRIPTIVES HOMOGENEITY BROWNFORSYTHE WELCH
  /PLOT MEANS
  /MISSING ANALYSIS
  /POSTHOC=DUNCAN ALPHA(0.05).
```

Oneway

[DataSet0]

Descriptives

		N	Mean	Std. Deviation	Std. Error	95% Confidence ... Lower Bound
aszkorbinsavegynap	0	2	,008250	,0024749	,0017500	-,013986
	10	2	,008600	0E-7	0E-7	,008600
	50	2	,009350	,0002121	,0001500	,007444
	100	2	,011500	,0002828	,0002000	,008959
	300	2	,016900	,0002828	,0002000	,014359
	Total	10	,010920	,0034717	,0010979	,008436
aszkorbinsavháromnap	0	2	,011200	,0001414	,0001000	,009929
	10	2	,009800	,0008485	,0006000	,002176
	50	2	,012000	,0004243	,0003000	,008188
	100	2	,013550	,0000707	,0000500	,012915
	300	2	,014350	,0006364	,0004500	,008632
	Total	10	,012180	,0017580	,0005559	,010922
aszkorbinsavhétnap	0	2	,012900	,0053740	,0038000	-,035384
	10	2	,013700	,0046669	,0033000	-,028230
	50	2	,019200	,0029698	,0021000	-,007483
	100	2	,018950	,0033234	,0023500	-,010910
	300	2	,026200	,0035355	,0025000	-,005566
	Total	10	,018190	,0058768	,0018584	,013986

Descriptives

		95% Confidence ...		
		Upper Bound	Minimum	Maximum
aszkorbinsavegynap	0	,030486	,0065	,0100
	10	,008600	,0086	,0086
	50	,011256	,0092	,0095
	100	,014041	,0113	,0117
	300	,019441	,0167	,0171
	Total	,013404	,0065	,0171
aszkorbinsavháromnap	0	,012471	,0111	,0113
	10	,017424	,0092	,0104
	50	,015812	,0117	,0123
	100	,014185	,0135	,0136
	300	,020068	,0139	,0148
	Total	,013438	,0092	,0148
aszkorbinsavhétnap	0	,061184	,0091	,0167
	10	,055630	,0104	,0170
	50	,045883	,0171	,0213
	100	,048810	,0166	,0213
	300	,057966	,0237	,0287
	Total	,022394	,0091	,0287

Test of Homogeneity of Variances

	Levene Statistic	df1	df2	Sig.
aszkorbinsavegynap	.	4	.	.
aszkorbinsavháromnap	.	4	.	.
aszkorbinsavhétnap	.	4	.	.

ANOVA

		Sum of Squares	df	Mean Square	F
aszkorbinsavegynap	Between Groups	,000	4	,000	20,171
	Within Groups	,000	5	,000	
	Total	,000	9		
aszkorbinsavháromnap	Between Groups	,000	4	,000	24,893
	Within Groups	,000	5	,000	
	Total	,000	9		
aszkorbinsavhétnap	Between Groups	,000	4	,000	3,430
	Within Groups	,000	5	,000	
	Total	,000	9		

ANOVA

		Sig.
aszkorbinsavegynap	Between Groups	,003
	Within Groups	
	Total	
aszkorbinsavháromnap	Between Groups	,002
	Within Groups	
	Total	
aszkorbinsavhétnap	Between Groups	,104
	Within Groups	
	Total	

Robust Tests of Equality of Means[b]

		Statistic[a]	df1	df2	Sig.
aszkorbinsavegynap	Welch	.	.	.	.
	Brown-Forsythe	.	.	.	.
aszkorbinsavháromnap	Welch	64,489	4	2,203	,011
	Brown-Forsythe	24,893	4	2,473	,022
aszkorbinsavhétnap	Welch	1,847	4	2,476	,348
	Brown-Forsythe	3,430	4	4,141	,126

a. Asymptotically F distributed.

b. Robust tests of equality of means cannot be performed for aszkorbinsavegynap because at least one group has 0 variance.

Post Hoc Tests

Homogeneous Subsets

aszkorbinsavegynap

Duncan[a]

		Subset for alpha = 0.05		
Időkezelés	N	1	2	3
0	2	,008250		
10	2	,008600	,008600	
50	2	,009350	,009350	
100	2		,011500	
300	2			,016900
Sig.		,384	,054	1,000

Means for groups in homogeneous subsets are displayed.

a. Uses Harmonic Mean Sample Size = 2,000.

aszkorbinsavháromnap

Duncan[a]

Időkezelés	N	Subset for alpha = 0.05		
		1	2	3
10	2	,009800		
0	2		,011200	
50	2		,012000	
100	2			,013550
300	2			,014350
Sig.		1,000	,182	,182

Means for groups in homogeneous subsets are displayed.

a. Uses Harmonic Mean Sample Size = 2,000.

aszkorbinsavhétnap

Duncan[a]

Időkezelés	N	Subset for alpha = 0.05	
		1	2
0	2	,012900	
10	2	,013700	
100	2	,018950	,018950
50	2	,019200	,019200
300	2		,026200
Sig.		,195	,144

Means for groups in homogeneous subsets are displayed.

a. Uses Harmonic Mean Sample Size = 2,000.

Means Plots

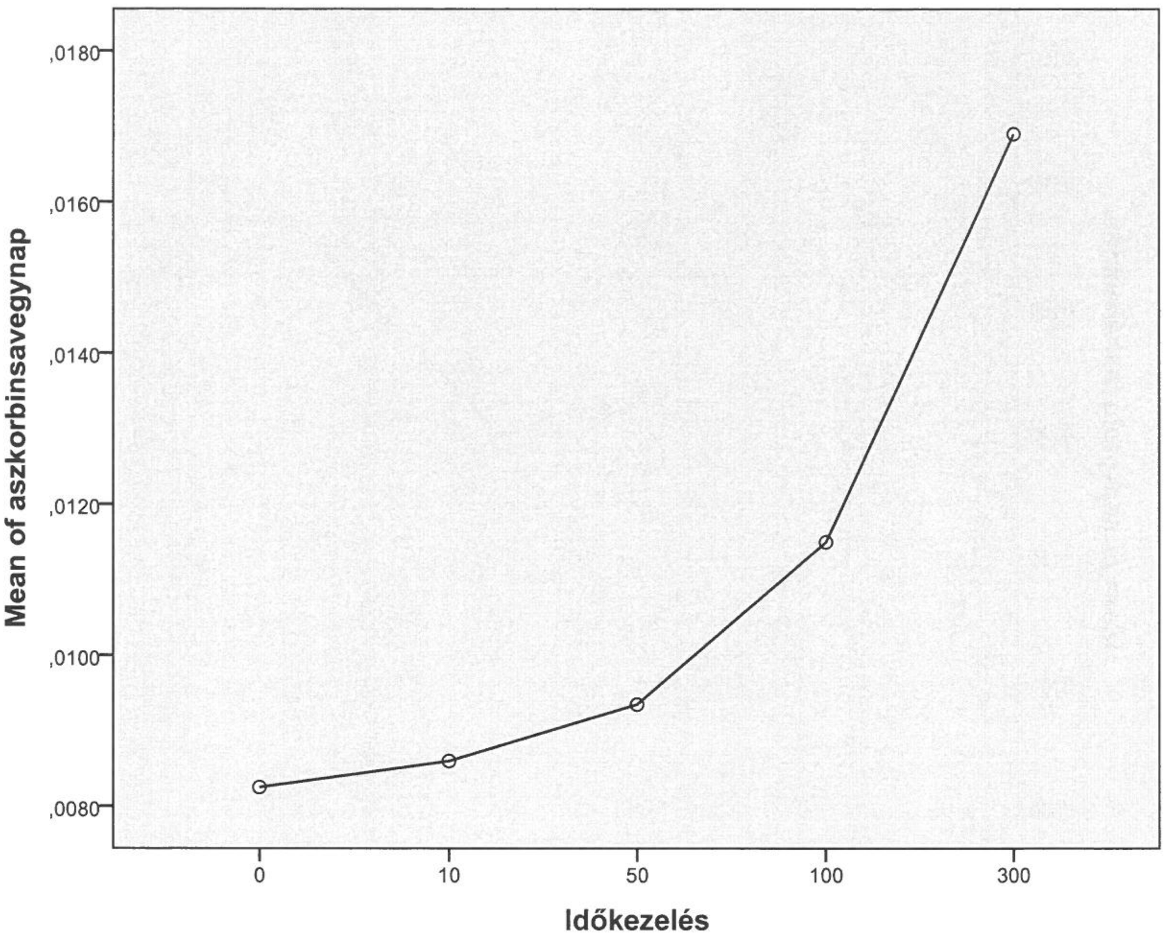
,0180
,0160
,0140
,0120
,0100
,0080
Mean of aszkorbinsavegynap
0
10
50
100
300
Időkezelés

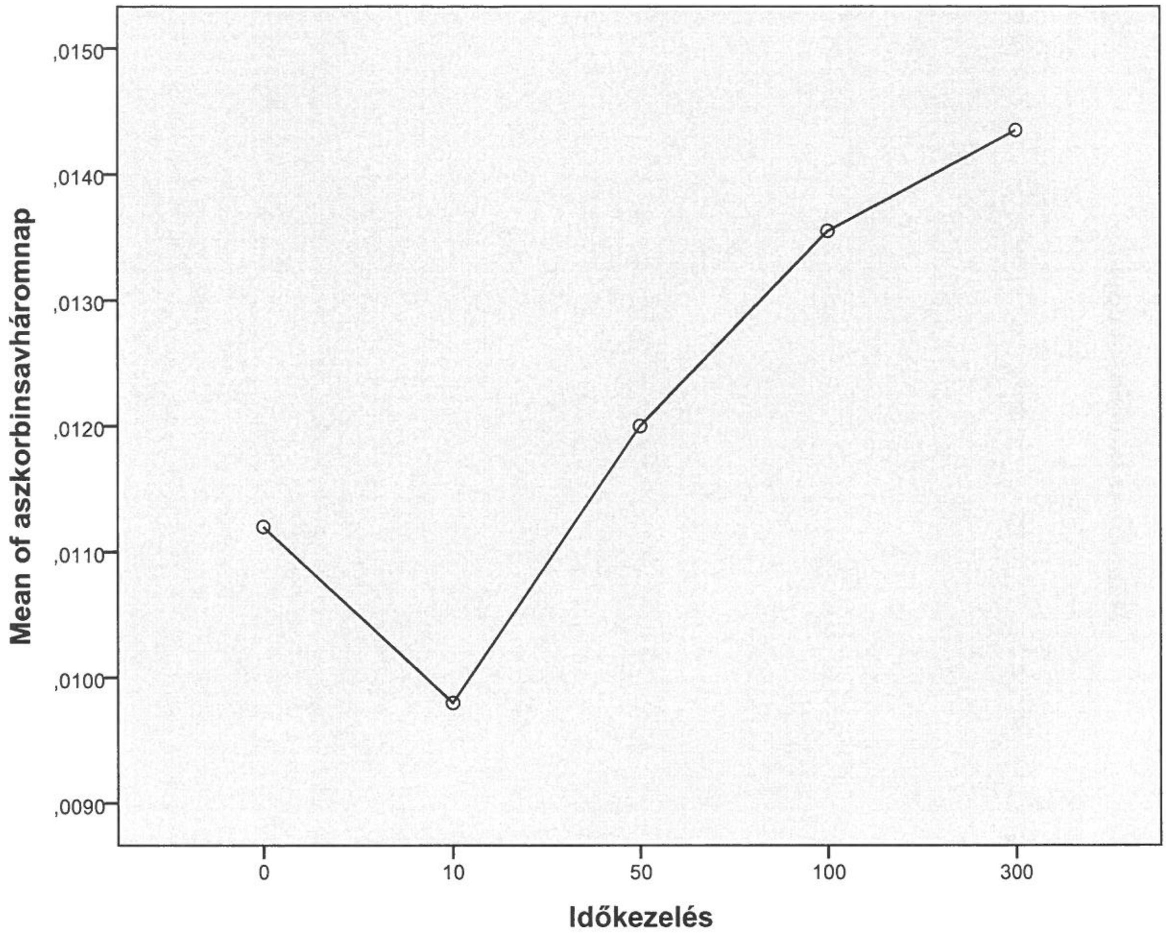
,0150
,0140
,0130
,0120
,0110
,0100
,0090
Mean of aszkorbinsavháromnap
0
10
50
100
300
Időkezelés

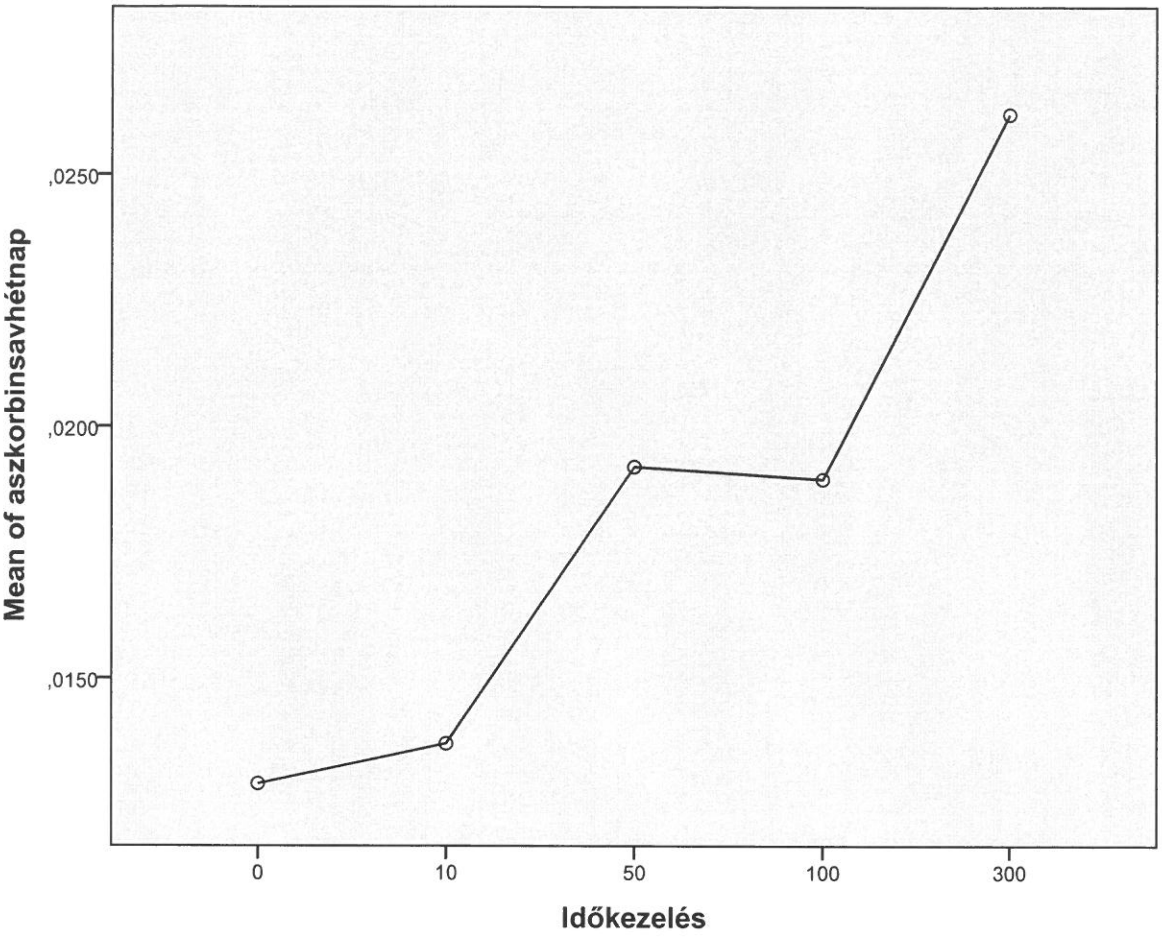


```
GET
  FILE='\\srv-fs01\home\jocsak.ildiko\Jócsák\01 Növényélettan\árpa vizsgál
atok\PhD téma folytatása\SPAD\SPAD.sav'.
DATASET NAME DataSet1 WINDOW=FRONT.
NEW FILE.
DATASET NAME DataSet2 WINDOW=FRONT.
```
