# Supplementary material for: Effect of cadmium stress on certain physiological parameters, antioxidative enzyme activities and biophoton emission of leaves in barley (Hordeum vulgare L.) seedlings
Source: PLoS One. 2020 Nov 3;15(11):e0240470. doi: 10.1371/journal.pone.0240470 (PMC7608874; doi:10.1371/journal.pone.0240470)
Supplement: S1 File — (ZIP) [file pone.0240470.s003.zip › stat results Cd - 1-3-7 day SPAD leaf.pdf]

```
ONEWAY SPAD0 SPAD1 SPAD3 SPAD7 BY Időkezelés
  /STATISTICS DESCRIPTIVES HOMOGENEITY BROWNFORSYTHE WELCH
  /PLOT MEANS
  /MISSING ANALYSIS
  /POSTHOC=DUNCAN ALPHA(0.05).
```

Oneway

[DataSet1] \\srv-fs01\home\jocsak.ildiko\Jócsák\01 Növényélettan\árpa vizs gálatok\SPAD\SPAD.sav

Warnings

There are fewer than two groups for dependent variable SPAD0. No statistics are computed.

Descriptives

						95% Confidence Interval for Mean	
		N	Mean	Std. Deviation	Std. Error	Lower Bound	Upper Bound
SPAD1	0	100	29,5343	3,47909	,34791	28,8440	30,2246
	10	100	28,7793	3,42957	,34296	28,0988	29,4598
	50	100	28,5450	4,25311	,42531	27,7011	29,3889
	100	100	27,5580	2,82057	,28206	26,9983	28,1177
	300	100	28,5220	4,32305	,43231	27,6642	29,3798
	Total	500	28,5877	3,74307	,16739	28,2588	28,9166
SPAD3	0	100	27,1590	3,84671	,38467	26,3957	27,9223
	10	100	24,9810	5,42502	,54250	23,9046	26,0574
	50	100	21,0590	5,40011	,54001	19,9875	22,1305
	100	100	20,5670	4,48614	,44861	19,6769	21,4571
	300	100	20,8960	4,87297	,48730	19,9291	21,8629
	Total	500	22,9324	5,50825	,24634	22,4484	23,4164
SPAD7	0	100	30,2190	4,70623	,47062	29,2852	31,1528
	10	100	26,3920	5,04022	,50402	25,3919	27,3921
	50	100	20,3950	6,31879	,63188	19,1412	21,6488
	100	100	17,0120	7,17588	,71759	15,5882	18,4358
	300	100	14,6160	7,35562	,73556	13,1565	16,0755
	Total	500	21,7268	8,48900	,37964	20,9809	22,4727

Descriptives

		Minimum	Maximum
SPAD1	0	22,10	38,90
	10	22,10	38,90
	50	22,10	55,80
	100	22,10	38,90
	300	22,10	55,80
	Total	22,10	55,80
SPAD3	0	19,10	36,60
	10	10,30	35,30
	50	8,50	32,10
	100	11,00	30,20
	300	5,60	28,90
	Total	5,60	36,60
SPAD7	0	12,10	37,90
	10	15,70	36,10
	50	5,50	31,50
	100	2,00	31,30
	300	1,60	28,30
	Total	1,60	37,90

Test of Homogeneity of Variances

	Levene Statistic	df1	df2	Sig.
SPAD1	,938	4	495	,442
SPAD3	3,814	4	495	,005
SPAD7	10,575	4	495	,000

ANOVA

		Sum of Squares	df	Mean Square	F	Sig.
SPAD1	Between Groups	199,918	4	49,980	3,643	,006
	Within Groups	6791,340	495	13,720		
	Total	6991,259	499			
SPAD3	Between Groups	3531,258	4	882,814	37,643	,000
	Within Groups	11608,797	495	23,452		
	Total	15140,055	499			
SPAD7	Between Groups	16844,806	4	4211,201	109,054	,000
	Within Groups	19114,715	495	38,616		
	Total	35959,521	499			

Robust Tests of Equality of Means

		Statistic[a]	df1	df2	Sig.
SPAD1	Welch	5,124	4	245,869	,001
	Brown-Forsythe	3,643	4	454,685	,006
SPAD3	Welch	46,847	4	246,349	,000
	Brown-Forsythe	37,643	4	468,686	,000
SPAD7	Welch	120,735	4	245,436	,000
	Brown-Forsythe	109,054	4	444,172	,000

a. Asymptotically F distributed.

Post Hoc Tests

Homogeneous Subsets

SPAD1

Duncan[a]

		Subset for alpha = 0.05	
Időkezelés	N	1	2
100	100	27,5580	
300	100	28,5220	28,5220
50	100	28,5450	28,5450
10	100		28,7793
0	100		29,5343
Sig.		,075	,078

Means for groups in homogeneous subsets are displayed.

a. Uses Harmonic Mean Sample Size = 100,000.

SPAD3

Duncan[a]

		Subset for alpha = 0.05		
Időkezelés	N	1	2	3
100	100	20,5670		
300	100	20,8960		
50	100	21,0590		
10	100		24,9810	
0	100			27,1590
Sig.		,503	1,000	1,000

Means for groups in homogeneous subsets are displayed.

a. Uses Harmonic Mean Sample Size = 100,000.

SPAD7

Duncan[a]

Időkezelés	N	Subset for alpha = 0.05				
		1	2	3	4	5
300	100	14,6160				
100	100		17,0120			
50	100			20,3950		
10	100				26,3920	
0	100					30,2190
Sig.		1,000	1,000	1,000	1,000	1,000

Means for groups in homogeneous subsets are displayed.

a. Uses Harmonic Mean Sample Size = 100,000.

Means Plots

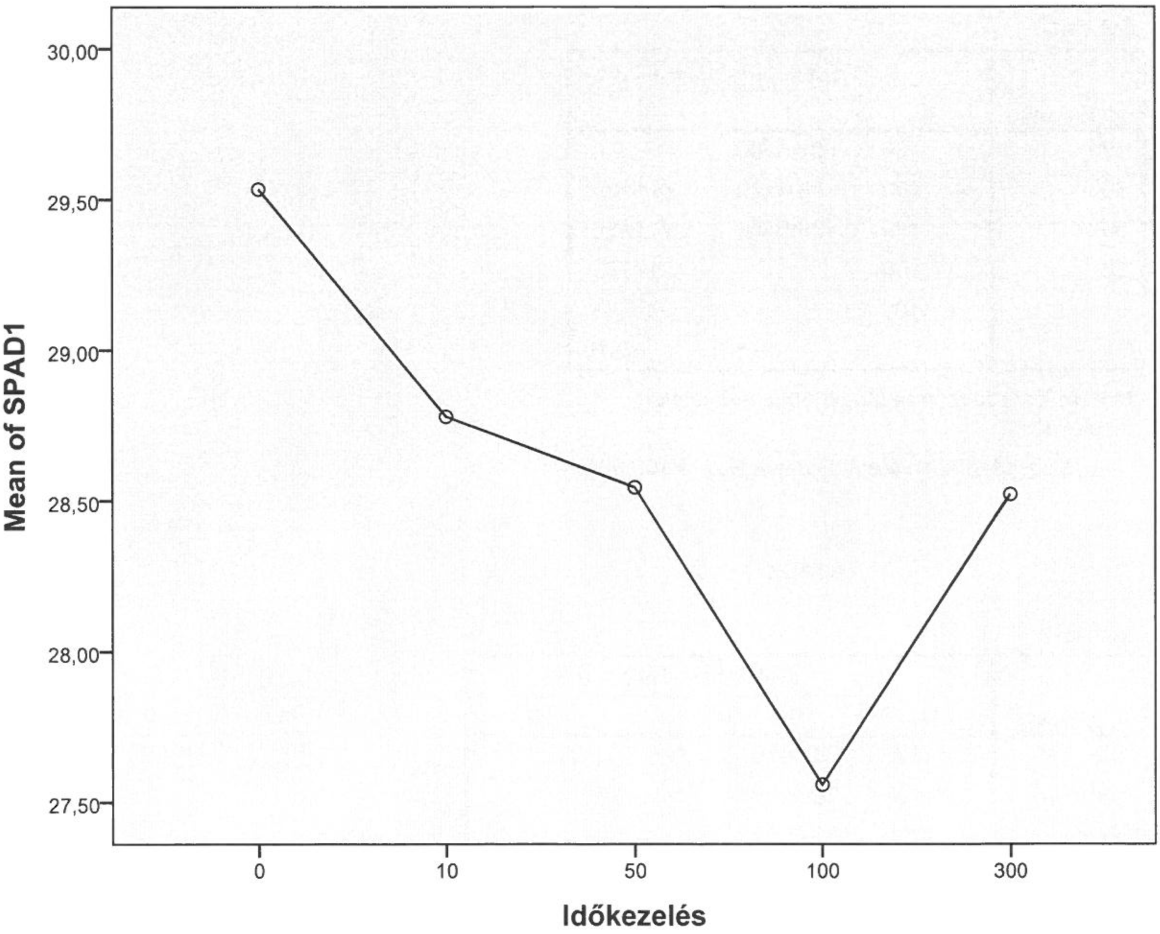

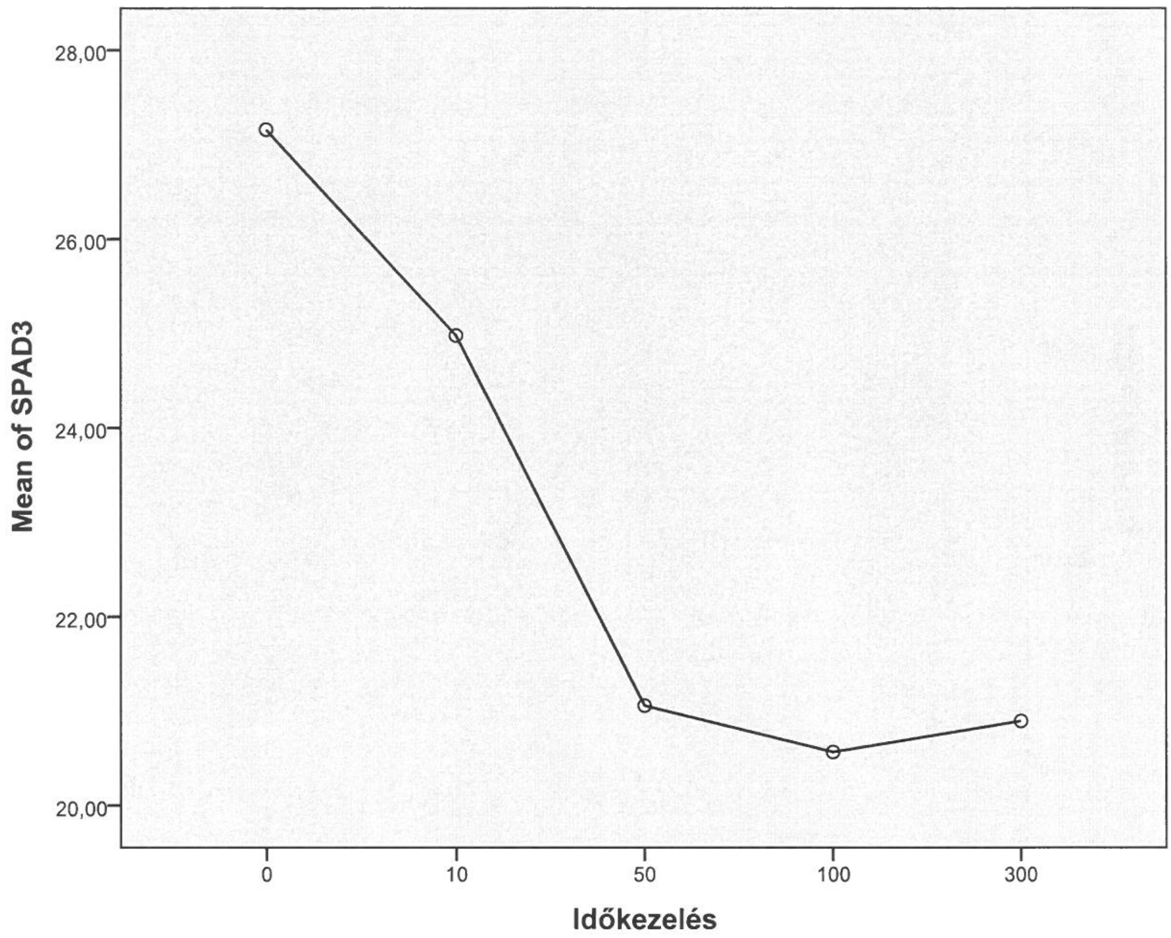
28,00
26,00
24,00
22,00
20,00
Mean of SPAD3
0
10
50
100
300
Időkezelés

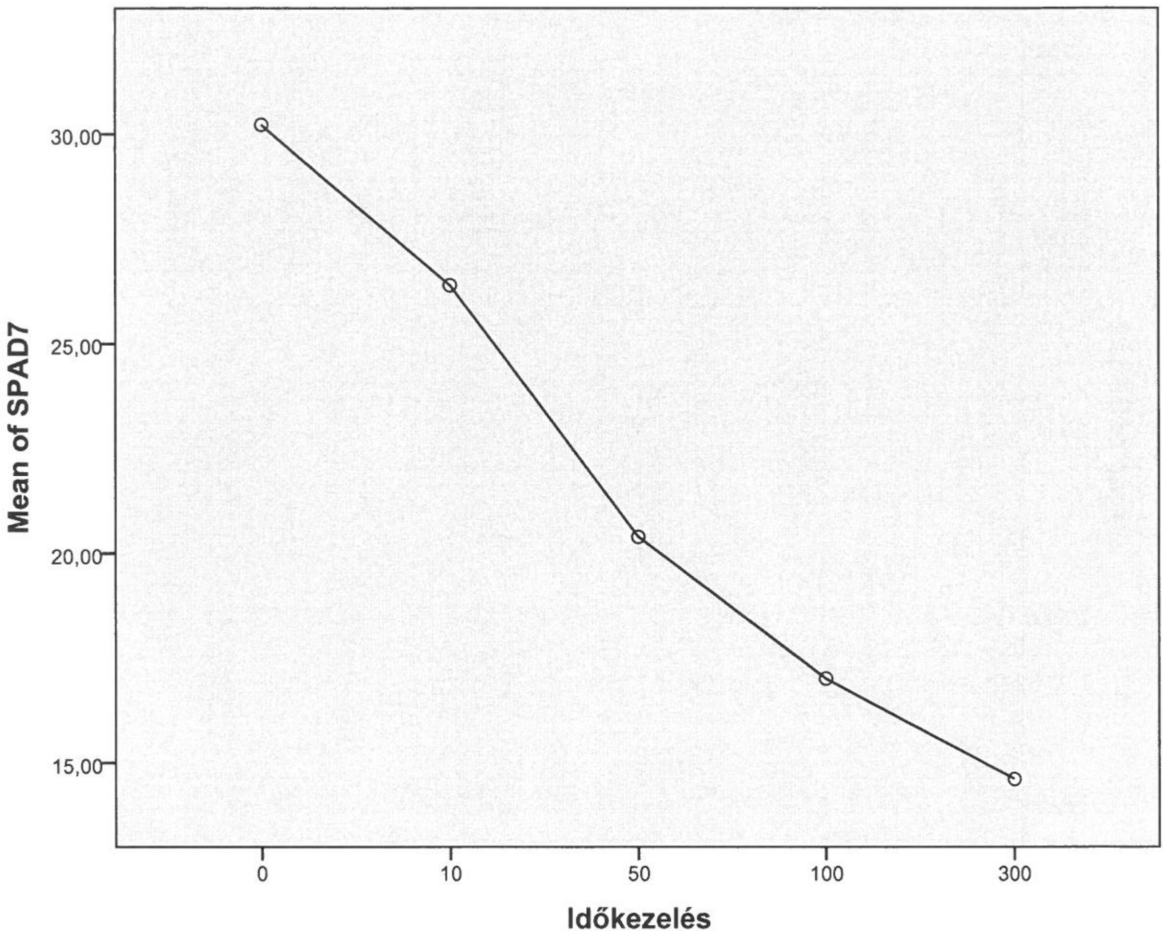
30,00
25,00
20,00
15,00
Mean of SPAD7
0
10
50
100
300
Időkezelés
