# Supplementary material for: Effect of cadmium stress on certain physiological parameters, antioxidative enzyme activities and biophoton emission of leaves in barley (Hordeum vulgare L.) seedlings
Source: PLoS One. 2020 Nov 3;15(11):e0240470. doi: 10.1371/journal.pone.0240470 (PMC7608874; doi:10.1371/journal.pone.0240470)
Supplement: S1 File — (ZIP) [file pone.0240470.s003.zip › stat results Cd - 7 day SPAD leaf.pdf]

```
ONEWAY SPAD BY Kezelés
  /STATISTICS DESCRIPTIVES HOMOGENEITY
  /PLOT MEANS
  /MISSING ANALYSIS
  /POSTHOC=DUNCAN T2 ALPHA(0.05).
```

Oneway

[DataSet1] H:\Jócsák\01 Növényélettan\árpa vizsgálatok\PhD téma folytatása \SPAD\SPAD-two-way-anova.sav

Descriptives

SPAD

	N	Mean	Std. Deviation	Std. Error	95% Confidence Interval for Mean	
					Lower Bound	Upper Bound
0	100	29,9740	4,90198	,49020	29,0013	30,9467
10	100	26,3920	5,04022	,50402	25,3919	27,3921
50	100	20,3950	6,31879	,63188	19,1412	21,6488
100	100	17,0120	7,17588	,71759	15,5882	18,4358
300	100	13,9730	6,86247	,68625	12,6113	15,3347
Total	500	21,5492	8,49159	,37976	20,8031	22,2953

Descriptives

SPAD

	Minimum	Maximum
0	12,10	37,90
10	15,70	36,10
50	5,50	31,50
100	2,00	31,30
300	1,60	28,30
Total	1,60	37,90

Test of Homogeneity of Variances

SPAD

Levene Statistic	df1	df2	Sig.
6,915	4	495	,000

ANOVA

SPAD

	Sum of Squares	df	Mean Square	F	Sig.
Between Groups	17374,713	4	4343,678	115,556	,000
Within Groups	18606,756	495	37,589		
Total	35981,470	499			

Post Hoc Tests

Multiple Comparisons

Dependent Variable: SPAD

	(I) Kezelés	(J) Kezelés	Mean Difference (I-J)	Std. Error	Sig.	95% ... Lower Bound
Tamhane	0	10	3,58200*	,70309	,000	1,5914
		50	9,57900*	,79973	,000	7,3132
		100	12,96200*	,86904	,000	10,4979
		300	16,00100*	,84334	,000	13,6105
	10	0	-3,58200*	,70309	,000	-5,5726
		50	5,99700*	,80828	,000	3,7073
		100	9,38000*	,87691	,000	6,8941
		300	12,41900*	,85145	,000	10,0059
	50	0	-9,57900*	,79973	,000	-11,8448
		10	-5,99700*	,80828	,000	-8,2867
		100	3,38300*	,95614	,005	,6755
		300	6,42200*	,93285	,000	3,7807
	100	0	-12,96200*	,86904	,000	-15,4261
		10	-9,38000*	,87691	,000	-11,8659
		50	-3,38300*	,95614	,005	-6,0905
		300	3,03900*	,99291	,025	,2278
	300	0	-16,00100*	,84334	,000	-18,3915
		10	-12,41900*	,85145	,000	-14,8321
		50	-6,42200*	,93285	,000	-9,0633
		100	-3,03900*	,99291	,025	-5,8502

Multiple Comparisons

Dependent Variable: SPAD

	(I) Kezelés	(J) Kezelés	95% ... Upper Bound
Tamhane	0	10	5,5726
		50	11,8448
		100	15,4261
		300	18,3915
	10	0	-1,5914
		50	8,2867
		100	11,8659
		300	14,8321
	50	0	-7,3132
		10	-3,7073
		100	6,0905
		300	9,0633
	100	0	-10,4979
		10	-6,8941
		50	-,6755
		300	5,8502
	300	0	-13,6105
		10	-10,0059
		50	-3,7807
		100	-,2278

*. The mean difference is significant at the 0.05 level.

Homogeneous Subsets

SPAD

	Kezelés	N	Subset for alpha = 0.05 1	2	3	4	5
Duncan[a]	300	100	13,9730				
	100	100		17,0120			
	50	100			20,3950		
	10	100				26,3920	
	0	100					29,9740
	Sig.		1,000	1,000	1,000	1,000	1,000

Means for groups in homogeneous subsets are displayed.

a. Uses Harmonic Mean Sample Size = 100,000.

Means Plots

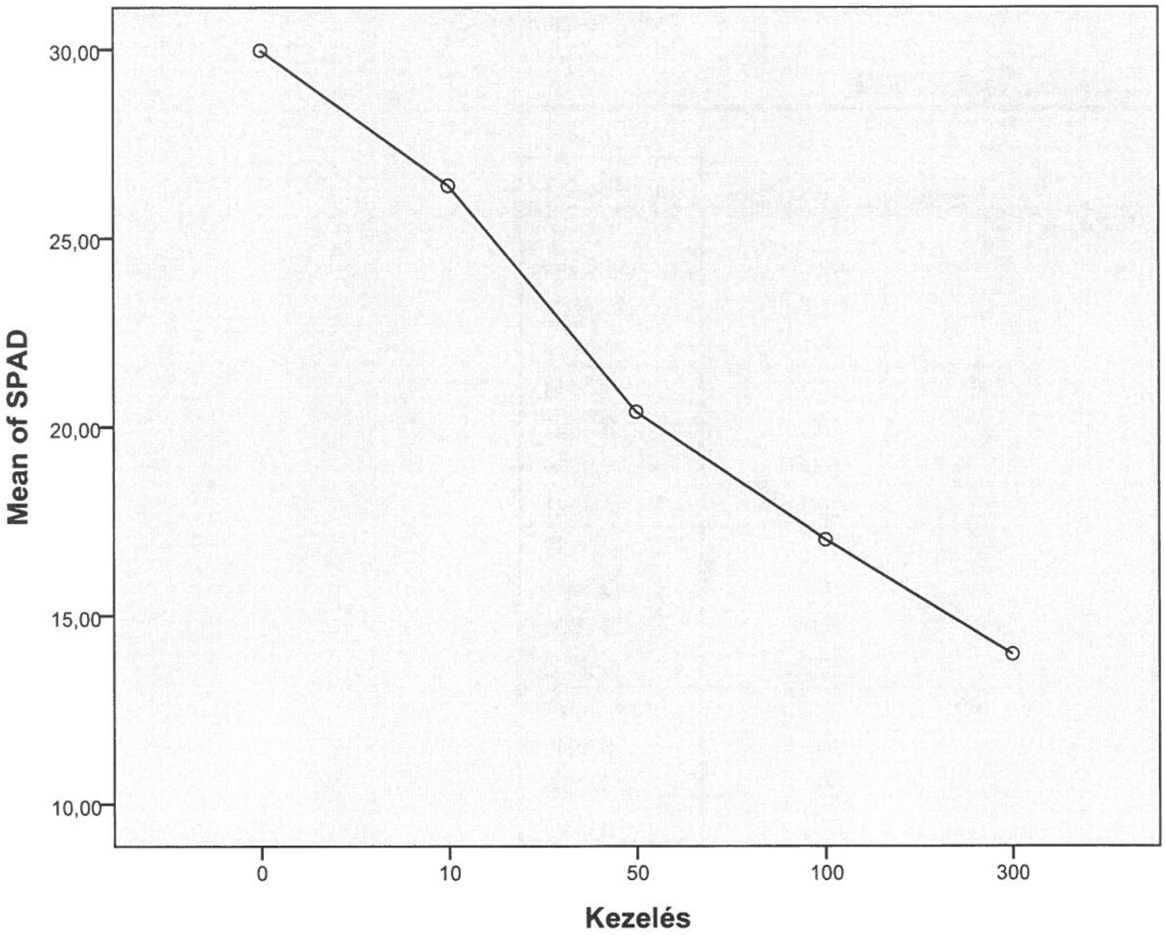
30,00
25,00
20,00
15,00
10,00
Mean of SPAD
0
10
50
100
300
Kezelés
