# Supplementary material for: Effect of cadmium stress on certain physiological parameters, antioxidative enzyme activities and biophoton emission of leaves in barley (Hordeum vulgare L.) seedlings
Source: PLoS One. 2020 Nov 3;15(11):e0240470. doi: 10.1371/journal.pone.0240470 (PMC7608874; doi:10.1371/journal.pone.0240470)
Supplement: S1 File — (ZIP) [file pone.0240470.s003.zip › stat results Cd -0 day Cd content leaf.pdf]

```
ONEWAY Kadmiumtartlev BY Kadmium
  /STATISTICS DESCRIPTIVES HOMOGENEITY
  /PLOT MEANS
  /MISSING ANALYSIS
  /POSTHOC=DUNCAN T2 ALPHA(0.05).
```

Oneway

[DataSet1] H:\Jócsák\01 Növényélettan\árpa vizsgálatok\PhD téma folytatása \MGHgyökér_1.sav

Descriptives

Kadmiumtartlev

	N	Mean	Std. Deviation	Std. Error	95% Confidence Interval for Mean	
					Lower Bound	Upper Bound
0	3	,5367	,03055	,01764	,4608	,6126
10	3	,5367	,03055	,01764	,4608	,6126
50	3	,5367	,03055	,01764	,4608	,6126
100	3	,5367	,03055	,01764	,4608	,6126
300	3	,5367	,03055	,01764	,4608	,6126
Total	15	,5367	,02582	,00667	,5224	,5510

Descriptives

Kadmiumtartlev

	Minimum	Maximum
0	,51	,57
10	,51	,57
50	,51	,57
100	,51	,57
300	,51	,57
Total	,51	,57

Test of Homogeneity of Variances

Kadmiumtartlev

Levene Statistic	df1	df2	Sig.
,000	4	10	1,000

ANOVA

Kadmiumtartlev

	Sum of Squares	df	Mean Square	F	Sig.
Between Groups	,000	4	,000	,000	1,000
Within Groups	,009	10	,001		
Total	,009	14			

Post Hoc Tests

Multiple Comparisons

Dependent Variable: Kadmiumtartlev

	(I) Kadmium	(J) Kadmium	Mean Difference (I-J)	Std. Error	Sig.	95% ...
						Lower Bound
Tamhane	0	10	,00000	,02494	1,000	-,1387
		50	,00000	,02494	1,000	-,1387
		100	,00000	,02494	1,000	-,1387
		300	,00000	,02494	1,000	-,1387
	10	0	,00000	,02494	1,000	-,1387
		50	,00000	,02494	1,000	-,1387
		100	,00000	,02494	1,000	-,1387
		300	,00000	,02494	1,000	-,1387
	50	0	,00000	,02494	1,000	-,1387
		10	,00000	,02494	1,000	-,1387
		100	,00000	,02494	1,000	-,1387
		300	,00000	,02494	1,000	-,1387
	100	0	,00000	,02494	1,000	-,1387
		10	,00000	,02494	1,000	-,1387
		50	,00000	,02494	1,000	-,1387
		300	,00000	,02494	1,000	-,1387
	300	0	,00000	,02494	1,000	-,1387
		10	,00000	,02494	1,000	-,1387
		50	,00000	,02494	1,000	-,1387
		100	,00000	,02494	1,000	-,1387

Multiple Comparisons

Dependent Variable: Kadmiumtartlev

	(I) Kadmium	(J) Kadmium	95% ... Upper Bound
Tamhane	0	10	,1387
		50	,1387
		100	,1387
		300	,1387
	10	0	,1387
		50	,1387
		100	,1387
		300	,1387
	50	0	,1387
		10	,1387
		100	,1387
		300	,1387
	100	0	,1387
		10	,1387
		50	,1387
		300	,1387
	300	0	,1387
		10	,1387
		50	,1387
		100	,1387

Homogeneous Subsets

Kadmiumtartlev

	Kadmium	N	Subset for alpha = 0.05
			1
Duncan[a]	0	3	,5367
	10	3	,5367
	50	3	,5367
	100	3	,5367
	300	3	,5367
	Sig.		1,000

Means for groups in homogeneous subsets are displayed.

a. Uses Harmonic Mean Sample Size = 3,000.

Means Plots

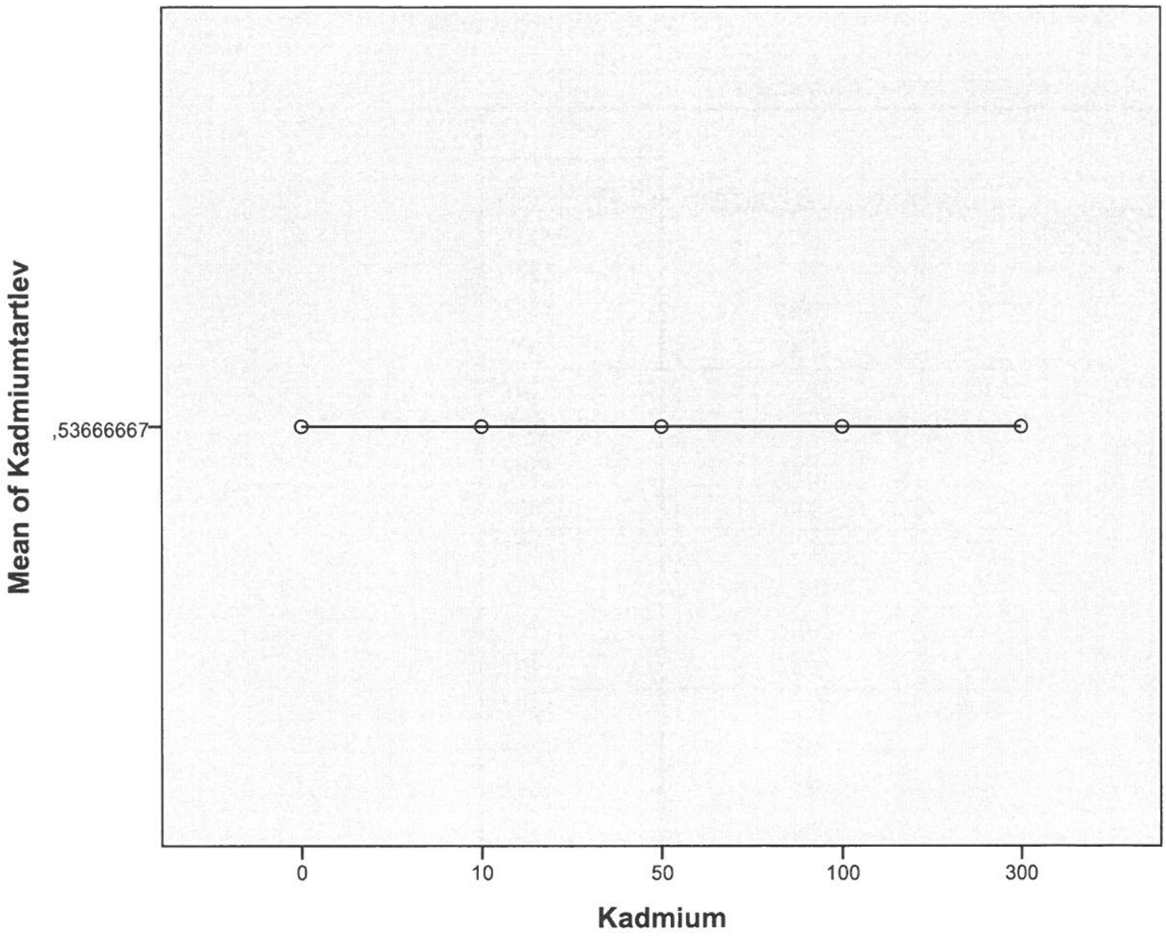
Mean of Kadmiumtartlev
,53666667
0
10
50
100
300
Kadmium
