# Supplementary material for: Effect of cadmium stress on certain physiological parameters, antioxidative enzyme activities and biophoton emission of leaves in barley (Hordeum vulgare L.) seedlings
Source: PLoS One. 2020 Nov 3;15(11):e0240470. doi: 10.1371/journal.pone.0240470 (PMC7608874; doi:10.1371/journal.pone.0240470)
Supplement: S1 File — (ZIP) [file pone.0240470.s003.zip › stat results Cd -1 day Cd content leaf.pdf]

```
ONEWAY Kadmiumtartlev BY Kadmium
  /STATISTICS DESCRIPTIVES HOMOGENEITY
  /PLOT MEANS
  /MISSING ANALYSIS
  /POSTHOC=DUNCAN T2 ALPHA(0.05).
```

Oneway

```
[DataSet1] H:\Jócsák\01 Növényélettan\árpa vizsgálatok\PhD téma folytatása
\MGHgyökér_1.sav
```

Descriptives

Kadmiumtartlev

	N	Mean	Std. Deviation	Std. Error	95% Confidence Interval for Mean	
					Lower Bound	Upper Bound
0	3	2,0367	,23159	,13371	1,4614	2,6120
10	3	10,4633	,76009	,43884	8,5752	12,3515
50	3	12,8633	,65287	,37693	11,2415	14,4851
100	3	27,1567	2,44876	1,41379	21,0736	33,2397
300	3	49,6633	3,04894	1,76031	42,0893	57,2373
Total	15	20,4367	17,35536	4,48113	10,8256	30,0477

Descriptives

Kadmiumtartlev

	Minimum	Maximum
0	1,78	2,23
10	9,71	11,23
50	12,16	13,45
100	24,79	29,68
300	47,56	53,16
Total	1,78	53,16

Test of Homogeneity of Variances

Kadmiumtartlev

Levene Statistic	df1	df2	Sig.
4,046	4	10	,033

ANOVA

Kadmiumtartlev

	Sum of Squares	df	Mean Square	F	Sig.
Between Groups	4184,218	4	1046,054	319,893	,000
Within Groups	32,700	10	3,270		
Total	4216,918	14			

Post Hoc Tests

Multiple Comparisons

Dependent Variable: Kadmiumtartlev

	(I) Kadmium	(J) Kadmium	Mean Difference (I-J)	Std. Error	Sig.	95% ...
						Lower Bound
Tamhane	0	10	-8,42667*	,45875	,013	-13,1383
		50	-10,82667*	,39994	,004	-14,6121
		100	-25,12000*	1,42010	,029	-44,2149
		300	-47,62667*	1,76538	,013	-71,6581
	10	0	8,42667*	,45875	,013	3,7150
		50	-2,40000	,57849	,140	-5,6782
		100	-16,69333*	1,48033	,039	-31,7571
		300	-39,20000*	1,81418	,012	-59,5303
	50	0	10,82667*	,39994	,004	7,0413
		10	2,40000	,57849	,140	-,8782
		100	-14,29333	1,46318	,064	-30,2598
		300	-36,80000*	1,80021	,015	-58,0358
	100	0	25,12000*	1,42010	,029	6,0251
		10	16,69333*	1,48033	,039	1,6296
		50	14,29333	1,46318	,064	-1,6731
		300	-22,50667*	2,25776	,007	-35,5544
	300	0	47,62667*	1,76538	,013	23,5952
		10	39,20000*	1,81418	,012	18,8697
		50	36,80000*	1,80021	,015	15,5642
		100	22,50667*	2,25776	,007	9,4589

Multiple Comparisons

Dependent Variable: Kadmiumtartlev

	(I) Kadmium	(J) Kadmium	95% ... Upper Bound
Tamhane	0	10	-3,7150
		50	-7,0413
		100	-6,0251
		300	-23,5952
	10	0	13,1383
		50	,8782
		100	-1,6296
		300	-18,8697
	50	0	14,6121
		10	5,6782
		100	1,6731
		300	-15,5642
	100	0	44,2149
		10	31,7571
		50	30,2598
		300	-9,4589
	300	0	71,6581
		10	59,5303
		50	58,0358
		100	35,5544

*. The mean difference is significant at the 0.05 level.

Homogeneous Subsets

Kadmiumtartlev

	Kadmium	N	Subset for alpha = 0.05 1	2	3	4
Duncan[a]	0	3	2,0367			
	10	3		10,4633		
	50	3		12,8633		
	100	3			27,1567	
	300	3				49,6633
	Sig.		1,000	,135	1,000	1,000

Means for groups in homogeneous subsets are displayed.

a. Uses Harmonic Mean Sample Size = 3,000.

Means Plots

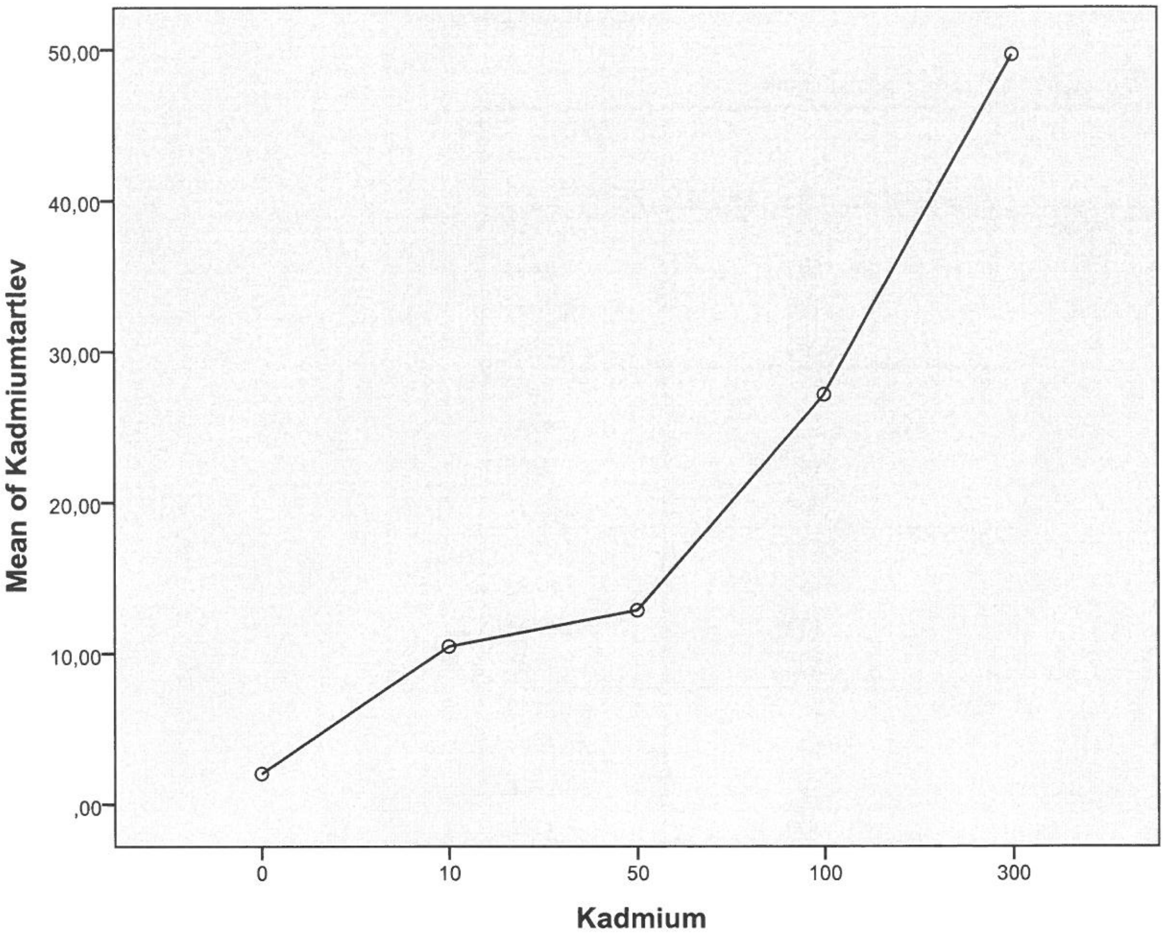
50,00
40,00
30,00
20,00
10,00
,00
Mean of Kadmiumtartlev
0
10
50
100
300
Kadmium
