# Supplementary material for: Effect of cadmium stress on certain physiological parameters, antioxidative enzyme activities and biophoton emission of leaves in barley (Hordeum vulgare L.) seedlings
Source: PLoS One. 2020 Nov 3;15(11):e0240470. doi: 10.1371/journal.pone.0240470 (PMC7608874; doi:10.1371/journal.pone.0240470)
Supplement: S1 File — (ZIP) [file pone.0240470.s003.zip › stat results Cd -3 day Cd content leaf.pdf]

```
ONEWAY Kadmiumtartlev BY Kadmium
  /STATISTICS DESCRIPTIVES HOMOGENEITY
  /PLOT MEANS
  /MISSING ANALYSIS
  /POSTHOC=DUNCAN T2 ALPHA(0.05).
```

Oneway

```
[DataSet1] H:\Jócsák\01 Növényélettan\árpa vizsgálatok\PhD téma folytatása
\MGHgyökér_1.sav
```

Descriptives

Kadmiumtartlev

	N	Mean	Std. Deviation	Std. Error	95% Confidence Interval for Mean	
					Lower Bound	Upper Bound
0	3	1,3267	,09074	,05239	1,1013	1,5521
10	3	7,8367	,68017	,39270	6,1470	9,5263
50	3	39,3700	3,64600	2,10502	30,3128	48,4272
100	3	61,0033	3,51654	2,03027	52,2678	69,7389
300	3	105,1067	10,39908	6,00391	79,2739	130,9394
Total	15	42,9287	39,43480	10,18202	21,0904	64,7669

Descriptives

Kadmiumtartlev

	Minimum	Maximum
0	1,23	1,41
10	7,21	8,56
50	36,56	43,49
100	56,98	63,49
300	95,14	115,89
Total	1,23	115,89

Test of Homogeneity of Variances

Kadmiumtartlev

Levene Statistic	df1	df2	Sig.
3,499	4	10	,049

ANOVA

Kadmiumtartlev

	Sum of Squares	df	Mean Square	F	Sig.
Between Groups	21502,909	4	5375,727	200,182	,000
Within Groups	268,542	10	26,854		
Total	21771,451	14			

Post Hoc Tests

Multiple Comparisons

Dependent Variable: Kadmiumtartlev

	(I) Kadmium	(J) Kadmium	Mean Difference (I-J)	Std. Error	Sig.	95% ...
						Lower Bound
Tamhane	0	10	-6,51000*	,39618	,031	-11,6620
		50	-38,04333*	2,10567	,030	-67,2962
		100	-59,67667*	2,03095	,011	-87,8863
		300	-103,78000*	6,00414	,033	-187,3736
	10	0	6,51000*	,39618	,031	1,3580
		50	-31,53333*	2,14133	,034	-57,7440
		100	-53,16667*	2,06790	,010	-78,2544
		300	-97,27000*	6,01674	,036	-179,6537
	50	0	38,04333*	2,10567	,030	8,7905
		10	31,53333*	2,14133	,034	5,3227
		100	-21,63333*	2,92457	,018	-37,9176
		300	-65,73667*	6,36224	,041	-126,3612
	100	0	59,67667*	2,03095	,011	31,4671
		10	53,16667*	2,06790	,010	28,0789
		50	21,63333*	2,92457	,018	5,3491
		300	-44,10333	6,33790	,108	-105,7396
	300	0	103,78000*	6,00414	,033	20,1864
		10	97,27000*	6,01674	,036	14,8863
		50	65,73667*	6,36224	,041	5,1121
		100	44,10333	6,33790	,108	-17,5330

Multiple Comparisons

Dependent Variable: Kadmiumtartlev

	(I) Kadmium	(J) Kadmium	95% ... Upper Bound
Tamhane	0	10	-1,3580
		50	-8,7905
		100	-31,4671
		300	-20,1864
	10	0	11,6620
		50	-5,3227
		100	-28,0789
		300	-14,8863
	50	0	67,2962
		10	57,7440
		100	-5,3491
		300	-5,1121
	100	0	87,8863
		10	78,2544
		50	37,9176
		300	17,5330
	300	0	187,3736
		10	179,6537
		50	126,3612
		100	105,7396

*. The mean difference is significant at the 0.05 level.

Homogeneous Subsets

Kadmiumtartlev

	Kadmium	N	Subset for alpha = 0.05 1	2	3	4
Duncan[a]	0	3	1,3267			
	10	3	7,8367			
	50	3		39,3700		
	100	3			61,0033	
	300	3				105,1067
	Sig.		,155	1,000	1,000	1,000

Means for groups in homogeneous subsets are displayed.

a. Uses Harmonic Mean Sample Size = 3,000.

Means Plots

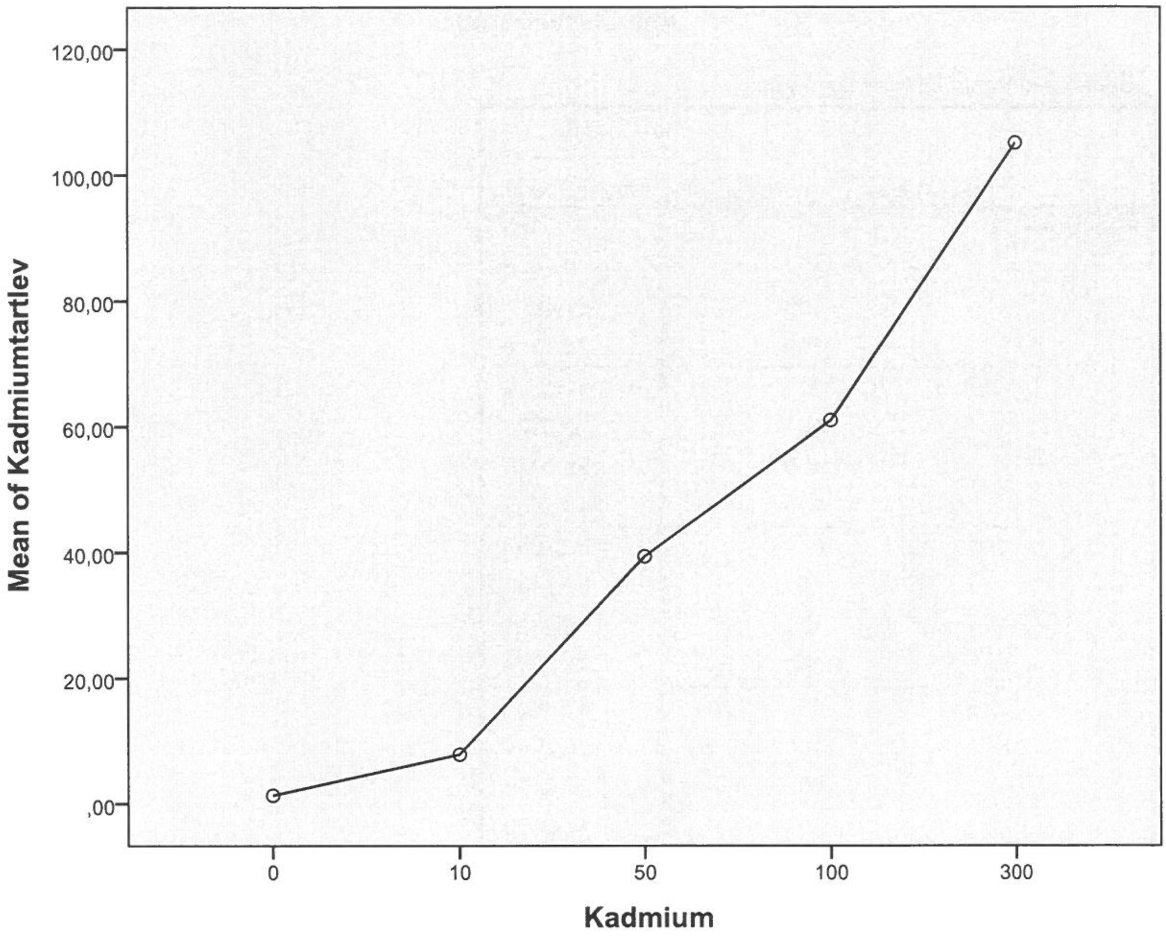
120,00
100,00
80,00
60,00
40,00
20,00
,00
Mean of Kadmiumtartlev
0
10
50
100
300
Kadmium
