# Supplementary material for: Effect of cadmium stress on certain physiological parameters, antioxidative enzyme activities and biophoton emission of leaves in barley (Hordeum vulgare L.) seedlings
Source: PLoS One. 2020 Nov 3;15(11):e0240470. doi: 10.1371/journal.pone.0240470 (PMC7608874; doi:10.1371/journal.pone.0240470)
Supplement: S1 File — (ZIP) [file pone.0240470.s003.zip › stat results Cd -7 day Cd content leaf.pdf]

```
ONEWAY Kadmiumtartlev BY Kadmium
  /STATISTICS DESCRIPTIVES HOMOGENEITY
  /PLOT MEANS
  /MISSING ANALYSIS
  /POSTHOC=DUNCAN T2 ALPHA(0.05).
```

Oneway

[DataSet1] H:\Jócsák\01 Növényélettan\árpa vizsgálatok\PhD téma folytatása \MGHgyökér_1.sav

Descriptives

Kadmiumtartlev

	N	Mean	Std. Deviation	Std. Error	95% Confidence Interval for Mean Lower Bound	95% Confidence Interval for Mean Upper Bound
0	3	1,7200	,25239	,14572	1,0930	2,3470
10	3	8,7400	,82177	,47445	6,6986	10,7814
50	3	49,3667	7,59485	4,38489	30,5000	68,2333
100	3	80,2033	7,93224	4,57968	60,4986	99,9081
300	3	143,7467	13,75321	7,94042	109,5818	177,9115
Total	15	56,7553	54,20842	13,99655	26,7357	86,7750

Descriptives

Kadmiumtartlev

	Minimum	Maximum
0	1,49	1,99
10	8,01	9,63
50	41,62	56,80
100	72,96	88,68
300	130,53	157,98
Total	1,49	157,98

Test of Homogeneity of Variances

Kadmiumtartlev

Levene Statistic	df1	df2	Sig.
2,595	4	10	,101

ANOVA

Kadmiumtartlev

	Sum of Squares	df	Mean Square	F	Sig.
Between Groups	40518,760	4	10129,690	163,123	,000
Within Groups	620,984	10	62,098		
Total	41139,744	14			

Post Hoc Tests

Multiple Comparisons

Dependent Variable: Kadmiumtartlev

	(I) Kadmium	(J) Kadmium	Mean Difference (I-J)	Std. Error	Sig.	95% ...
						Lower Bound
Tamhane	0	10	-7,02000*	,49632	,024	-12,0974
		50	-47,64667	4,38731	,080	-108,4792
		100	-78,48333*	4,58200	,033	-142,0386
		300	-142,02667*	7,94176	,031	-252,4818
	10	0	7,02000*	,49632	,024	1,9426
		50	-40,62667	4,41048	,103	-99,3071
		100	-71,46333*	4,60419	,037	-132,9489
		300	-135,00667*	7,95458	,033	-244,2281
	50	0	47,64667	4,38731	,080	-13,1858
		10	40,62667	4,41048	,103	-18,0537
		100	-30,83667	6,34040	,080	-66,1573
		300	-94,38000*	9,07070	,016	-158,5666
	100	0	78,48333*	4,58200	,033	14,9281
		10	71,46333*	4,60419	,037	9,9778
		50	30,83667	6,34040	,080	-4,4839
		300	-63,54333*	9,16645	,049	-126,5634
	300	0	142,02667*	7,94176	,031	31,5716
		10	135,00667*	7,95458	,033	25,7853
		50	94,38000*	9,07070	,016	30,1934
		100	63,54333*	9,16645	,049	,5233

Multiple Comparisons

Dependent Variable: Kadmiumtartlev

	(I) Kadmium	(J) Kadmium	95% ... Upper Bound
Tamhane	0	10	-1,9426
		50	13,1858
		100	-14,9281
		300	-31,5716
	10	0	12,0974
		50	18,0537
		100	-9,9778
		300	-25,7853
	50	0	108,4792
		10	99,3071
		100	4,4839
		300	-30,1934
	100	0	142,0386
		10	132,9489
		50	66,1573
		300	-,5233
	300	0	252,4818
		10	244,2281
		50	158,5666
		100	126,5634

*. The mean difference is significant at the 0.05 level.

Homogeneous Subsets

Kadmiumtartlev

	Kadmium	N	Subset for alpha = 0.05 1	2	3	4
Duncan[a]	0	3	1,7200			
	10	3	8,7400			
	50	3		49,3667		
	100	3			80,2033	
	300	3				143,7467
	Sig.		,301	1,000	1,000	1,000

Means for groups in homogeneous subsets are displayed.

a. Uses Harmonic Mean Sample Size = 3,000.

Means Plots

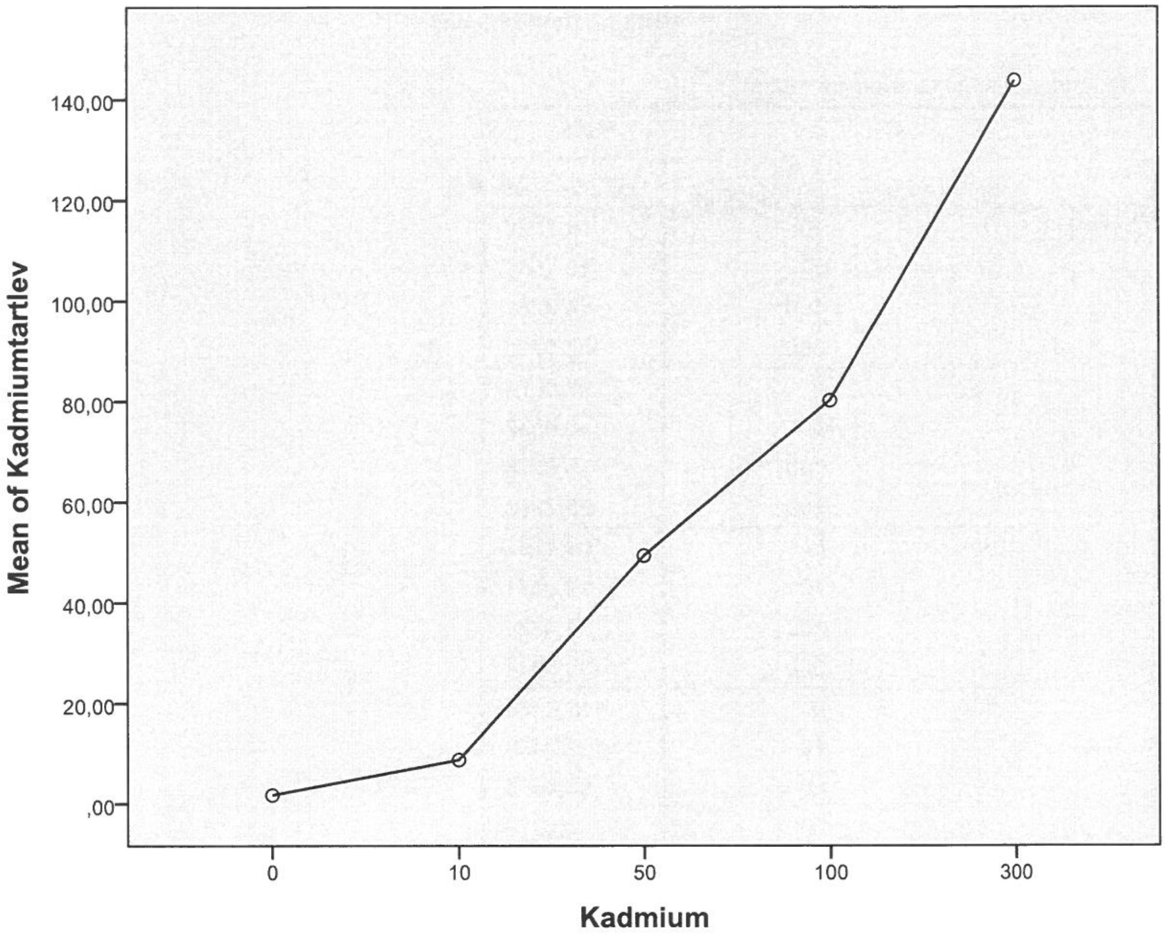

140,00
120,00
100,00
80,00
60,00
40,00
20,00
,00
Mean of Kadmiumtartlev
0
10
50
100
300
Kadmium
