# Supplementary material for: Effect of cadmium stress on certain physiological parameters, antioxidative enzyme activities and biophoton emission of leaves in barley (Hordeum vulgare L.) seedlings
Source: PLoS One. 2020 Nov 3;15(11):e0240470. doi: 10.1371/journal.pone.0240470 (PMC7608874; doi:10.1371/journal.pone.0240470)
Supplement: S1 File — (ZIP) [file pone.0240470.s003.zip › stat results Cd AA leaf 1-3-7 days.pdf]

```
ONEWAY AA1 AA3 AA7 BY Időkezelés
  /MISSING ANALYSIS
  /POSTHOC=TUKEY DUNCAN ALPHA(0.05).
```

Oneway

[DataSet2]

ANOVA

		Sum of Squares	df	Mean Square	F	Sig.
AA1	Between Groups	,473	4	,118	19,911	,003
	Within Groups	,030	5	,006		
	Total	,502	9			
AA3	Between Groups	,123	4	,031	24,577	,002
	Within Groups	,006	5	,001		
	Total	,129	9			
AA7	Between Groups	1,070	4	,268	3,448	,104
	Within Groups	,388	5	,078		
	Total	1,458	9			

Post Hoc Tests

Multiple Comparisons

Dependent Variable		(I) Időkezelés	(J) Időkezelés	Mean Difference (I-J)	Std. Error	Sig.
AA1	Tukey HSD	0	10	-,02855	,07704	,995
			50	-,08000	,07704	,829
			100	-,22515	,07704	,147
			300	-,59125*	,07704	,003
		10	0	,02855	,07704	,995
			50	-,05145	,07704	,955
			100	-,19660	,07704	,216
			300	-,56270*	,07704	,004
		50	0	,08000	,07704	,829
			10	,05145	,07704	,955
			100	-,14515	,07704	,426
			300	-,51125*	,07704	,006
		100	0	,22515	,07704	,147
			10	,19660	,07704	,216
			50	,14515	,07704	,426
			300	-,36610*	,07704	,026
		300	0	,59125*	,07704	,003
			10	,56270*	,07704	,004
			50	,51125*	,07704	,006
			100	,36610*	,07704	,026

Multiple Comparisons

Dependent Variable		(I) Időkezelés	(J) Időkezelés	95% Confidence Interval Lower Bound	95% Confidence Interval Upper Bound
AA1	Tukey HSD	0	10	-,3376	,2805
			50	-,3891	,2291
			100	-,5342	,0839
			300	-,9003	-,2822
		10	0	-,2805	,3376
			50	-,3605	,2576
			100	-,5057	,1125
			300	-,8718	-,2536
		50	0	-,2291	,3891
			10	-,2576	,3605
			100	-,4542	,1639
			300	-,8203	-,2022
		100	0	-,0839	,5342
			10	-,1125	,5057
			50	-,1639	,4542
			300	-,6752	-,0570
		300	0	,2822	,9003
			10	,2536	,8718
			50	,2022	,8203
			100	,0570	,6752

Multiple Comparisons

Dependent Variable		(I) Időkezelés	(J) Időkezelés	Mean Difference (I-J)	Std. Error	Sig.
AA3	Tukey HSD	0	10	,09375	,03534	,194
			50	-,05740	,03534	,542
			100	-,16025*	,03534	,031
			300	-,21630*	,03534	,009
		10	0	-,09375	,03534	,194
			50	-,15115*	,03534	,039
			100	-,25400*	,03534	,004
			300	-,31005*	,03534	,002
		50	0	,05740	,03534	,542
			10	,15115*	,03534	,039
			100	-,10285	,03534	,149
			300	-,15890*	,03534	,032
		100	0	,16025*	,03534	,031
			10	,25400*	,03534	,004
			50	,10285	,03534	,149
			300	-,05605	,03534	,560
		300	0	,21630*	,03534	,009
			10	,31005*	,03534	,002
			50	,15890*	,03534	,032
			100	,05605	,03534	,560
AA7	Tukey HSD	0	10	-,05600	,27858	,999
			50	-,43125	,27858	,579
			100	-,41610	,27858	,606
			300	-,91225	,27858	,102
		10	0	,05600	,27858	,999
			50	-,37525	,27858	,680
			100	-,36010	,27858	,707
			300	-,85625	,27858	,126
		50	0	,43125	,27858	,579
			10	,37525	,27858	,680
			100	,01515	,27858	1,000
			300	-,48100	,27858	,494
		100	0	,41610	,27858	,606
			10	,36010	,27858	,707
			50	-,01515	,27858	1,000
			300	-,49615	,27858	,470
		300	0	,91225	,27858	,102
			10	,85625	,27858	,126
			50	,48100	,27858	,494
			100	,49615	,27858	,470

Multiple Comparisons

Dependent Variable		(I) Időkezelés	(J) Időkezelés	95% Confidence Interval	
				Lower Bound	Upper Bound
AA3	Tukey HSD	0	10	-,0480	,2355
			50	-,1992	,0844
			100	-,3020	-,0185
			300	-,3581	-,0745
		10	0	-,2355	,0480
			50	-,2929	-,0094
			100	-,3958	-,1122
			300	-,4518	-,1683
		50	0	-,0844	,1992
			10	,0094	,2929
			100	-,2446	,0389
			300	-,3007	-,0171
		100	0	,0185	,3020
			10	,1122	,3958
			50	-,0389	,2446
			300	-,1978	,0857
		300	0	,0745	,3581
			10	,1683	,4518
			50	,0171	,3007
			100	-,0857	,1978
AA7	Tukey HSD	0	10	-1,1735	1,0615
			50	-1,5488	,6863
			100	-1,5336	,7014
			300	-2,0298	,2053
		10	0	-1,0615	1,1735
			50	-1,4928	,7423
			100	-1,4776	,7574
			300	-1,9738	,2613
		50	0	-,6863	1,5488
			10	-,7423	1,4928
			100	-1,1024	1,1327
			300	-1,5985	,6365
		100	0	-,7014	1,5336
			10	-,7574	1,4776
			50	-1,1327	1,1024
			300	-1,6137	,6214
		300	0	-,2053	2,0298
			10	-,2613	1,9738
			50	-,6365	1,5985
			100	-,6214	1,6137

*. The mean difference is significant at the 0.05 level.

Homogeneous Subsets

AA1

	Időkezelés	N	Subset for alpha = 0.05		
			1	2	3
Tukey HSD[a]	0	2	,5619		
	10	2	,5904		
	50	2	,6419		
	100	2	,7870		
	300	2		1,1531	
	Sig.		,147	1,000	
Duncan[a]	0	2	,5619		
	10	2	,5904	,5904	
	50	2	,6419	,6419	
	100	2		,7870	
	300	2			1,1531
	Sig.		,358	,056	1,000

Means for groups in homogeneous subsets are displayed.

a. Uses Harmonic Mean Sample Size = 2,000.

AA3

	Időkezelés	N	Subset for alpha = 0.05			
			1	2	3	4
Tukey HSD[a]	10	2	,6722			
	0	2	,7659	,7659		
	50	2		,8233	,8233	
	100	2			,9262	,9262
	300	2				,9822
	Sig.		,194	,542	,149	,560
Duncan[a]	10	2	,6722			
	0	2		,7659		
	50	2		,8233		
	100	2			,9262	
	300	2			,9822	
	Sig.		1,000	,165	,174	

Means for groups in homogeneous subsets are displayed.

a. Uses Harmonic Mean Sample Size = 2,000.

AA7

	Időkezelés	N	Subset for alpha = 0.05	
			1	2
Tukey HSD[a]	0	2	,8805	
	10	2	,9365	
	100	2	1,2966	
	50	2	1,3117	
	300	2	1,7927	
	Sig.		,102	
Duncan[a]	0	2	,8805	
	10	2	,9365	
	100	2	1,2966	1,2966
	50	2	1,3117	1,3117
	300	2		1,7927
	Sig.		,195	,144

Means for groups in homogeneous subsets are displayed.

a. Uses Harmonic Mean Sample Size = 2,000.

```
SAVE OUTFILE='\\srv-fs01\home\jocsak.ildiko\Jócsák\01 Növényélettan\árpa v
izsgálatok\PhD téma '+
    'folytatása\Visi É árpa c vit meghatározás\aszkorbinsav mg-g fr tömeg.
sav'
  /COMPRESSED.
DATASET ACTIVATE DataSet1.
DATASET CLOSE DataSet2.
```
