# Supplementary material for: Effect of cadmium stress on certain physiological parameters, antioxidative enzyme activities and biophoton emission of leaves in barley (Hordeum vulgare L.) seedlings
Source: PLoS One. 2020 Nov 3;15(11):e0240470. doi: 10.1371/journal.pone.0240470 (PMC7608874; doi:10.1371/journal.pone.0240470)
Supplement: S1 File — (ZIP) [file pone.0240470.s003.zip › stat results Cd-1 day AA leaf.pdf]

```
ONEWAY AA1 BY Kadmium
  /STATISTICS DESCRIPTIVES HOMOGENEITY
  /MISSING ANALYSIS
  /POSTHOC=DUNCAN T2 ALPHA(0.05).
```

Oneway

[DataSet2] H:\Jócsák\01 Növényélettan\árpa vizsgálatok\PhD téma folytatása \Visi É árpa c vit meghatározás\aszkorbinsav mg-g fr tömeg.sav

Descriptives

AA1

	N	Mean	Std. Deviation	Std. Error	95% Confidence Interval for Mean	
					Lower Bound	Upper Bound
0	2	,5619	,16949	,11985	-,9610	2,0847
10	2	,5904	,00057	,00040	,5853	,5955
50	2	,6419	,01351	,00955	,5205	,7632
100	2	,7870	,02036	,01440	,6040	,9700
300	2	1,1531	,01881	,01330	,9841	1,3221
Total	10	,7468	,23628	,07472	,5778	,9159

Descriptives

AA1

	Minimum	Maximum
0	,44	,68
10	,59	,59
50	,63	,65
100	,77	,80
300	1,14	1,17
Total	,44	1,17

Test of Homogeneity of Variances

AA1

Levene Statistic	df1	df2	Sig.
.	4	.	.

ANOVA

AA1

	Sum of Squares	df	Mean Square	F	Sig.
Between Groups	,473	4	,118	19,911	,003
Within Groups	,030	5	,006		
Total	,502	9			

Post Hoc Tests

Multiple Comparisons

Dependent Variable: AA1

	(I) Kadmium	(J) Kadmium	Mean Difference (I-J)	Std. Error	Sig.	95% ... Lower Bound
Tamhane	0	10	-,02855	,11985	1,000	-14,9400
		50	-,08000	,12023	1,000	-14,1970
		100	-,22515	,12071	,975	-13,4188
		300	-,59125	,12059	,733	-14,0180
	10	0	,02855	,11985	1,000	-14,8829
		50	-,05145	,00956	,710	-1,2217
		100	-,19660	,01441	,378	-1,9763
		300	-,56270	,01331	,140	-2,2046
	50	0	,08000	,12023	1,000	-14,0370
		10	,05145	,00956	,710	-1,1188
		100	-,14515	,01728	,190	-,4730
		300	-,51125*	,01637	,017	-,7919
	100	0	,22515	,12071	,975	-12,9685
		10	,19660	,01441	,378	-1,5831
		50	,14515	,01728	,190	-,1827
		300	-,36610*	,01960	,029	-,6426
	300	0	,59125	,12059	,733	-12,8355
		10	,56270	,01331	,140	-1,0792
		50	,51125*	,01637	,017	,2306
		100	,36610*	,01960	,029	,0896

Multiple Comparisons

Dependent Variable: AA1

	(I) Kadmium	(J) Kadmium	95% ... Upper Bound
Tamhane	0	10	14,8829
		50	14,0370
		100	12,9685
		300	12,8355
	10	0	14,9400
		50	1,1188
		100	1,5831
		300	1,0792
	50	0	14,1970
		10	1,2217
		100	,1827
		300	-,2306
	100	0	13,4188
		10	1,9763
		50	,4730
		300	-,0896
	300	0	14,0180
		10	2,2046
		50	,7919
		100	,6426

*. The mean difference is significant at the 0.05 level.

Homogeneous Subsets

AA1

	Kadmium	N	Subset for alpha = 0.05 1	2	3
Duncan[a]	0	2	,5619		
	10	2	,5904	,5904	
	50	2	,6419	,6419	
	100	2		,7870	
	300	2			1,1531
	Sig.		,358	,056	1,000

Means for groups in homogeneous subsets are displayed.

a. Uses Harmonic Mean Sample Size = 2,000.
