# Supplementary material for: Effect of cadmium stress on certain physiological parameters, antioxidative enzyme activities and biophoton emission of leaves in barley (Hordeum vulgare L.) seedlings
Source: PLoS One. 2020 Nov 3;15(11):e0240470. doi: 10.1371/journal.pone.0240470 (PMC7608874; doi:10.1371/journal.pone.0240470)
Supplement: S1 File — (ZIP) [file pone.0240470.s003.zip › stat results Cd-1 day SPAD leaf.pdf]

```
ONEWAY SPAD BY Kezelés
  /STATISTICS DESCRIPTIVES HOMOGENEITY
  /PLOT MEANS
  /MISSING ANALYSIS
  /POSTHOC=DUNCAN T2 ALPHA(0.05).
```

Oneway

[DataSet1] H:\Jócsák\01 Növényélettan\árpa vizsgálatok\PhD téma folytatása \SPAD\SPAD-two-way-anova.sav

Descriptives

SPAD

	N	Mean	Std. Deviation	Std. Error	95% Confidence Interval for Mean Lower Bound	95% Confidence Interval for Mean Upper Bound
0	100	29,5343	3,47909	,34791	28,8440	30,2246
10	100	28,4333	3,35001	,33500	27,7686	29,0980
50	100	28,5730	4,32983	,43298	27,7139	29,4321
100	100	27,5410	2,61758	,26176	27,0216	28,0604
300	100	28,3148	3,61216	,36122	27,5981	29,0315
Total	500	28,4793	3,56405	,15939	28,1661	28,7924

Descriptives

SPAD

	Minimum	Maximum
0	22,10	38,90
10	22,10	38,90
50	22,10	55,80
100	22,10	38,90
300	20,20	38,90
Total	20,20	55,80

Test of Homogeneity of Variances

SPAD

Levene Statistic	df1	df2	Sig.
2,043	4	495	,087

ANOVA

SPAD

	Sum of Squares	df	Mean Square	F	Sig.
Between Groups	203,139	4	50,785	4,097	,003
Within Groups	6135,377	495	12,395		
Total	6338,516	499			

Post Hoc Tests

Multiple Comparisons

Dependent Variable: SPAD

	(I) Kezelés	(J) Kezelés	Mean Difference (I-J)	Std. Error	Sig.	95% ... Lower Bound
Tamhane	0	10	1,10100	,48298	,213	-,2664
		50	,96130	,55544	,589	-,6121
		100	1,99330*	,43538	,000	,7596
		300	1,21950	,50152	,148	-,2004
	10	0	-1,10100	,48298	,213	-2,4684
		50	-,13970	,54745	1,000	-1,6907
		100	,89230	,42514	,315	-,3121
		300	,11850	,49265	1,000	-1,2764
	50	0	-,96130	,55544	,589	-2,5347
		10	,13970	,54745	1,000	-1,4113
		100	1,03200	,50596	,356	-,4040
		300	,25820	,56387	1,000	-1,3388
	100	0	-1,99330*	,43538	,000	-3,2270
		10	-,89230	,42514	,315	-2,0967
		50	-1,03200	,50596	,356	-2,4680
		300	-,77380	,44609	,586	-2,0381
	300	0	-1,21950	,50152	,148	-2,6394
		10	-,11850	,49265	1,000	-1,5134
		50	-,25820	,56387	1,000	-1,8552
		100	,77380	,44609	,586	-,4905

Multiple Comparisons

Dependent Variable: SPAD

	(I) Kezelés	(J) Kezelés	95% ... Upper Bound
Tamhane	0	10	2,4684
		50	2,5347
		100	3,2270
		300	2,6394
	10	0	,2664
		50	1,4113
		100	2,0967
		300	1,5134
	50	0	,6121
		10	1,6907
		100	2,4680
		300	1,8552
	100	0	-,7596
		10	,3121
		50	,4040
		300	,4905
	300	0	,2004
		10	1,2764
		50	1,3388
		100	2,0381

*. The mean difference is significant at the 0.05 level.

Homogeneous Subsets

SPAD

	Kezelés	N	Subset for alpha = 0.05 1	2
Duncan[a]	100	100	27,5410	
	300	100	28,3148	
	10	100	28,4333	
	50	100	28,5730	28,5730
	0	100		29,5343
	Sig.		,058	,054

Means for groups in homogeneous subsets are displayed.

a. Uses Harmonic Mean Sample Size = 100,000.

Means Plots

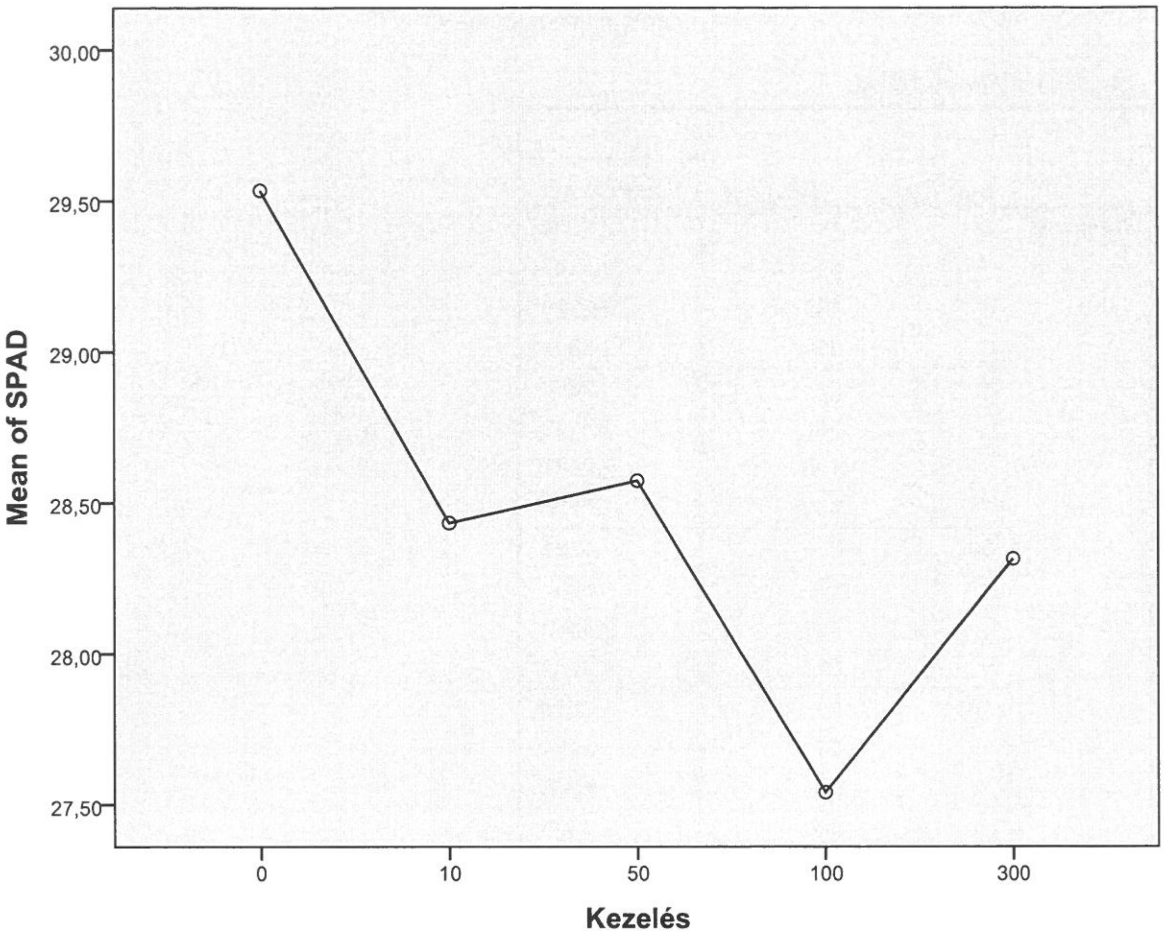
30,00
29,50
29,00
28,50
28,00
27,50
Mean of SPAD
0
10
50
100
300
Kezelés
