# Supplementary material for: Effect of cadmium stress on certain physiological parameters, antioxidative enzyme activities and biophoton emission of leaves in barley (Hordeum vulgare L.) seedlings
Source: PLoS One. 2020 Nov 3;15(11):e0240470. doi: 10.1371/journal.pone.0240470 (PMC7608874; doi:10.1371/journal.pone.0240470)
Supplement: S1 File — (ZIP) [file pone.0240470.s003.zip › stat results Cd-3 day AA leaf.pdf]

```
ONEWAY AA1 BY Kadmium
  /STATISTICS DESCRIPTIVES HOMOGENEITY
  /MISSING ANALYSIS
  /POSTHOC=DUNCAN T2 ALPHA(0.05).
```

Oneway

[DataSet2] H:\Jócsák\01 Növényélettan\árpa vizsgálatok\PhD téma folytatása \Visi É árpa c vit meghatározás\aszkorbinsav mg-g fr tömeg.sav

Descriptives

AA1

	N	Mean	Std. Deviation	Std. Error	95% Confidence Interval for Mean	
					Lower Bound	Upper Bound
0	2	,7659	,00948	,00670	,6808	,8510
10	2	,6722	,05636	,03985	,1658	1,1785
50	2	,8233	,02885	,02040	,5641	1,0825
100	2	,9262	,00304	,00215	,8988	,9535
300	2	,9822	,04624	,03270	,5667	1,3977
Total	10	,8339	,11975	,03787	,7483	,9196

Descriptives

AA1

	Minimum	Maximum
0	,76	,77
10	,63	,71
50	,80	,84
100	,92	,93
300	,95	1,01
Total	,63	1,01

Test of Homogeneity of Variances

AA1

Levene Statistic	df1	df2	Sig.
.	4	.	.

ANOVA

AA1

	Sum of Squares	df	Mean Square	F	Sig.
Between Groups	,123	4	,031	24,577	,002
Within Groups	,006	5	,001		
Total	,129	9			

Post Hoc Tests

Multiple Comparisons

Dependent Variable: AA1

	(I) Kadmium	(J) Kadmium	Mean Difference (I-J)	Std. Error	Sig.	95% ...
						Lower Bound
Tamhane	0	10	,09375	,04041	,942	-3,8354
		50	-,05740	,02147	,880	-1,2553
		100	-,16025	,00704	,145	-,5644
		300	-,21630	,03338	,588	-3,1257
	10	0	-,09375	,04041	,942	-4,0229
		50	-,15115	,04477	,704	-1,4227
		100	-,25400	,03991	,644	-5,0885
		300	-,31005	,05155	,255	-1,0852
	50	0	,05740	,02147	,880	-1,1405
		10	,15115	,04477	,704	-1,1204
		100	-,10285	,02051	,726	-2,4113
		300	-,15890	,03854	,528	-,9571
	100	0	,16025	,00704	,145	-,2439
		10	,25400	,03991	,644	-4,5805
		50	,10285	,02051	,726	-2,2056
		300	-,05605	,03277	,983	-3,9751
	300	0	,21630	,03338	,588	-2,6931
		10	,31005	,05155	,255	-,4651
		50	,15890	,03854	,528	-,6393
		100	,05605	,03277	,983	-3,8630

Multiple Comparisons

Dependent Variable: AA1

	(I) Kadmium	(J) Kadmium	95% ... Upper Bound
Tamhane	0	10	4,0229
		50	1,1405
		100	,2439
		300	2,6931
	10	0	3,8354
		50	1,1204
		100	4,5805
		300	,4651
	50	0	1,2553
		10	1,4227
		100	2,2056
		300	,6393
	100	0	,5644
		10	5,0885
		50	2,4113
		300	3,8630
	300	0	3,1257
		10	1,0852
		50	,9571
		100	3,9751

Homogeneous Subsets

AA1

	Kadmium	N	Subset for alpha = 0.05		
			1	2	3
Duncan[a]	10	2	,6722		
	0	2		,7659	
	50	2		,8233	
	100	2			,9262
	300	2			,9822
	Sig.		1,000	,165	,174

Means for groups in homogeneous subsets are displayed.

a. Uses Harmonic Mean Sample Size = 2,000.
