# Supplementary material for: Effect of cadmium stress on certain physiological parameters, antioxidative enzyme activities and biophoton emission of leaves in barley (Hordeum vulgare L.) seedlings
Source: PLoS One. 2020 Nov 3;15(11):e0240470. doi: 10.1371/journal.pone.0240470 (PMC7608874; doi:10.1371/journal.pone.0240470)
Supplement: S1 File — (ZIP) [file pone.0240470.s003.zip › stat results Cd-3 day SPAD leaf.pdf]

```
ONEWAY SPAD BY Kezelés
  /STATISTICS DESCRIPTIVES HOMOGENEITY
  /PLOT MEANS
  /MISSING ANALYSIS
  /POSTHOC=DUNCAN T2 ALPHA(0.05).
```

Oneway

[DataSet1] H:\Jócsák\01 Növényélettan\árpa vizsgálatok\PhD téma folytatása \SPAD\SPAD-two-way-anova.sav

Descriptives

SPAD

	N	Mean	Std. Deviation	Std. Error	95% Confidence Interval for Mean	
					Lower Bound	Upper Bound
0	100	27,2570	3,76647	,37665	26,5097	28,0043
10	100	24,9810	5,42502	,54250	23,9046	26,0574
50	100	21,0590	5,40011	,54001	19,9875	22,1305
100	100	20,9250	4,71684	,47168	19,9891	21,8609
300	100	21,0210	4,96528	,49653	20,0358	22,0062
Total	500	23,0486	5,52836	,24724	22,5628	23,5344

Descriptives

SPAD

	Minimum	Maximum
0	19,10	36,60
10	10,30	35,30
50	8,50	32,10
100	11,00	30,20
300	5,60	32,50
Total	5,60	36,60

Test of Homogeneity of Variances

SPAD

Levene Statistic	df1	df2	Sig.
4,019	4	495	,003

ANOVA

SPAD

	Sum of Squares	df	Mean Square	F	Sig.
Between Groups	3402,415	4	850,604	35,536	,000
Within Groups	11848,414	495	23,936		
Total	15250,829	499			

Post Hoc Tests

Multiple Comparisons

Dependent Variable: SPAD

	(I) Kezelés	(J) Kezelés	Mean Difference (I-J)	Std. Error	Sig.	95% ... Lower Bound
Tamhane	0	10	2,27600*	,66043	,007	,4036
		50	6,19800*	,65839	,000	4,3315
		100	6,33200*	,60361	,000	4,6221
		300	6,23600*	,62322	,000	4,4701
	10	0	-2,27600*	,66043	,007	-4,1484
		50	3,92200*	,76545	,000	1,7548
		100	4,05600*	,71888	,000	2,0203
		300	3,96000*	,73542	,000	1,8777
	50	0	-6,19800*	,65839	,000	-8,0645
		10	-3,92200*	,76545	,000	-6,0892
		100	,13400	,71701	1,000	-1,8964
		300	,03800	,73359	1,000	-2,0391
	100	0	-6,33200*	,60361	,000	-8,0419
		10	-4,05600*	,71888	,000	-6,0917
		50	-,13400	,71701	1,000	-2,1644
		300	-,09600	,68485	1,000	-2,0350
	300	0	-6,23600*	,62322	,000	-8,0019
		10	-3,96000*	,73542	,000	-6,0423
		50	-,03800	,73359	1,000	-2,1151
		100	,09600	,68485	1,000	-1,8430

Multiple Comparisons

Dependent Variable: SPAD

	(I) Kezelés	(J) Kezelés	95% ... Upper Bound
Tamhane	0	10	4,1484
		50	8,0645
		100	8,0419
		300	8,0019
	10	0	-,4036
		50	6,0892
		100	6,0917
		300	6,0423
	50	0	-4,3315
		10	-1,7548
		100	2,1644
		300	2,1151
	100	0	-4,6221
		10	-2,0203
		50	1,8964
		300	1,8430
	300	0	-4,4701
		10	-1,8777
		50	2,0391
		100	2,0350

*. The mean difference is significant at the 0.05 level.

Homogeneous Subsets

SPAD

	Kezelés	N	Subset for alpha = 0.05: 1	2	3
Duncan[a]	100	100	20,9250		
	300	100	21,0210		
	50	100	21,0590		
	10	100		24,9810	
	0	100			27,2570
	Sig.		,857	1,000	1,000

Means for groups in homogeneous subsets are displayed.

a. Uses Harmonic Mean Sample Size = 100,000.

Means Plots

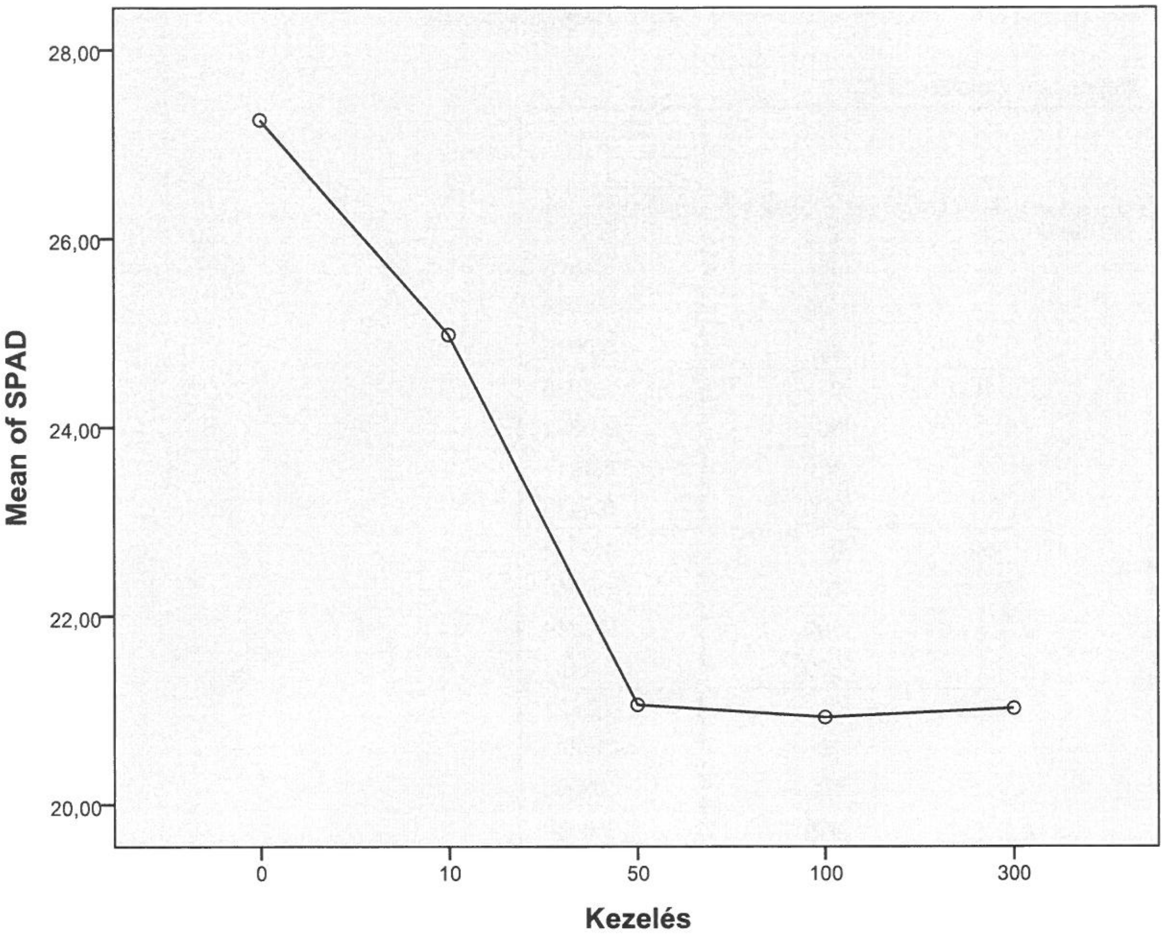
28,00
26,00
24,00
22,00
20,00
Mean of SPAD
0
10
50
100
300
Kezelés
