# Supplementary material for: Effect of cadmium stress on certain physiological parameters, antioxidative enzyme activities and biophoton emission of leaves in barley (Hordeum vulgare L.) seedlings
Source: PLoS One. 2020 Nov 3;15(11):e0240470. doi: 10.1371/journal.pone.0240470 (PMC7608874; doi:10.1371/journal.pone.0240470)
Supplement: S1 File — (ZIP) [file stat results Cd-7 day AA leaf.pdf › stat results Cd-7 day AA leaf.pdf]

```
ONEWAY AA1 BY Kadmium
  /STATISTICS DESCRIPTIVES HOMOGENEITY
  /MISSING ANALYSIS
  /POSTHOC=DUNCAN T2 ALPHA(0.05).
```

Oneway

[DataSet2] H:\Jócsák\01 Növényélettan\árpa vizsgálatok\PhD téma folytatása \Visi É árpa c vit meghatározás\aszkorbinsav mg-g fr tömeg.sav

Descriptives

AA1

	N	Mean	Std. Deviation	Std. Error	95% Confidence Interval for Mean	
					Lower Bound	Upper Bound
0	2	,8805	,36678	,25935	-2,4149	4,1758
10	2	,9365	,31742	,22445	-1,9155	3,7884
50	2	1,3117	,20549	,14530	-,5345	3,1579
100	2	1,2966	,22691	,16045	-,7422	3,3353
300	2	1,7927	,24296	,17180	-,3902	3,9756
Total	10	1,2436	,40254	,12730	,9556	1,5315

Descriptives

AA1

	Minimum	Maximum
0	,62	1,14
10	,71	1,16
50	1,17	1,46
100	1,14	1,46
300	1,62	1,96
Total	,62	1,96

Test of Homogeneity of Variances

AA1

Levene Statistic	df1	df2	Sig.
.	4	.	.

ANOVA

AA1

	Sum of Squares	df	Mean Square	F	Sig.
Between Groups	1,070	4	,268	3,448	,104
Within Groups	,388	5	,078		
Total	1,458	9			

Post Hoc Tests

Multiple Comparisons

Dependent Variable: AA1

	(I) Kadmium	(J) Kadmium	Mean Difference (I-J)	Std. Error	Sig.	95% ...
						Lower Bound
Tamhane	0	10	-,05600	,34299	1,000	-5,0347
		50	-,43125	,29728	,977	-7,7123
		100	-,41610	,30497	,981	-6,8113
		300	-,91225	,31109	,712	-6,8239
	10	0	,05600	,34299	1,000	-4,9227
		50	-,37525	,26738	,977	-5,6223
		100	-,36010	,27590	,983	-5,1134
		300	-,85625	,28265	,658	-5,3747
	50	0	,43125	,29728	,977	-6,8498
		10	,37525	,26738	,977	-4,8718
		100	,01515	,21646	1,000	-3,0592
		300	-,48100	,22501	,844	-3,7932
	100	0	,41610	,30497	,981	-5,9791
		10	,36010	,27590	,983	-4,3932
		50	-,01515	,21646	1,000	-3,0895
		300	-,49615	,23507	,845	-3,8007
	300	0	,91225	,31109	,712	-4,9994
		10	,85625	,28265	,658	-3,6622
		50	,48100	,22501	,844	-2,8312
		100	,49615	,23507	,845	-2,8084

Multiple Comparisons

Dependent Variable: AA1

	(I) Kadmium	(J) Kadmium	95% ... Upper Bound
Tamhane	0	10	4,9227
		50	6,8498
		100	5,9791
		300	4,9994
	10	0	5,0347
		50	4,8718
		100	4,3932
		300	3,6622
	50	0	7,7123
		10	5,6223
		100	3,0895
		300	2,8312
	100	0	6,8113
		10	5,1134
		50	3,0592
		300	2,8084
	300	0	6,8239
		10	5,3747
		50	3,7932
		100	3,8007

Homogeneous Subsets

AA1

	Kadmium	N	Subset for alpha = 0.05: 1	2
Duncan[a]	0	2	,8805	
	10	2	,9365	
	100	2	1,2966	1,2966
	50	2	1,3117	1,3117
	300	2		1,7927
	Sig.		,195	,144

Means for groups in homogeneous subsets are displayed.

a. Uses Harmonic Mean Sample Size = 2,000.
